# Effects of the Bark Resin Extract of *Garcinia nigrolineata* on Chronic Stress-Induced Memory Deficit in Mice Model and the In Vitro Monoamine Oxidases and β-Amyloid Aggregation Inhibitory Activities of Its Prenylated Xanthone Constituents

**DOI:** 10.3390/molecules27093014

**Published:** 2022-05-07

**Authors:** Charinya Khamphukdee, Ibrahim Turkmani, Yutthana Chotritthirong, Yaowared Chulikhit, Chantana Boonyarat, Nazim Sekeroglu, Artur M. S. Silva, Orawan Monthakantirat, Anake Kijjoa

**Affiliations:** 1 Division of Pharmacognosy and Toxicology, Faculty of Pharmaceutical Sciences, Khon Kaen University, Khon Kaen 40002, Thailand; charkh@kku.ac.th; 2 ICBAS-Instituo de Ciências Biomédicas Abel Salazar and CIIMAR, Universidade do Porto, Rua de Jorge Viterbo Ferreira 228, 4050-313 Porto, Portugal; ibrahimturkmani94@gmail.com; 3 Graduate School of Pharmaceutical Sciences, Khon Kaen University, Khon Kaen 40002, Thailand; yutthana_ch@kkumail.com; 4 Division of Pharmaceutical Chemistry, Faculty of Pharmaceutical Sciences, Khon Kaen University, Khon Kaen 40002, Thailand; yaosum@kku.ac.th (Y.C.); chaboo@kku.ac.th (C.B.); 5 Phytotherapy, Medicinal and Aromatic Plants Application & Research Center and Biology Department, Faculty of Arts and Science, Gaziantep University, 27310 Gaziantep, Turkey; nsekeroglu@gmail.com; 6 Departamento de Química & QOPNA, Universidade de Aveiro, 3810-193 Aveiro, Portugal; artur.silva@ua.pt

**Keywords:** *Garcinia nigrolineata*, prenylated xanthones, unpredictable chronic mild stress (UCMS), memory deficit, monoamine oxidases, β-amyloid aggregation

## Abstract

The present study describes investigation of the effects of the bark resin extract of *Garcinia nigrolineata* (Clusiaceae) on the cognitive function and the induction of oxidative stress in both frontal cortex and hippocampus by unpredictable chronic mild stress (UCMS). By using behavioral mouse models, i.e., the Y-maze test, the Novel Object Recognition Test (NORT), and the Morris Water Maze Test (MWMT), it was found that the negative impact of repeated mild stress-induced learning and memory deficit through brain oxidative stress in the UCMS mice was reversed by treatment with the bark resin extract *G. nigrolineata*. Moreover, the prenylated xanthones *viz.* cowagarcinone C, cowaxanthone, α-mangostin, cowaxanthone B, cowanin, fuscaxanthone A, fuscaxanthone B, xanthochymusxanthones A, 7-*O*-methylgarcinone E, and cowagarcinone A, isolated from the bark resin of *G. nigrolineata*, were assayed for their inhibitory activities against β-amyloid (Aβ) aggregation and monoamine oxidase enzymes (MAOs).

## 1. Introduction

In a rapidly evolving society, mental health problems such as anxiety, depression, and memory problems are becoming increasingly widespread among the general population, and regular exposure to stressful situations can lead to accelerated aging and a variety of memory problems [1]. Accumulated evidence demonstrates that the secretion of adrenal hormones and monoaminergic neurotransmitters in response to stressful moments can be considered as a major modulator of learning and memory processes in humans and rodents [2]. Although the mechanism underlying this pathway is not fully understood, many reports indicated that a decrease in serotonin (5-HT) neurotransmitter in the frontal cortex and hippocampus is involved in chronic stress [3]. In connection with this event are the monoamine oxidase enzymes (MAOs), which are found in the outer membrane of the mitochondria of neurons, and exist as two isoenzymes, MAO-A and MAO-B. MAO-A is thought to play a major role in degrading serotonin, epinephrine, norepinephrine, and melatonin, whereas MAO-B is responsible for the degradation of dopamine, phenylethylamine, and benzylamine [4]. As a result, MAOs are important in nerve transmission regulation, and changes in their activities caused by therapeutic intervention are likely to have a significant impact on behavior [5]. Furthermore, several studies reported that different stress paradigms cause changes in not only the level of monoamine neurotransmitters in various brain regions in rodents but also antioxidant defenses [6]. Oxidative stress is a relatively severe form of cognitive impairment, since the brain is more susceptible to oxidative damage due to overproduction of free radicals, relative to other organs, because of its high fatty acid content, leading to a high concentration of peroxidizable lipids [1,2,6]. In addition, oxidative stress has been linked to the etiology of Alzheimer’s disease (AD) through β-amyloid (Aβ), a hallmark of this disease, by increasing Aβ deposition, while Aβ also induces oxidative stress, resulting in neuronal damage [7].

Plants of the genus *Garcinia* (Family Clusiaceae) are used in many Ayurvedic preparations for treatment of various illnesses, since they contain a wide range of biologically active metabolites [8]. Thus, *Garcinia* species have gained popularity in recent decades due to the chemical constituents of their extracts, which have been shown to exhibit beneficial effects in many diseases [9]. The constituents of *Garcinia* species display a myriad of biological and pharmacological effects, such as antioxidant, antitumor, anticancer, antihistamine, antiulcer, antiviral, antimicrobial, vasodilatory, cardioprotective, nephroprotective, hepatoprotective, anti-obesity, anti-inflammatory, and stress-relieving activities [10]. With over 400 species, *Garcinia* is the largest genus in the Clusiaceae family. It is particularly abundant in tropical and subtropical regions of the world, with 29 species found in Thailand. *Garcinia nigrolineata* Planch. ex T. Anderson is a small- to medium-sized tree that is found throughout Southeast Asia [11].

Chemical investigations of different parts, i.e., bark [12], leaves [13], and twigs [14], from the same plant of *G. nigrolineata,* collected in June 2000 from Southern Thailand, revealed that they contained different compounds. More recently, chemical study of leaves of *G. nigrolineata*, collected in January 2018 in Northern Thailand [11] also resulted in the isolation of different constituents from those isolated from leaves of *G. nigrolineata*, collected in Southern Thailand [13]. For this reason, we decided to investigate the chemical constituents of the bark resin of *G. nigrolineata*, collected in January 2020 from Khon Kaen, Northeastern Thailand. Since α-mangostin, a prenylated xanthone implicated in the improvement of the spatial learning and memory of chronic cerebral hypoperfusion [15] and attenuation of β-amyloid-oligomer-induced neurotoxicity by inhibiting amyloid aggregation [16], was isolated from this resin extract, we also investigated the anti-dementia activity of the extract of the bark resin of *G. nigrolineata* in the unpredictable chronic mild stress (UCMS) mice model, along with the determination of the oxidative stress through lipid peroxidation in the frontal cortex and hippocampus. Moreover, the effects of the isolated pure compounds were also investigated for their inhibitory activities against MAOs and Aβ aggregation.

## 2. Results

### 2.1. Isolation and Structure Elucidation of the Constituents of the Extract of the Bark Resin of G. nigroleanata

**Figure 1:**
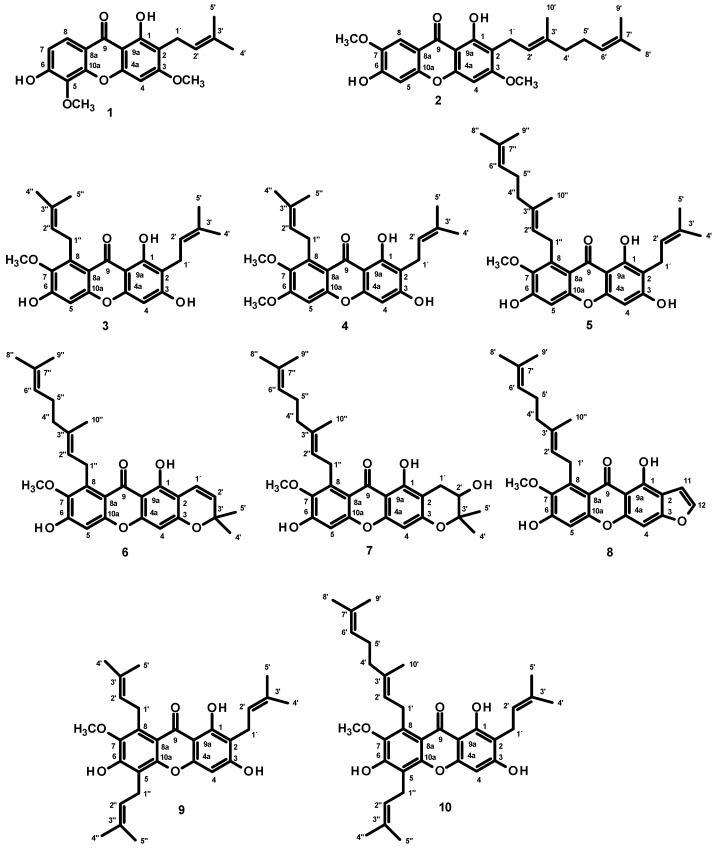
Prenylated xanthones isolated from the extract of the bark resin of *G. nigrolineata* Planch.ex T. Anderson.

Fractionation of the CH_2_Cl_2_ crude extract of the bark resin of *G. nigrolineata* by column chromatography, followed by purification by crystallization, preparative TLC, and Sephadex LH-20 column, led to the isolation of two monoprenylated xanthones, cowagarcinone C (**1**) [17] and cowaxanthone (**2**) [17], three diprenylated xanthones, α-mangostin (**3**) [18], cowaxanthone B (**4**) [19,20], and cowanin (**5**) [20], three modified diprenylated xantnones, fuscaxanthone A (**6**) [17,18], fuscaxanthone B (**7**) [18], xanthochymusxanthone A (**8**) [21], and two triprenylated xanthones, 7-*O*-methylgarcinone E (**9**) [22] and cowagarcinone A (**10**) [17] (Figure 1). The structures of the isolated compounds were elucidated by analysis of 1D and 2D NMR spectra and comparison of their ^1^H and ^13^C NMR chemical shift values (Supplementary materials, Tables S1–S10 and Figures S1–S50) with those from the literature.

### 2.2. Effect of the Bark Resin Crude Extract of G. nigrolineata (GN) on UCMS-Induced Cognitive Deficit-like Behavior

Chronic stress has been recognized to suppress learning and memory over a variety of cognitive tasks [1]. Thus, three behavioral models, *viz.* the Y-maze Test, the Novel Object Recognition Test (NORT), and the Morris Water Maze Test (MWMT), were used to determine whether GN resin extract modulates UCMS-induced cognitive deficit.

**Figure 2:**
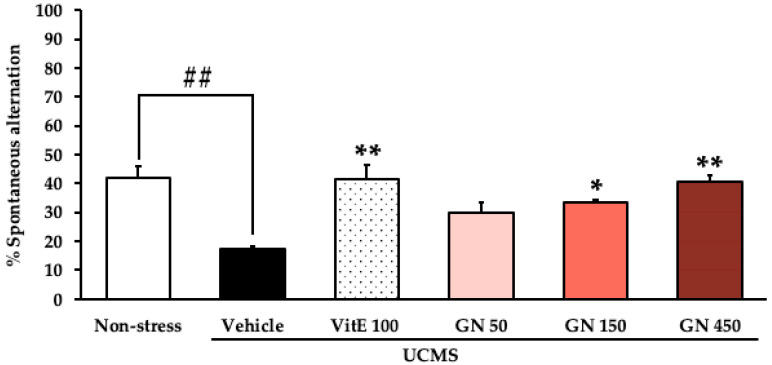
The effect of the GN bark resin extract on learning and memory in the Y-maze test. Each column represents the mean ± SEM (*n* = 10–11). ^##^ *p* < 0.001 vs. the vehicle-treated non-stress group. * *p* < 0.05, ** *p* < 0.001 vs. the vehicle-treated UCMS (post-hoc Tukey test).

When assessed in the Y-maze test, the vehicle-treated UCMS mice showed significantly less percentage of spontaneous alternation than the non-stress group, indicating an impairment of a spatial memory or a short-term memory caused by UCMS. Treatment with GN bark resin extract with two different doses, i.e., 150 and 450 mg/kg/day, and with vitamin E (100 mg/kg/day) significantly increased the percentage of spontaneous alternation compared to the vehicle-treated UCMS group (Figure 2). For detailed statistical analysis, see Table S11.

**Figure 3:**
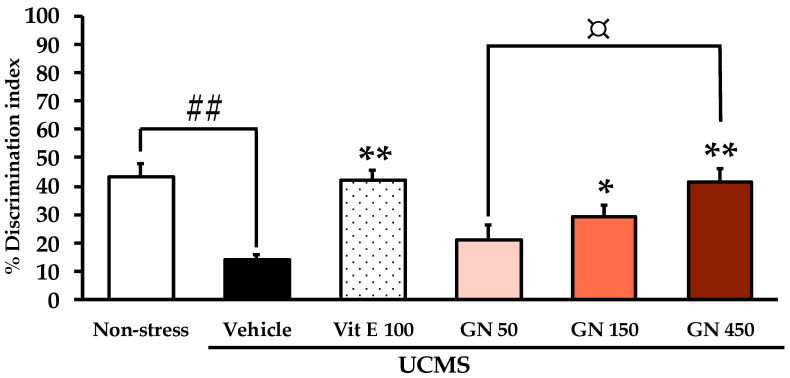
The effect of the GN bark resin extract on learning and memory in the NORT. Each column represents the mean ± SEM (*n* = 10–11). ^##^ *p* < 0.001 vs. the vehicle-treated non-stress group. * *p* < 0.05, ** *p* < 0.001 vs. the vehicle-treated UCMS group and ^¤^ *p* < 0.05 vs. the GN bark resin extract (Post-hoc Tukey test).

For the NORT, during the sample phase trial, all groups spent equal time exploring the two identical objects (for detailed statistical analysis, see Table S12). During the test phase trial of NORT, the non-stress mice could successfully discriminate between the new and the familiar object, while the vehicle-treated UCMS mice failed to recognize the new object. In contrast, the UCMS mice treated with vitamin E (100 mg/kg/day) and GN bark resin extract (150 and 450 mg/kg/day) were significantly ameliorated in the discrimination index performance. A dose-dependent manner of the GN bark resin extract, from 50 to 450 mg/kg/day, was observed in the NORT (Figure 3).

**Figure 4:**
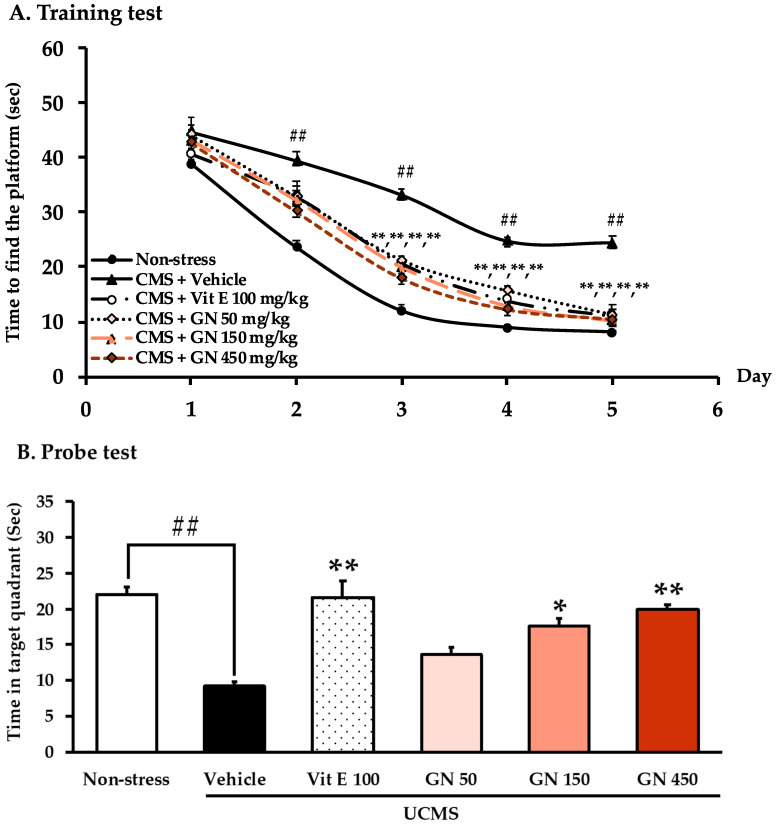
Latency time of training the mouse to find the target platform (*n* = 10–11). Each data point represents the mean ± SEM of latency, 4 trials/day for 5 days (**A**). The effect of the GN bark resin extract on the UCMS-induced cognitive deficit in the MWMT (**B**). Each column represents the mean ± SEM (*n* = 10–11). ^##^ *p* < 0.001 vs. the vehicle-treated non-stress group. * *p* < 0.05, ** *p* < 0.001 vs. the vehicle-treated UCMS group (post-hoc Tukey test).

In the step-through MWMT, results showed that time to find the platform progressively decreased during five days, revealing a gradual improvement of learning of mice. As shown in Figure 4A, this significant effect was observed on day 2 and further potentiated on days 3, 4, and 5 when compared between the non-stress mice and the vehicle-treated UCMS mice. Moreover, three weeks of treatment with GN bark resin extract and vitamin E have significantly reduced escape latency on days 3, 4, and 5 (for detailed statistical analysis, see Table S13.1). As for the probe phase on day 6 (Figure 4B), the results show that the vehicle-treated UCMS mice spent significantly less time in the target quadrant than the non-stress mice, indicating that UCMS causes learning and memory deficit. However, oral administration with vitamin E (100 mg/kg/day) and the GN bark resin extract (150, and 450 mg/kg/day) significantly increased time spent in the target quadrant, indicating an improvement of the cognitive function (for detailed statistical analysis, see Table S13.2).

To avoid the possibility of false positives from the hyper movement of animals that affects the behavioral model test, mice were observed for locomotor activity by counting the total arms of entry during 5 min of the mouse movement. The results showed that there was no significant difference in locomotor activity between experimental groups.

### 2.3. Treatment with the Crude Extract of the Bark Resin of G. nigrolineata (GN) Ameliorated Oxidative Stress in UCMS-Mice

**Figure 5:**
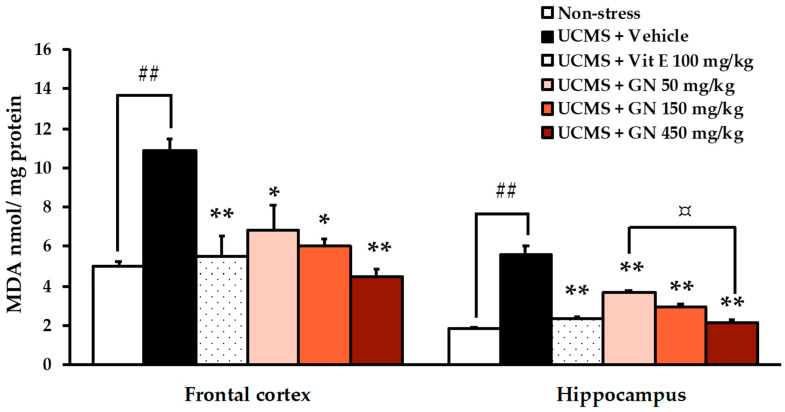
The effect of the GN bark resin extract on the UCMS-induced lipid peroxidation in frontal cortex and hippocampus. Each column represents the mean ± SEM (*n* = 4–5). ^##^ *p* < 0.001 vs. the vehicle-treated non-stress group. * *p* < 0.05, ** *p* < 0.001 vs. the vehicle-treated UCMS group and ^¤^ *p* < 0.05 vs. the GN bark resin extract (post-hoc Tukey test).

Assessing thiobarbituric acid reactive substances (TBARs) is an effective measure to determine oxidative stress through lipid peroxidation [2]. The level of malondialdehyde (MDA), a biomarker of neuronal damage, was found to be higher in both frontal cortex (for detailed statistical analysis, see Table S14.1) and hippocampus (for detailed statistical analysis, see Table S14.2) of the vehicle-treated UCMS-mice when compared to the non-stress mice. However, treatment of the UCMS-mice with a strong antioxidant such as vitamin E (100 mg/kg/day) and three different doses of GN bark resin extract (50, 150, 450 mg/kg/day) provided significant neuroprotection against UCMS-induced lipid peroxidation in both brain regions (Figure 5).

### 2.4. β-Amyloid (Aβ) Aggregation Inhibitory Activity of the Constituents of G. nigrolineata (GN) Bark Resin

**Figure 6:**
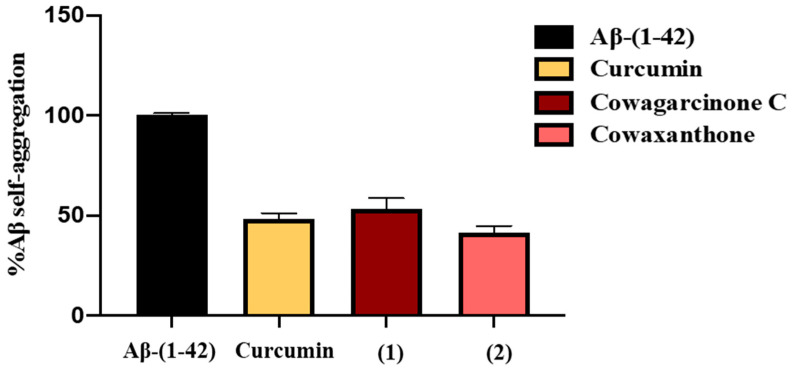
The percent of self-aggregation of Aβ-(1-42) in the presence of curcumin, **1** and **2**. The percent of self-aggregation of Aβ-(1-42) without any compound is taken as 100%.

A thioflavin-T (Th-T) assay was used to evaluate the ability of **1**–**6** and **8**–**10**, isolated from GN bark resin extract, to inhibit Aβ42 aggregation. The presence of Aβ plaques in the brain appears to be related to cognitive decline and neurodegeneration [23]. In this study, curcumin, which has been reported in our previous studies [24], was used as the positive control at a concentration of 5 µM. The results showed that curcumin could inhibit the aggregation of Aβ with a percentage of inhibition value of 51.73 ± 2.89. Among the tested compounds, only **1** and **2**, at 100 µM, showed good Aβ aggregation inhibitory activity with a percentage of inhibition value of 46.79 ± 5.46 and 58.56 ± 3.28, respectively (Table 1). The percent of self-aggregation of Aβ-(1-42) in the presence of curcumin, **1** and **2** are 48.27 ± 2.89, 53.21 ± 5.46, 41.44 ± 3.28, respectively (Figure 6).

### 2.5. MAO-A and MAO-B Inhibitory Activities of the Constituents of G. nigrolineata (GN) Bark Resin

The stress response is influenced by monoaminergic systems. Moreover, mental illness and other intellectual disabilities have been linked to the activity of MAOs. Since MAOs cause a decrease in the levels of monoamine neurotransmitters, inhibition of MAO-A and MAO-B will result in elevated levels of monoamine neurotransmitters [5]. Therefore, **1**–**6** and **8**–**10** (**7** was not tested since we did not have enough quantity) were evaluated for their capacity to inhibit MAO-A and MAO-B. As indicated in Table 1, all the tested compounds showed inhibitory effects against MAOs. In order to compare the selectivity of the compounds on the two isoenzymes, the IC_50_ values were converted to the corresponding enzyme-inhibitor dissociation constant (*Ki*) by the Cheng–Prusoff equation; *Ki* = IC_50_/(1 + [S]/*Km*), and then converted to a corresponding selective index (Si) of enzyme type values according to the equation, Si = *Ki* (MAO-B)/*Ki* (MAO-A), as previously described by Tonge [25]. The results show that **2**, **5**, **8,** and **10** have a selectivity index on MAO-A, indicating that they possess specific antidepressant activity. On the other hand, **1**, **3**, **4**, **6**, and **9** exhibited their selectivity index on MAO-B, indicating their effect of anti-dementia and anti-Alzheimer’s disease.

## 3. Discussion

In today’s society, people thrive on performance, competition, and perfection, leading to an insidious increase in stress in daily life. Chronic mild stress has been demonstrated in a number of studies as one of the leading causes of dementia [1]. Thus, searching for natural compounds from plants used in traditional medicine or food which can counteract the chronic mild stress is an important avenue to improve life conditions.

In spite of being rich sources of xanthone derivatives, only a few species of the genus *Garcinia* have been investigated for their pharmacological effects. One of the most studied species is *G. mangostana L.* (mangosteen). The pericarp extract and its bioactive xanthones have been investigated for their potential therapeutic values in AD, Parkinson’s disease, and depression [26]. Among the prenylated xanthones isolated from *Garcinia* species, α-mangostin was the most investigated and was claimed to have the pivotal neuroinflammatory, therapeutic, and neuroprotective roles [27]. Recently, Tiwari et al. have found that α-mangostin was able to reduce the overactivation of the extracellular signal-regulated kinase (ERK) signaling and also restored autism-like behavioral and neurochemical alterations [28].

The main findings of the current study are that the deleterious effects of unpredictable chronic mild stress to impair the ability of learning and in both short-term and long-term memory, through oxidative stress in the frontal cortex and hippocampus, can be improved by treatment with the extract of the bark resin of *G. nigrolineata*. Moreover, our study revealed that the xanthone constituents of the bark resin of *G. nigroleanata, viz.* **5** inhibited MAO-A and MAO-B enzymes with a high potency, whereas **2** showed a good inhibitory activity on amyloid aggregation.

The UCMS model is well-established for studying animal behavior of stress-related mental illness due to its high predictive power on both behavioral and physiological changes [29]. Since chronic stress induces cognitive dysfunction in various tasks, the effects of the crude extract of the bark resin of *G. nigrolineata* were evaluated by using three different models. First, the recall or short-term memory was assessed by the Y-maze test. The recall memory is measured by allowing mice to explore all three arms of the maze based on their natural desire to explore new areas. A mouse with a good recall memory, i.e., good frontal cortex functions, will recall the arms it has recently visited and will be more likely to enter a less visited arm [30]. Second, short-term recognition memory was evaluated by the Novel Object Recognition Test (NORT). This experiment is based on an intact tendency of mice to spend more time discovering different objects than the familiar one. The recognition process of the UCMS, which results in damage of frontal cortex and hippocampus, is reflected by a low percent discrimination index in this behavior model [31]. Third, long-term learning and memory were determined by the Morris Water Maze Test (MWMT). Spatial learning is evaluated by repeated trials in the training phase, while reference memory is determined by preference for the target quadrant when the platform is removed. The MWMT has proved to be a robust and reliable test which is strongly correlated with *N*-methyl-D-aspartate (NMDA) receptor function and hippocampal synaptic plasticity [32]. In agreement with previous reports, repeated unpredictable chronic mild stress for 6 weeks completely induced cognitive dysfunction with dementia-like behavior models, with decreasing spontaneous alternations in the Y-maze test, failure to discriminate between two different objects in the NORT, and increasing escape latency in the acquisition phase but decreasing time spent in target quadrant in the test day in the MWMT. These effects are in agreement with those observed in previous studies [1,33,34]. Furthermore, we have also observed that the daily administration of both *G. nigrolineata* bark resin extract and vitamin E dose-dependently improved not only the impairment of the frontal cortex-dependent recall memory (Y-maze test) and the deficit in frontal-cortex- and hippocampus-dependent spatial recognition (NORT), but also hippocampal synaptic plasticity and NMDA-dependent long-term learning and memory dysfunction.

UCMS has been shown to influence different regions of brain, i.e., hippocampus and prefrontal cortex [35], which play a critical role in spatial navigation and memory [36]. In addition, it is widely accepted that exposure to chronic stress causes the over-secretion of stress hormone glucocorticoids (GCs), which may trigger the enhancement of cellular metabolic rate and promote ATP synthesis, leading to a spontaneous superoxide anion (O.^−2^) and other radical overproductions. This oxidative stress ultimately induces neuronal cell damage, especially in the pyramidal cell of the hippocampus where glucocorticoids receptors are located, and thus causing the consequences of cognitive and memory decline. [3,7,37]. In agreement with previous studies [38,39], our results showed that levels of MDA, a biomarker of lipid peroxidation caused by oxidative stress, were significantly elevated not only in the frontal cortex but also in the hippocampus of the vehicle-treated UCMS mice when compared with non-stress mice. Nevertheless, the MDA level of both brain regions was reduced in UCMS mice that received the GN bark resin extract at three different doses and vitamin E for 6 weeks. Most importantly, the GN bark resin extract exerts protective effects against memory deficit and brain damage attributable to UCMS, a key risk factor for neurodegenerative disease [40].

In order to better understand the molecular mechanism of GN bark resin extract on cognitive improvement, its chemical constituents, i.e., **1**–**6** and **8**–**10** (Figure 1), were evaluated for in vitro anti-amyloid aggregation and MAO inhibitory effects. As a main constituent of amyloids plaques, the 39–43 amino acids-β-amyloid peptides (Aβ) are thought to play a crucial role in the pathogenesis of AD. The aggregated Aβ, found in senile plaques, is involved in neuronal toxicity and impaired hippocampal long-term potentiation [41]. The inhibitory activity against Aβ-42 self-aggregation of **1**–**6** and **8**–**10** was evaluated using Th-T fluorescence assay. At a concentration of 100 μM, **1** and **2** showed good activity with the percentage of inhibition values at 46.79 ± 5.46 and 58.56 ± 3.28, respectively, whereas the rest of the xanthones tested did not show any anti-Aβ aggregation activity. Curiously, Wang et al. [42] have previously demonstrated that α-mangostin (**3**) attenuated β-amyloid-oligomer-induced neurotoxicity by inhibiting amyloid aggregation. The reasons for this discrepancy may originate from the different experimental protocol used by Wang et al. [42], which used a different incubation time and procedure from ours. To assess the inhibition of aggregation, Wang et al. [42] mixed Aβ (1-42) with α-mangostin (**3**) and incubated them for 6 days at 37 °C. On the contrary, in the present study the incubation time was much shorter, i.e., 24 h. The longer incubation time may influence the binding capacity of the test compound with Aβ (1-42). This notion was supported by Durani et al. who demonstrated that the tocotrienol-rich fraction (TRF), a mixture of vitamin E analogs from palm oil at the concentration of 0.001%, significantly decreased the formation of amyloid aggregation only at 48 h, but not at 0, 6, and 24 h of incubation [43]. Moreover, other compounds in the solution can modulate the signal intensity, either through fluorescence quenching, an inner filter effect, or interactions on the fibril’s surface of Aβ [44].

Finally, MAO inhibitory effects of **1**–**6** and **8**–**10** were investigated. MAO-A and MAO-B are flavin adenine dinucleotide (FAD)-containing enzymes that show different substrate specificity and sensitivity to inhibitors. MAOs play an important role in the metabolism of monoamine neurotransmitters in the central nervous system [4]. Both isozymes play a role in the progression of AD. MAO-B, in particular, has been suggested as a biomarker because it is related to the formation of Aβ peptide, and is linked to cognitive dysfunction and cholinergic neuron disorders [45]. Furthermore, an increase in MAO-A activity in AD is associated with an increase in neurotoxic metabolites such as hydrogen peroxide, and oxidative stress, leading to a neuronal cell death [46]. Thus, both MAO isoforms can be considered as targets for the treatment of AD [4,45]. Interestingly, all of the tested compounds exhibited MAO inhibitory activities; in particular, **5** and **10** displayed a strong inhibitory effect against MAO-A, with IC_50_ values of 7.10 and 3.58 µM, respectively. Compounds **6** and **9** were potent MAO-B inhibitors, with IC_50_ values of 0.07 and 0.40 µM, respectively. These xanthone derivatives may exert their inhibitory effects by means of reversible interaction with the flavin moiety of FAD, which is a redox-active coenzyme factor of MAOs [47]. Interestingly, only **6** exhibits potency and selectivity toward MAO-B, with IC_50_ and *Ki* values less than those of deprenyl, a preferred standard for selective MAO-B inhibitor.

Taken together, the present study demonstrates that *G. nigrolineata* bark resin extract improved the effect of UCMS-induced cognitive deficit in mice by protecting frontal cortex and hippocampal cell damage from oxidative stress and that the effects of *G. nigrolineata* bark resin are mainly due to antioxidant and MAO inhibitory activities, and in lesser extent, the inhibition of Aβ aggregation. This is not surprising since xanthones, which are a main constituent of *G. nigrolineata* bark resin, are strong antioxidants [48].

## 4. Experimental Section

### 4.1. General Experimental Procedures

The melting points were determined on a Stuart Melting Point Apparatus SMP3 (Bibby Sterilin, Stone, Staffordshire, UK) and are uncorrected. Optical rotations were measured on an ADP410 Polarimeter (Bellingham + Stanley Ltd., Tunbridge Wells, Kent, UK). ^1^H and ^13^C NMR spectra were recorded at ambient temperature on a Bruker AMC instrument (Bruker Biosciences Corporation, Billerica, MA, USA) operating at 300 or 500 and 75 or 125 MHz, respectively. Fluorescence intensities were measured using EnSight^®^ Multimode Plate Reader (Perkin Elmer Inc., Waltham, MA, USA). The extraction was performed by a microwave extractor model MS23F300EEW (Samsung, Kuala Lumper, Malaysia). A Merck (Darmstadt, Germany) silica gel GF_254_ was used for preparative TLC, and Merck Si gel 60 (0.2–0.5 mm), Li Chroprep silica gel and Sephadex LH 20 were used for column chromatography.

### 4.2. Plant Material

Dried resin (2.95 kg) of *Garcinia nigrolineata* Planch. ex T. Anderson (Clusiaceae) was collected from its trunk barks in the campus of Khon Kaen University, Khon Kaen, Thailand (GPS coordinates: 16°27′38.2′′ N 102°49′07.3′′ E), in January 2020. The plant material was identified by Dr. Prathan Leucha of the Faculty of Pharmaceutical Sciences of Khon Kaen University, Khon Kaen, Thailand. The voucher specimen (PSKKU-PL-047) was deposited at the Herbarium of the Faculty of Pharmaceutical Sciences, Khon Kaen University.

### 4.3. Extraction and Isolation of the Constituents

The dried resin was extracted by a microwave using 60 L of CH_2_Cl_2_ (60 L), 300 watts, 4 min, at 34 °C. The organic solutions were combined and evaporated under reduced pressure and dried in a desiccator to give 650 g of a dried crude CH_2_Cl_2_ extract. The crude CH_2_Cl_2_ extract (10 g) was applied on a column chromatography of silica gel (110 g) and eluted with mixtures of petrol–CHCl_3_ and CHCl_3_–Me_2_CO, wherein 250 mL fractions (Frs) were collected as follows: Frs 1–100 (CHCl_3_–petrol, 1:1), 101–170 (CHCl_3_–petrol, 7:3), 171–190 (CHCl_3_–petrol, 9:1), 191–268 (CHCl_3_–Me_2_CO, 9:1). Fr. 6–25 were combined (275 mg) and applied on a Sephadex LH-20 column (10 g), and eluted with MeOH to give two subfractions (sfr.): sfr. A (39 mg) and Srf. B (211 mg). Sfr. A (39) was purified by preparative TLC (Silica Gel G_254_, CHCl_3_: Petrol: HCO_2_H, 3:7:0.01) to give 4.6 mg of 7-*O*-methylgarcinone E (**9**) and 5.2 mg of xanthochymusxanthone A (**8**). Sfr. B (211 mg) was applied on a Sephadex LH-20 column (30 g) and eluted with a 1:1 mixture of CHCl_3_-MeOH) to give 55 fractions (frs). Frs 23–46 were combined (49 mg) and applied on another Sephadex LH-20 column (10 g), and eluted with MeOH to give 60 sfrs. Sfrs. 37–42 were combined and evaporated to give 11.9 mg of fuscaxanthone A (**6**). Frs. 47–67 were combined (256 mg) and crystalized in a mixture of CHCl_3_ and petrol to give 130 mg of cowanin (**5**). The mother liquor was purified by preparative TLC (Silica gel G_245_, CHCl_3_: petrol: Me_2_CO, HCO_2_H, 96:3:1:0.01) to give additional 38 mg of cowanin (**5**) and 2.7 mg of fuscaxanthone B (**7**). Frs. 68–100 were combined (427 mg) and applied on a column chromatography of silica gel (30 g), and eluted with mixtures of petrol–CHCl_3_ and CHCl_3_–Me_2_CO, wherein 25 mL sfrs were collected as follows: sfrs 1–10 (CHCl_3_–petrol, 7:3),11–20 (CHCl_3_–petrol, 9:1), 21–40 (CHCl_3_–Me_2_CO, 9:1). Sfrs 4–9 were combined (193.5 mg) and applied over a Sephadex LH-20 column (10 g) and eluted with MeOH to give 35 ssfrs of 10 mL each. Ssfrs 34 and 35 were combined (37.1 mg) and purified by TLC (Silica Gel G_254_, CHCl_3_: petrol: Me_2_CO, HCO_2_H, 94:2:4: 0.01) to give 5.8 mg of α-mangostin (**3**) and 6.6 mg of cowanin (**5**). Frs. 142–156 were combined (99 mg) and crystallized in a mixture of CHCl_3_ and Me_2_CO to give 37.9 mg of cowagarcinone C (**1**). The mother liquor was purified by preparative TLC (Silica Gel G_254_, CHCl_3_: Me_2_CO, HCO_2_H, 95.5:0.5: 0.01) to give additional 1.3 mg of cowagarcinone C (**1**). Frs 157–170 were combined (42.8 mg) and crystallized in a mixture of CHCl_3_ and petrol to give 19.1 mg of cowaxanthone (**2**).

Another portion (5.0 g) of the crude CH_2_Cl_2_ extract was chromatographed on a silica gel column (50 g) and eluted with gradient mixtures of petrol, CHCl_3_ and Me_2_CO, wherein 250 mL were collected as follow: Frs 1–125 (CHCl_3_–petrol, 7:3), 126–294 (CHCl_3_–petrol, 9:1). Frs. 9–23 were combined (1.16 g) and applied over Sephadex LH-20 column (20 g) and eluted with MeOH wherein 15 mL 100 sfrs were collected. Sfrs. 18–79 were combined (1.03 g) and applied over another Sephadex LH-20 column (20 g) and eluted with petrol–CH_2_Cl_2_–MeOH, 1:2:1 to give 136 ssfrs of 15 mL each. Ssfrs 69–119 were combined (241 mg) and applied on another Sephadex LH-20 column (20 g) and elutes with CHCl_3_–MeOH, 1:1 to give 8.4 mg of cowaxanthone B (**4**) and 3.3 mg of cowagarcinone A (**10**). Ssfrs 120–129 were combined (121.3 mg) and recrystallized in MeOH to give 10.3 mg of 7-*O*-methylgarcinone E (**9**).

### 4.4. Animals

Five-week-old male ICR mice (weight: 20–30 g), which were acquired from Nomura Siam International (Pathumwan, Bangkok, Thailand), were maintained at the Animal Unit of the Faculty of Pharmaceutical Sciences, Khon Kaen University. Mice were cared for in accordance with the Guiding Principles for the Care and Use of Animals (NIH Publications No. 80–23, revised in 2011). The facilities sustained a 12 h light/dark cycle, constant temperature (22 ± 2 °C), and humidity (45 ± 2%), with full access to food and drink. The current research was authorized by the Animal Ethics Committee for Use and Care of Khon Kaen University (IACUC-KKU-121/64)

### 4.5. Chronic Unpredictable Stress

Mice in the stress groups received mild stressors for 6 weeks. The serial repetition of stressors to induce unpredictable chronic mild stress (UCMS) included one period of food and water deprivation (18 h), two periods of tilted cage at 45° (2 h), two periods of restricted access to food, 5 micro pellets (1 h), two periods of exposure to empty bottle (3 h), one period of wet cage, 200 mL of water in 100 g sawdust bedding (21 h), two periods of light exposure (36 h), two periods of intermittent sound (3 and 5 h) and two periods of paired caging (2 h). All these stressors were randomly scheduled over one-week periods in day/night time and repeated along with the experiment, whereas the control group (non-stress group) was housed under normal environments [33].

### 4.6. Experimental Design and Drug Treatment

**Figure 7:**
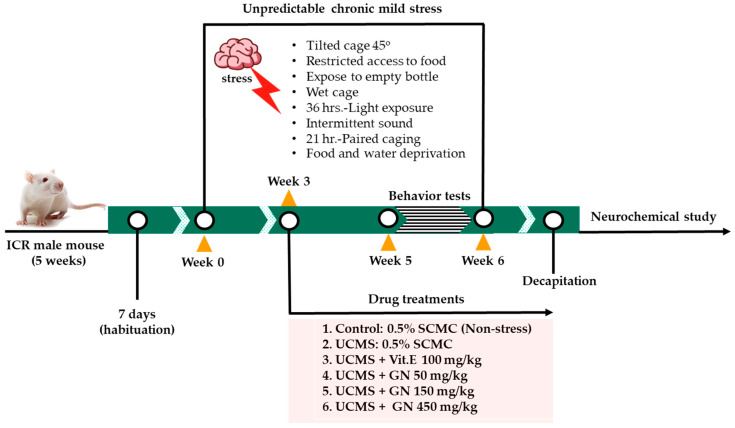
A schematic diagram of the animal experimental design in this study.

The mice were randomly divided into six groups (*n* = 12): (1) non-stress control group that received 0.5% sodium carboxymethylcellulose (SCMC) (HiMedia laboratory, Mumbai, India) 1 mg/kg, *p.o.*, and (2) UCMS group, administered with 0.5% SCMC (1 mg/kg, *p.o*.), (3) UCMS + vitamin E (100 mg/kg, *p.o*.) (Sigma-Aldrich, St. Louis, MO, USA), (4) UCMS + GN (*G. nigrolineata* bark resin extract 50 mg/kg, *p.o*.), (5) UCMS + GN (*G. nigrolineata* bark resin extract 150 mg/kg, *p.o*.), and (6) UCMS + GN (*G. nigrolineata* bark resin extract 450 mg/kg, *p.o*.). Vehicle and drugs were daily administered at week 4 to week 7 at 8.00 am and 1 h before behavioral testing. One day after finishing behavioral testing, the hippocampus and frontal cortex were immediately collected and kept at −80 °C throughout the experiment. Figure 7 presents a schematic diagram of animal experimental design. According to ARRIVE guidelines 2.0, all animal exclusions were summarized in the report as a flowchart describing attrition in each group. For detailed description, see Figure S51.

### 4.7. Behavioral Study

#### 4.7.1. Y-Maze Test

Y-maze test was used to evaluate spontaneous alternation related to a sudden recall, a performance that was previously described by Kraeuter et al. [30] with some modification. The apparatus consists of three arms of equal size (3 cm × 39 cm × 12 cm) and is oriented at a 60 degree angle from each tail. Mice were allowed to explore the maze for 4 min, during which their number and sequence of arms visits and spontaneous alternations were recorded. Alternation is defined as successive entries into three different arms, for example 123, 321, and 213, but not 323. The measure of the animal recalling a memory is the percentage alternation, which was calculated by the following equation:

% Alternation = [(Number of alternations)/(Total arm entries − 2)] × 100

In addition, the Y-maze test was also performed for a locomotor activity to prevent false positives from behavioral task testing. It was defined by the total number of entries in each arm of the Y-maze apparatus [24].

#### 4.7.2. Novel Object Recognition Test (NORT)

The NORT is used to investigate the spatial recognition memory associated with the ability to discriminate between two sets of objects that differ in texture, color, and shape [49]. A black plastic box (52 cm length × 52 cm width × 41 cm height) was used to develop the apparatus. The NORT was divided into three sessions: pre-training, sample phase trial, and test phase trial. In the pre-training phase, all mice were given one habituation session for 10 min in the test box to explore the arena without objects 24 h before the sample phase trial. In the sample phase trial, each mouse was placed in the box facing the center of the opposite wall and exposed for a set length of time. Two identical objects were in the corner at a specified distance from each other (15 cm from each adjacent wall) and mice were allowed to explore for 5 min. Thirty minutes later, the test phase trial was conducted. One of the two objects was replaced by a novel object. The mouse was free to explore the objects for 5 min and the total time spent exploring each object and the percentage discrimination index were recorded. High percentage discrimination index represents the potential of learning and memory. The exploration ratio was calculated according to the following equation:

% Discrimination index = [(TN − TF)/(TN + TF)] × 100

where, TN = time devoted to the novel object, TF = time devoted to the familiar object.

#### 4.7.3. Morris Water Maze Test (MWMT)

The MWMT tested spatial memory and short-term memory by evaluating escape latency and time spent in a quadrant. The water maze was a circular dark pool (65 cm diameter, and 25 cm deep), filled with a depth of 20 cm with deionized water (25 ± 1 °C). The dark pool was divided into four quadrants and the platform (4 cm width × 12 cm length × 14 cm height) was submerged 1.5 cm under the water surface in the middle of one quadrant (Q1). In the platform trial test, the entry points were systematically varied among four quadrants of the pool. The mice were trained to learn to find the hidden platform until they reached a steady state of escape latency. The mouse that failed to locate the platform within 60 s was gently guided to the platform and allowed to re-orient to the distal visual cues for 20 s. In each trial, the latency to reach the platform (escape latency) and the distance covered were recorded for 5 days. For spatial working memory, a probe test was obtained after 24 h of the last training session. The platform was removed from the pool and the mouse was allowed to swim freely for 1 min. After completion of the probe test, the time spent in the target quadrant (Q1) was counted as an index of reference memory [50,51].

### 4.8. Lipid Peroxidation

The lipid peroxidation was measured in the homogenates of the hippocampus and frontal cortex by using TBARs assay as previously described by Khamphukdee et al. [24]. Both brains were weighed and homogenized in 10 volumes in a phosphate buffer (5 mM, pH 7.4). The homogenized brain was mixed with trichloroacetic acid (TCA) (Sigma-Aldrich, St. Louis, MO, USA) and centrifuged at 8000× *g*, 4 °C for 10 min. The supernatant was collected and incubated with 0.8% (*w*/*v*) of 2-thiobarbituric acid (TBA) (Sigma-Aldrich, St. Louis, MO, USA) at 100 °C for 15 min. The intensity of the pink-colored complex MDA-TBA condensation indicated the extent of a lipid peroxidation, and their protein contents were quantified by the Bradford assay [52]. The absorbance of the MDA-TBA complex was measured by UV/Visible spectrophotometer (Perkin Elmer Inc., Waltham, MA, USA) at 532 nm. Levels of the MDA were calculated from the MDA standard and presented as nmol MDA/mg protein. Serial concentrations of the standard were generated as a reference curve which exhibited a linear regression with the coefficient of determination (R^2^ = 0.9999) of standard calibration curve.

### 4.9. Inhibition of Aβ-Self Aggregation

The Thioflavin-T assay used to investigate the Aβ1-42 self-aggregation activity was performed as previously described [24]. Aβ1-42 was dissolved in a 50 mM phosphate buffer (pH 7.4) to obtain a 250 mM stock solution. Compounds **1**–**6**, **8**–**10,** and curcumin (a positive control) were first prepared at a concentration of 10 mM in dimethyl sulfoxide (DMSO). In black and opaque 96-well plates, 2 µL of each compound were added at a concentration of 100 µM. Then, in each well, Aβ1-42 solution was added to give a final concentration of 10 µM and mixed thoroughly. Covered plates were left in the dark for 24 h at 37 °C without agitation. After incubation, each well was loaded with 190 µL of 5 M ThT in 50 mM glycine/NaOH buffer (pH 8.0), and fluorescence intensities were measured at 446 nm and 490 nm for excitation and emission, respectively. The percent inhibition of each compound was calculated and reported.

### 4.10. Inhibitory Activity of Recombinant Human Monoamine Oxidase Enzymes

The inhibitory activity assays of the isolated compound with recombinant human MAO-A and MAO-B (Sigma-Aldrich, St. Louis, MO, USA) were carried out with some modification as described recently by [53]. In brief, **1**–**6** and **8**–**10** were first dissolved in DMSO to obtain a stock solution with a concentration of 25 mM. The activity was determined by serial dilution of each concentration of the compounds. For the MAO-A inhibitor assay, 4.5 µL of kynuramine (Sigma-Aldrich, St. Louis, MO, USA) were mixed with 234.75 µL of potassium phosphate buffer (100 mM, pH 7.4, made isotonic with KCl, 20.2 mM). Then, 3 µL of kynuramine were mixed with 236.25 µL of potassium phosphate buffer (pH 7.4) for the MAO-B inhibitor assay. The reaction was stopped by adding 400 µL of 2N NaOH and 1000 µL of water. The fluorescence of 4-hydroxyquinoline (Sigma-Aldrich, St. Louis, MO, USA), a by-product of this reaction, was measured with the excitation wavelength at 310 nm and the emission wavelength at 400 nm. The initial rates of MAO-catalyzed kynuramine oxidation were plotted *versus* the logarithm of the inhibitor concentrations and reported as IC_50_ values by using the Prism software package (GraphPad Software, version 5, San Diego, CA, USA) to evaluate quantitative. IC_50_ values were converted to *Ki* values by using the equation *Ki* = IC_50_/(1 + [S]/*Km*). Clorgyline (Sigma-Aldrich, St. Louis, MO, USA) and deprenyl (Sigma-Aldrich, St. Louis, MO, USA) were used as referent standards for selective MAO-A and MAO-B inhibitors, respectively.

### 4.11. Statistical Analysis

The results obtained from animal behavioral tests and neurochemical study were expressed as the mean ± standard error of the mean (SEM) and were analyzed by one-way analysis of variance (ANOVA), followed by the Tukey test for multiple comparisons among different groups for Y-maze test, NORT, and Probe test of MWMT. The one-way repeated measures ANOVA were used for training test of MWMT. The difference with *p* values < 0.05 was considered as statistically significant. The software SigmaStat^®^ ver. 3.5 (SYSTAT Software Inc., Richmond, CA, USA) was used for data analysis. Additionally, the data from the in vitro tests were expressed as mean ± standard deviation (SD).

## 5. Conclusions

Treatment of the unpredictable chronic mild stress mice with *G. nigrolineata* bark resin extract ameliorates their memory deficits. One of the possible mechanisms is through attenuation of oxidative stress in both frontal cortex and hippocampus via lipid peroxidation. The brain protective effects of *G. nigrolineata* bark resin extract was supported by the inhibition of MAO-A and MAO-B activities by its prenylated xanthone constituents (**1**–**6** and **8**–**10**) as well as the inhibition of the Aβ1-42 self-aggregation by **1** and **2**. The findings of this study suggest that *G. nigrolineata* bark resin extract and its prenylated xanthone constituents offer potential neuroprotection against stress-induced cognitive dysfunction.

## Figures and Tables

**Table 1:** Inhibitory effects of **1**–**6** and **8**–**10** on Aβ self-aggregation and MAOs.

Compounds	% Inhibition of Aβ Aggregation	IC_50_ (µM)		Si	
		MAO-A	MAO-B	MAO-A	MAO-B
**1**	46.79 ± 5.46	100.2 ± 0.35	17.99 ± 0.11	3.407	0.294
**2**	58.56 ± 3.28	119.8 ± 0.75	238.50 ± 2.03	0.307	3.254
**3**	Not detected	79.81 ± 0.50	40.09 ± 0.89	1.218	0.821
**4**	Not detected	40.21 ± 0.35	15.60 ± 0.26	1.579	0.6333
**5**	Not detected	7.10 ± 0.03	16.34 ± 1.44	0.266	3.762
**6**	Not detected	69.92 ± 0.06	0.07 ± 0.01	558.273	0.00179
**8**	Not detected.	100.5 ± 0.44	>400	<0.153	0.6522
**9**	Not detected	25.41 ± 0.63	0.40 ± 0.01	38.705	0.0256
**10**	Not detected	3.58 ± 0.02	>400	<0.005	183.168
Curcumin ^1^	51.73 ± 2.89	-	-	-	-
Clogyline ^2^	-	0.005 ± 0.00	0.015 ± 0.002	0.143	7
Deprenyl ^3^	-	8.48 ± 0.14	0.10 ± 0.02	48.565	0.021

^1^ Curcumin was used as a positive control for Aβ self-aggregation; ^2^ Clorgyline was used as a reference standard for selective MAO-A inhibitor and ^3^ Deprenyl was used as a reference standard for selective MAO-B inhibitor.

## Data Availability

Data sharing is not applicable to this article.

## Supplementary Materials

The following supporting information can be downloaded at: www.mdpi.com/article/10.3390/molecules27093014/s1, Figures S1–S50: ^1^H, ^13^C, COSY, HSQC and HMBC spectra of **1**–**10**; Figure S51: A flow chart of the experimental protocol with the number of animals used, died, and included in this study; Table S1–S10: ^1^H and ^13^C NMR and HMBC assignments of **1–10**; Table S11–12: One-way analysis of variance (ANOVA) test of Y-maze test and novel object recognition test (NORT); Table S13.1: One-Way Repeated Measures ANOVA test of MWMT on the training test, Table S13.2: One-way analysis of variance (ANOVA) test of MWMT on the probe test; Table S14.1: One-way analysis of variance (ANOVA) test of UCMS-Induced lipid peroxidation in frontal cortex; Table S14.2: **2** One-way analysis of variance (ANOVA) test of UCMS-Induced lipid peroxidation in hippocampus.

## Sample Availability

Samples of the compounds are not available from the authors.
