# Supplementary material for: Effects of the Bark Resin Extract of Garcinia nigrolineata on Chronic Stress-Induced Memory Deficit in Mice Model and the In Vitro Monoamine Oxidases and β-Amyloid Aggregation Inhibitory Activities of Its Prenylated Xanthone Constituents
Source: Molecules. 2022 May 7;27(9):3014. doi: 10.3390/molecules27093014 (PMC9103351; doi:10.3390/molecules27093014)
Supplement: Supplementary file 1 [file molecules-27-03014-s001.zip › molecules-1702294-supplementary.pdf]

Supplementary Materials

Effects of the Bark Resin Extract of *Garcinia nigrolineata* on Chronic Stress-Induced Memory Deficit in Mice Model and the *In Vitro* Monoamine Oxidases and β-Amyloid Aggregation Inhibitory Activities of Its Prenylated Xanthone Constituents

Charinya Khamphukdee[1], Ibrahim Turkmani[2], Yutthana Chotritthirong[3], Yaowared Chulikhit[4], Chantana Boonyarat[4], Nazim Sekeroglu[5], Artur M. S. Silva[6], Orawan Monthakantirat[4]*, and Anake Kijjoa[2]*

[1] Division of Pharmacognosy and Toxicology, Faculty of Pharmaceutical Sciences, Khon Kaen University, Khon Kaen 40002, Thailand. E-mail: charkh@kku.ac.th (C.K.)

[2] ICBAS-Instituo de Ciências Biomédicas Abel Salazar and CIIMAR, Universidade do Porto, Rua de Jorge Viterbo Ferreira 228, 4050-313 Porto, Portugal. E-mail: ibrahimturkmani94@gmail.com (I.T.).

[3] Graduate School of Pharmaceutical Sciences, Khon Kaen University, Khon Kaen 40002, Thailand. E-mail: yutthana_ch@kkumail.com (Y.Ch.)

[4] Division of Pharmaceutical Chemistry, Faculty of Pharmaceutical Sciences, Khon Kaen University, Khon Kaen 40002, Thailand. E-mail: yaosum@kku.ac.th (Y.C.); chaboo@kku.ac.th (C.B.); oramon@kku.ac.th (O.M.)

[5] Phytotherapy, Medicinal and Aromatic Plants Application & Research Center and Biology Department, Faculty of Arts and Science, Gaziantep University, 27310-Gaziantep, Turkey. E-mail: nsekeroglu@gmail.com (N.S.)

[6] Departamento de Química & QOPNA, Universidade de Aveiro, 3810-193 Aveiro, Portugal. E-mail: artur.silva@ua.pt (A.M.S. S.)

*Correspondence: oramon@kku.ac.th (O.M.); Tel.: +66-81-3404677; ankijjoa@icbas.up.pt (A.K.); Tel.: +351-220428331

Figure S1. ^{1}H NMR spectrum of cowagarcinone C (DMSO-*d6*, 300 MHz).

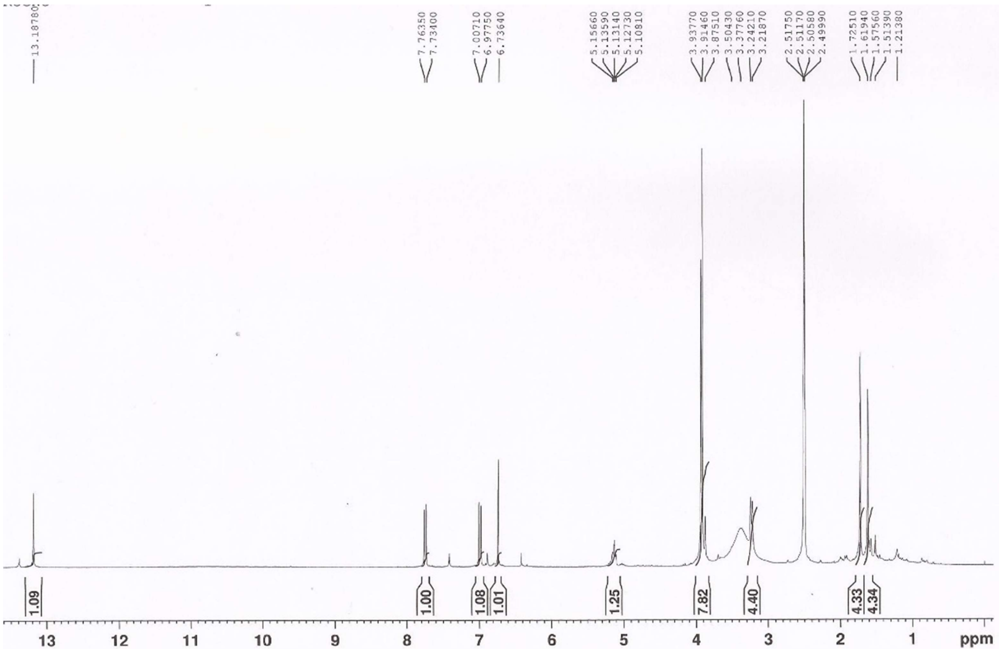

Figure S2. ^{13}C NMR spectrum of cowagarcinone C (DMSO-*d6*, 75MHz).

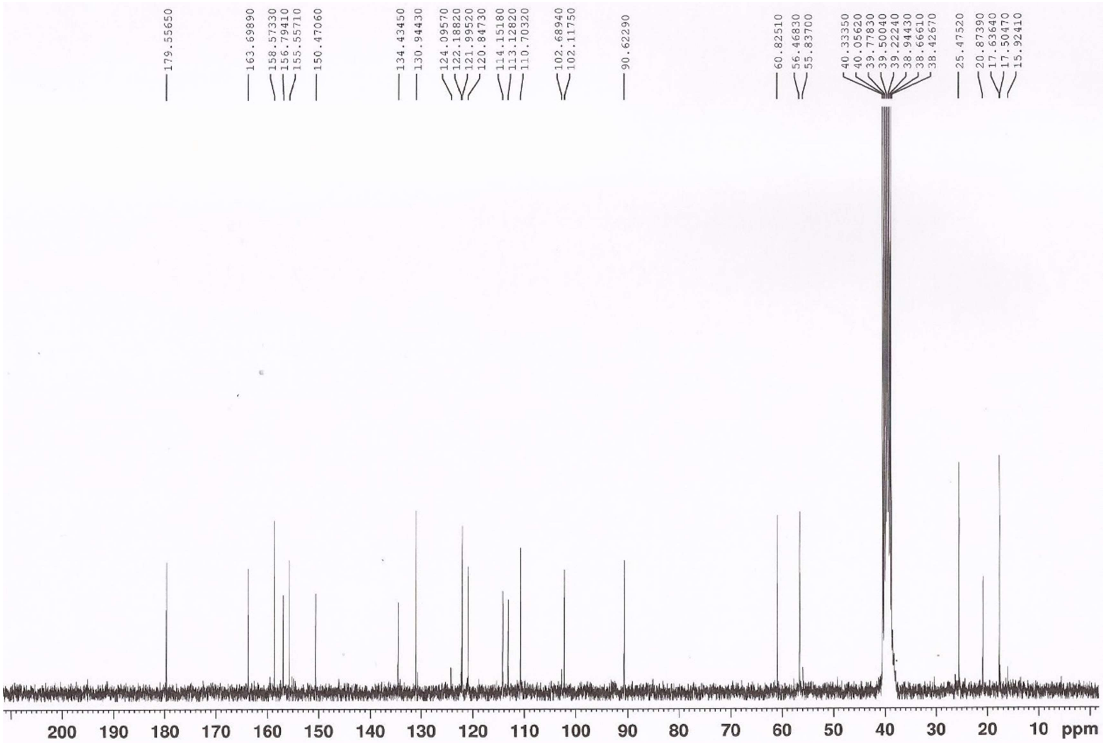

Figure S3. COSY spectrum of cowagarcinone C (DMSO-*d6*, 300 MHz).

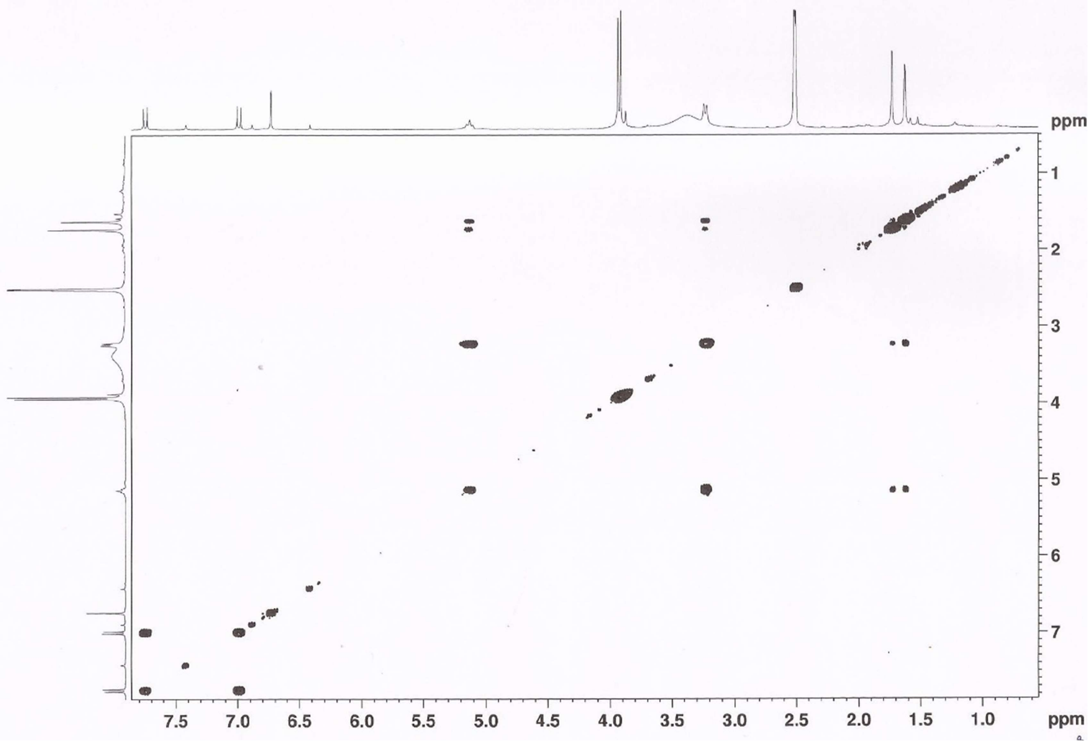

Figure S4. HSQC spectrum of cowagarcinone C (DMSO-*d6*, 300 MHz).

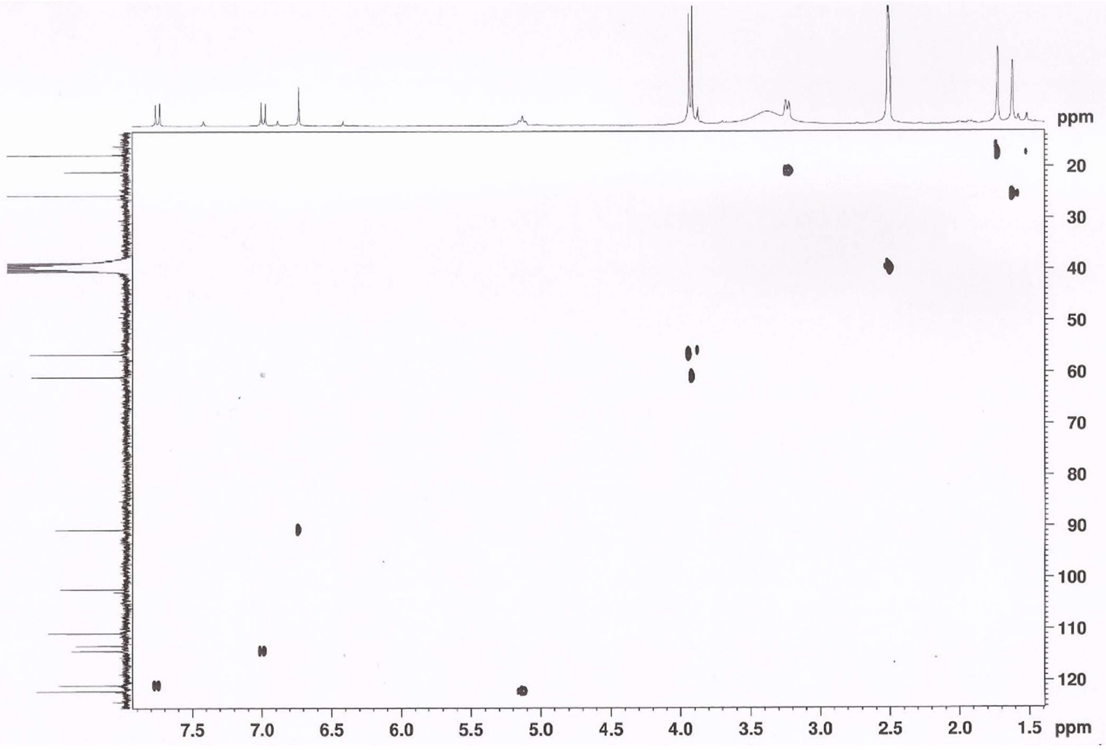

Figure S5. HMBC spectrum of cowagarcinone C (DMSO-*d6*, 300 MHz).

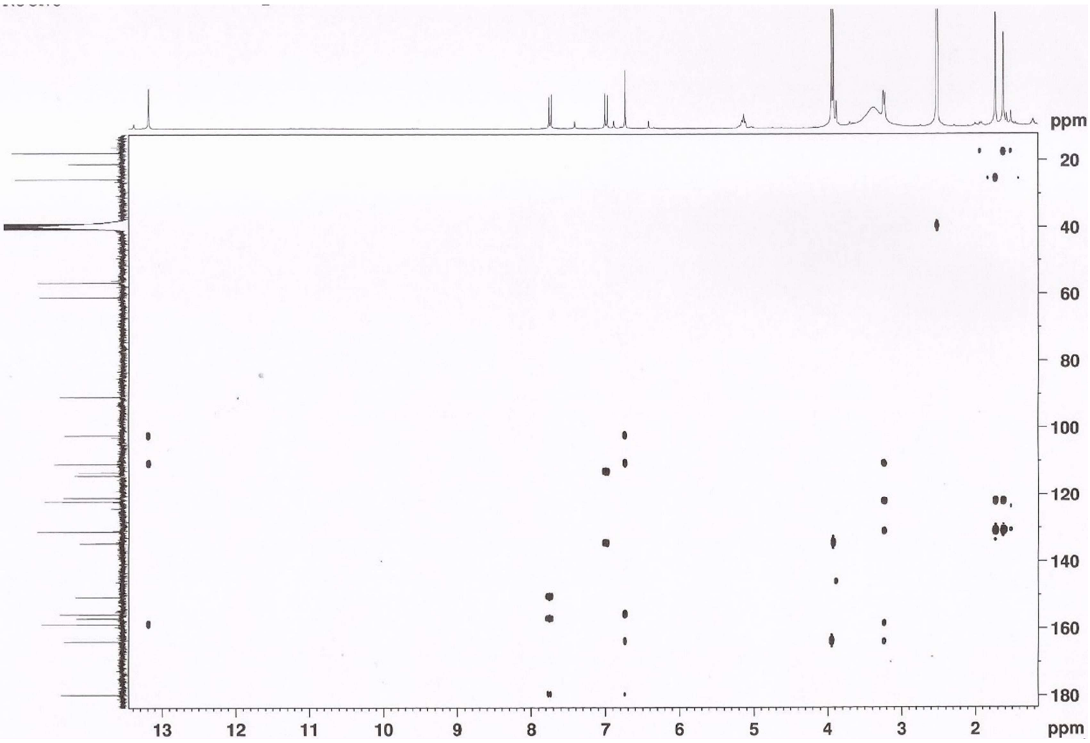

Figure S6. ^{1}HNMR spectrum of cowaxanthone (DMSO-*d6*, 300 MHz).

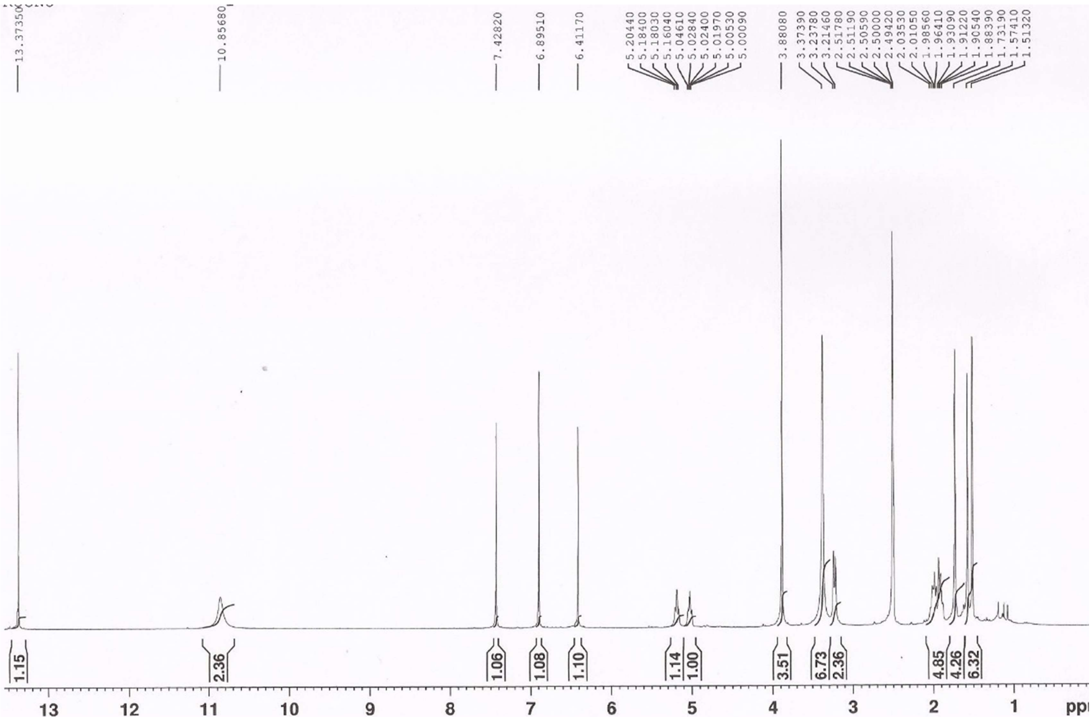

Figure S7. ^{13}C NMR spectrum of cowaxanthone (DMSO-*d6*, 75 MHz).

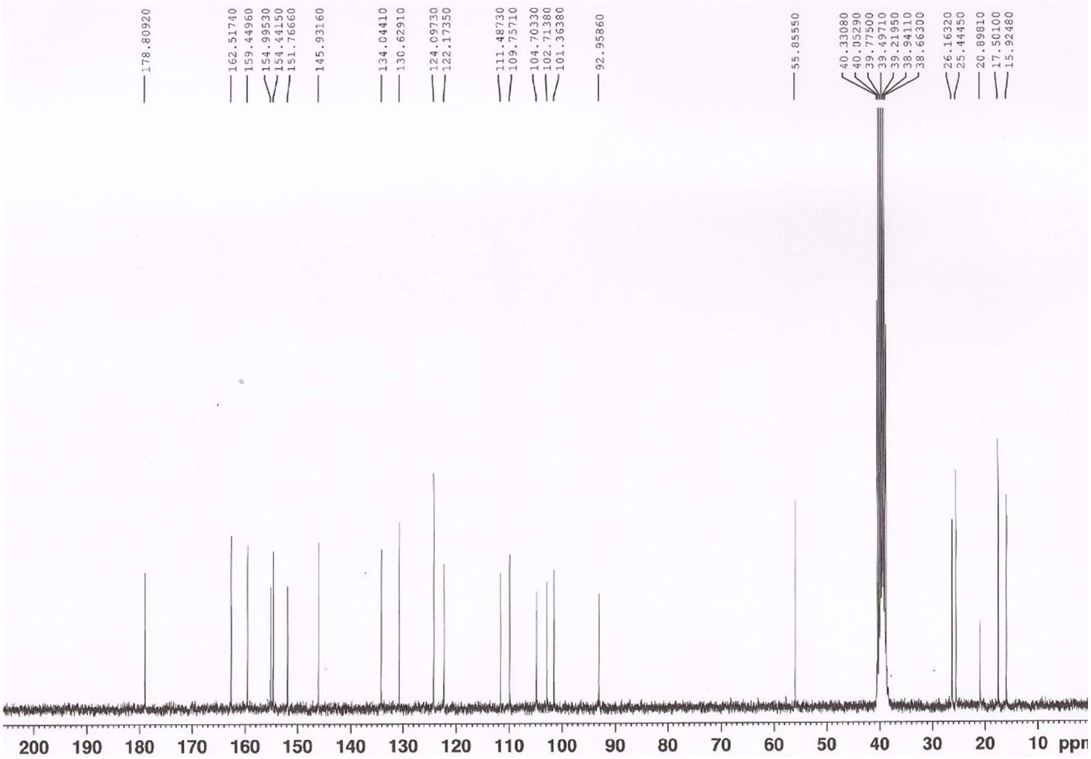

Figure S8. COSY spectrum of cowaxanthone (DMSO-*d6*, 300 MHz).

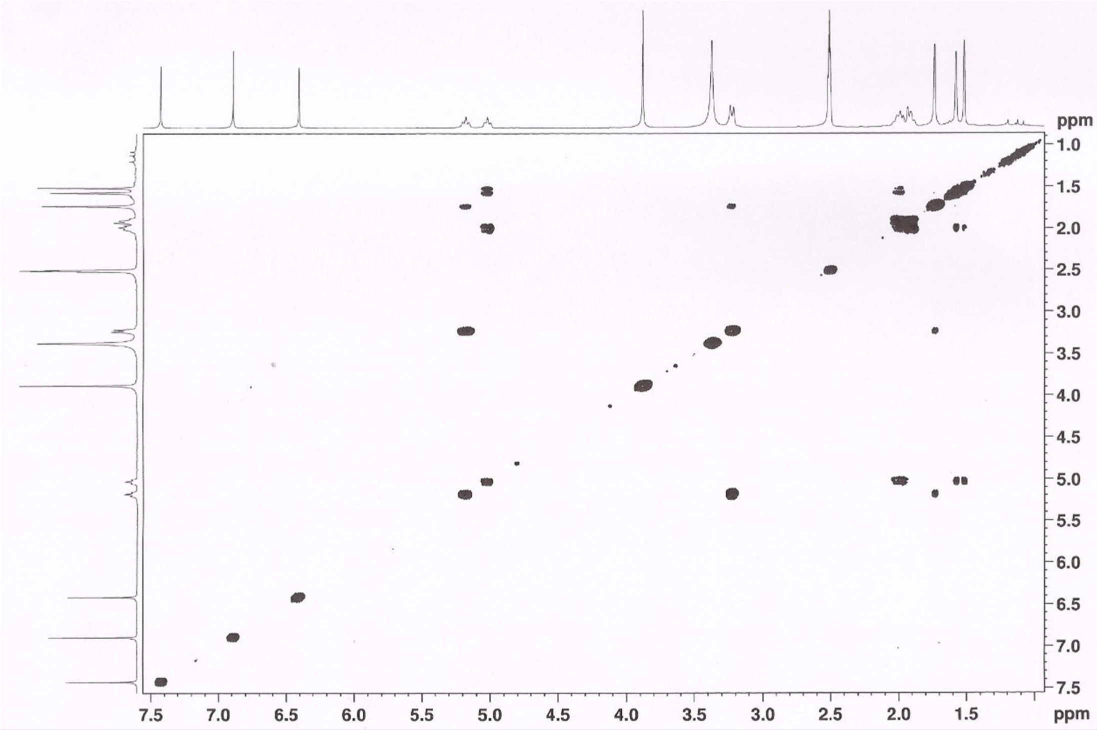

Figure S9. HSQC spectrum of cowaxanthone (DMSO-*d6*, 300 MHz).

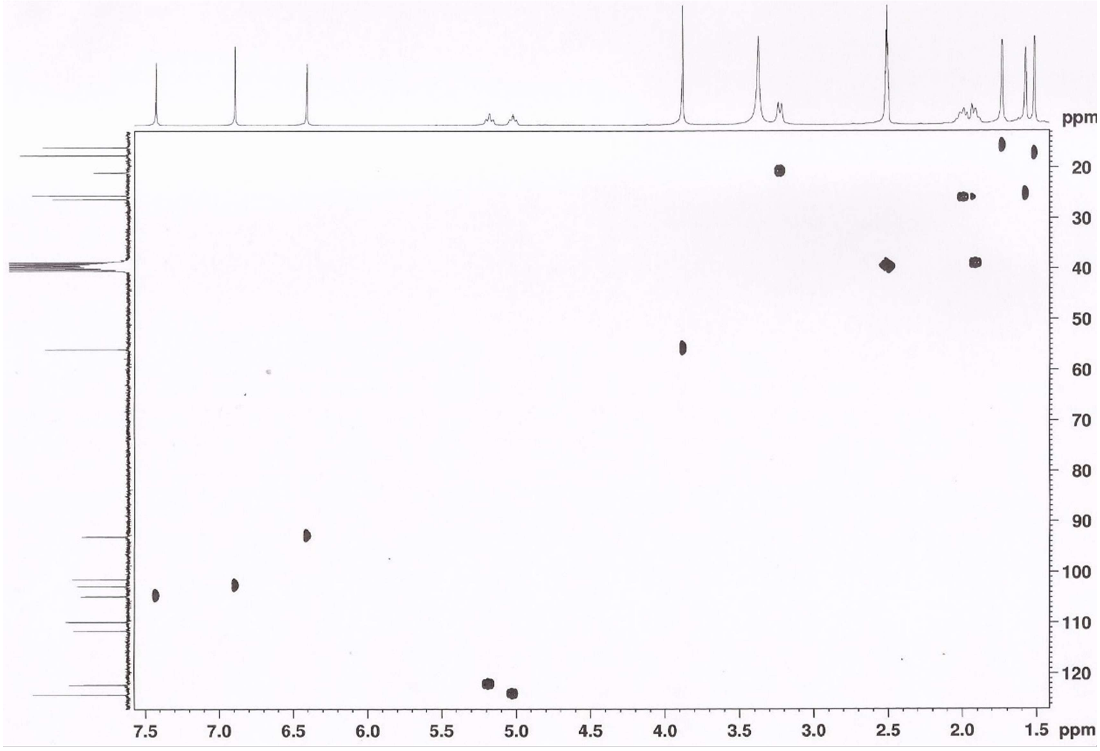

Figure S10. HMBC spectrum of cowaxanthone (DMSO-*d6*, 300 MHz).

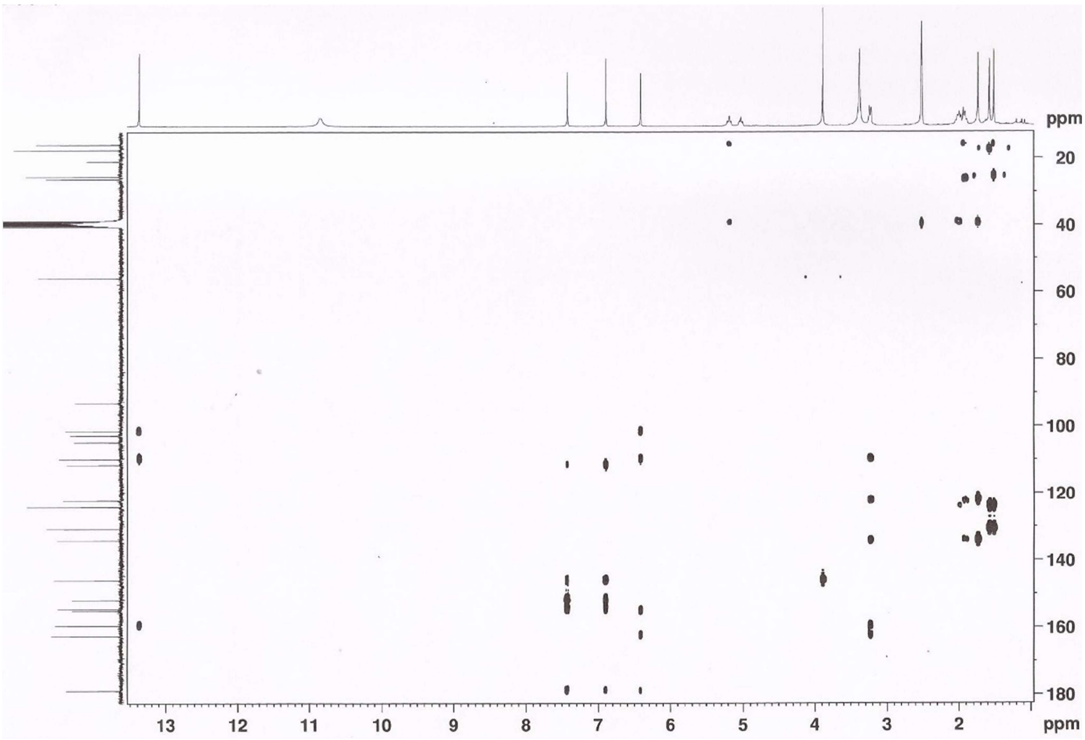

Figure S11. ^{1}H NMR spectrum of α-mangostin ($CDCl_3$, 300 MHz).

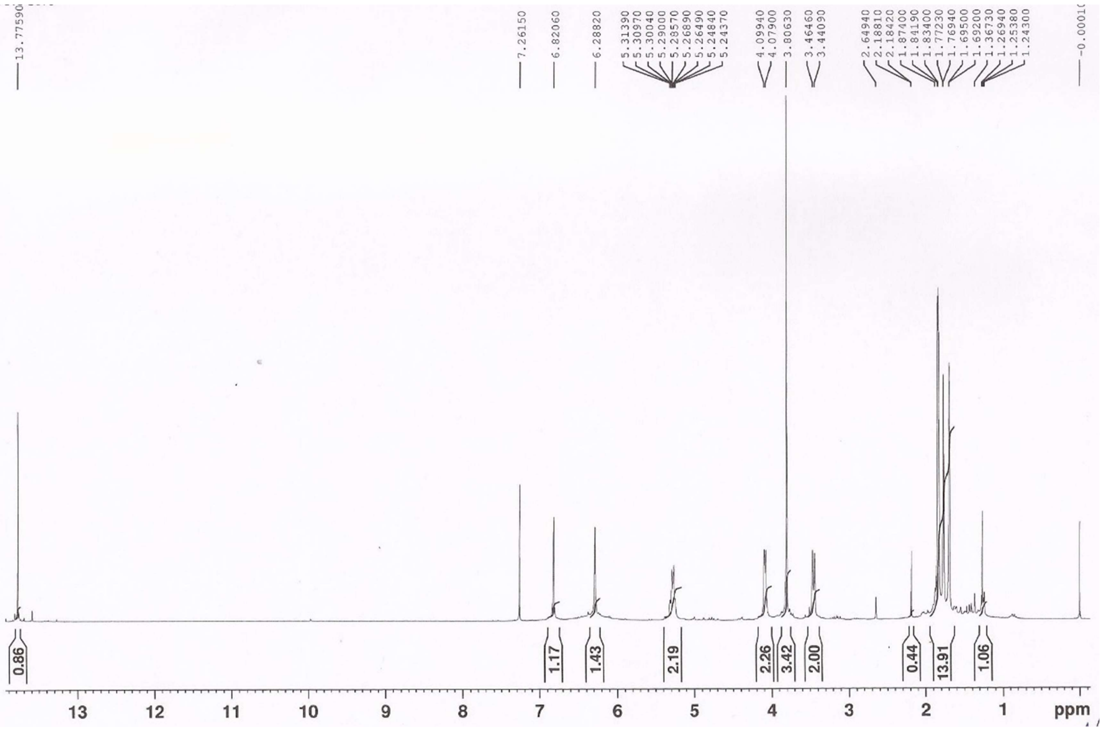

Figure S12. ^{13}C NMR spectrum of α-mangostin ($CDCl_3$, 75 MHz).

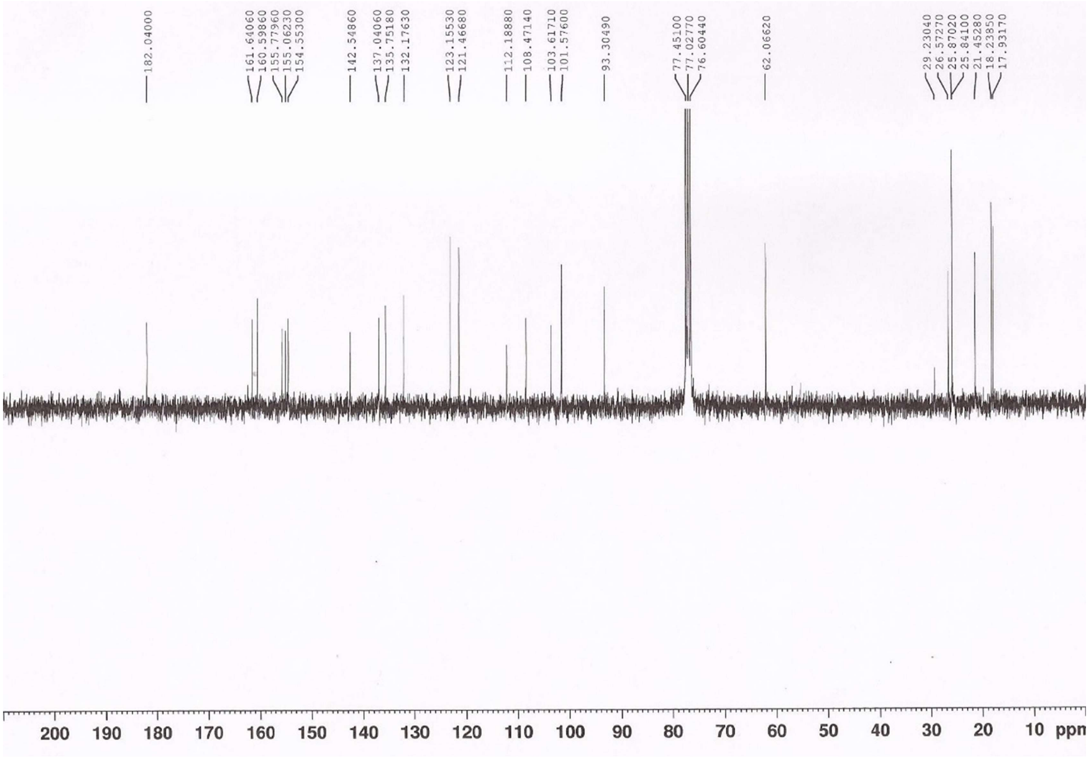

Figure S13. COSY spectrum of α-mangostin ($CDCl_3$, 300 MHz).

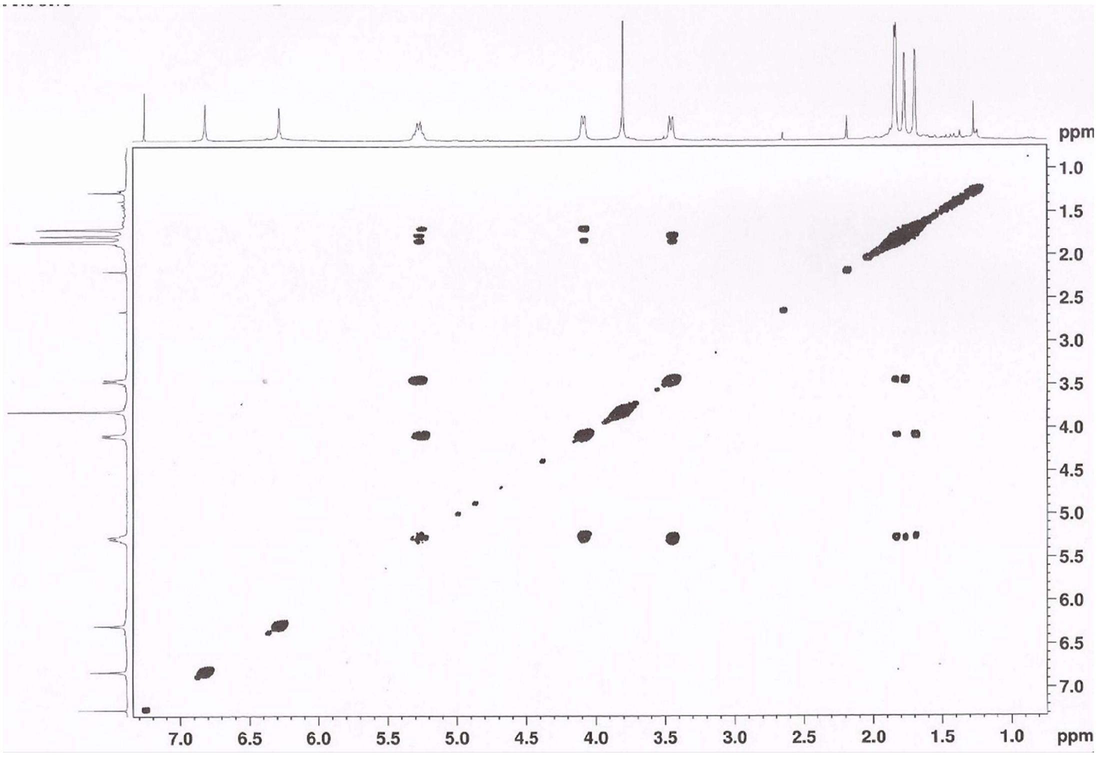

Figure S14. HSQC spectrum of α-mangostin ($CDCl_3$, 300 MHz).

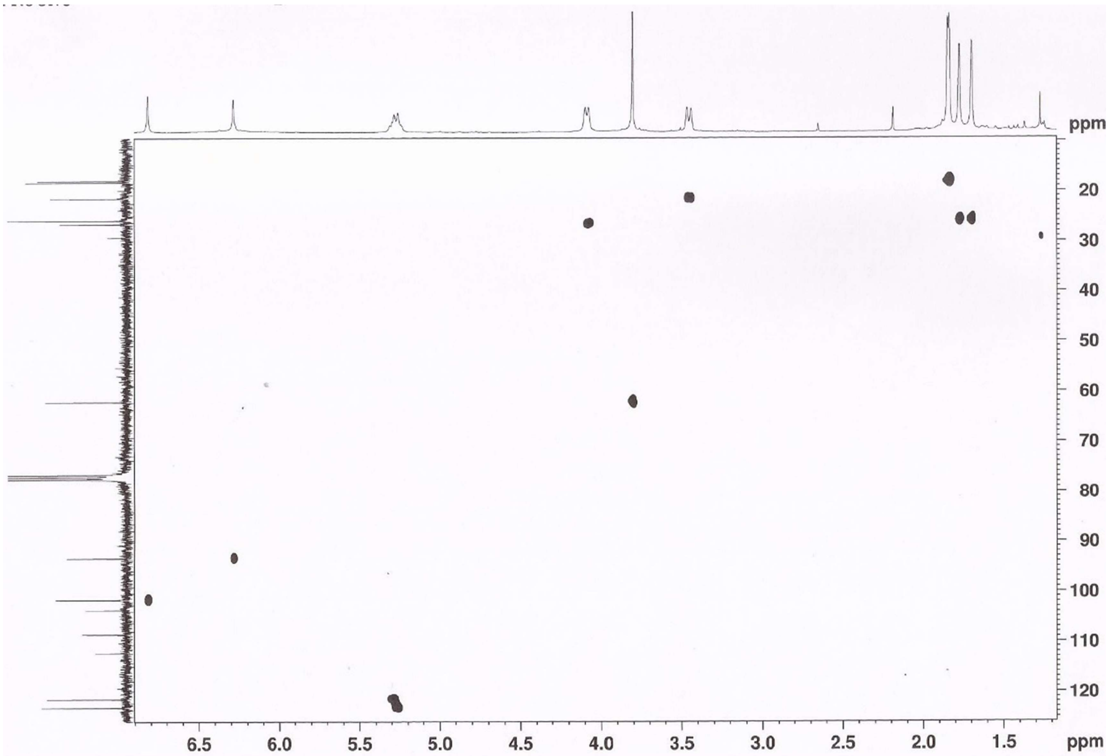

Figure S15. HMBC spectrum of α-mangostin ($CDCl_3$, 300 MHz).

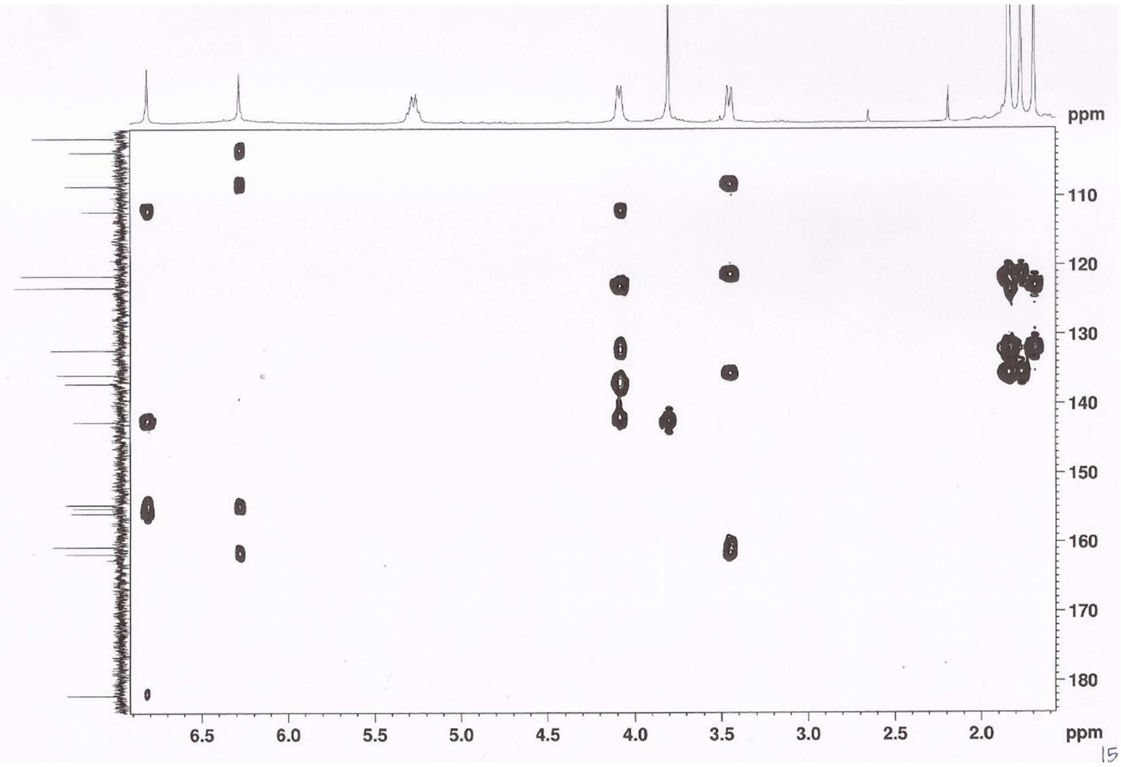

Figure S16. ^{1}H NMR spectrum of cowaxanthone B ($CDCl_3$, 300 MHz).

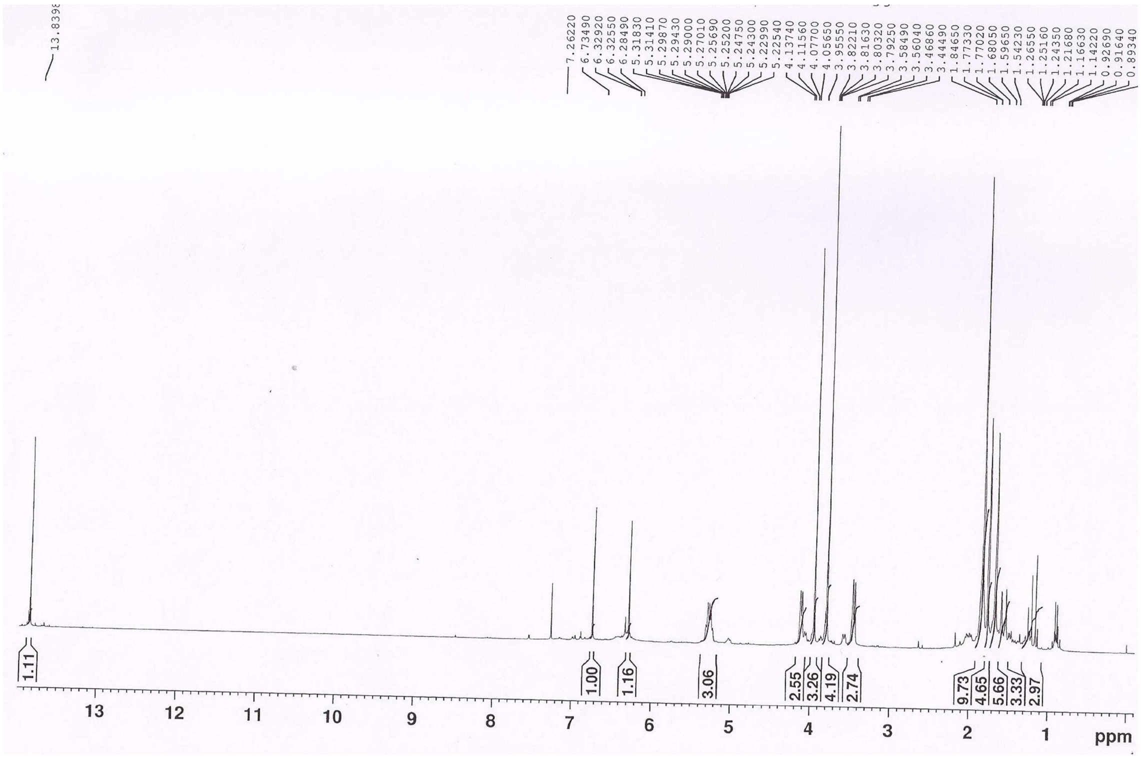

Figure S17. ^{1}C NMR spectrum of cowaxanthone B ($CDCl_3$, 75 MHz).

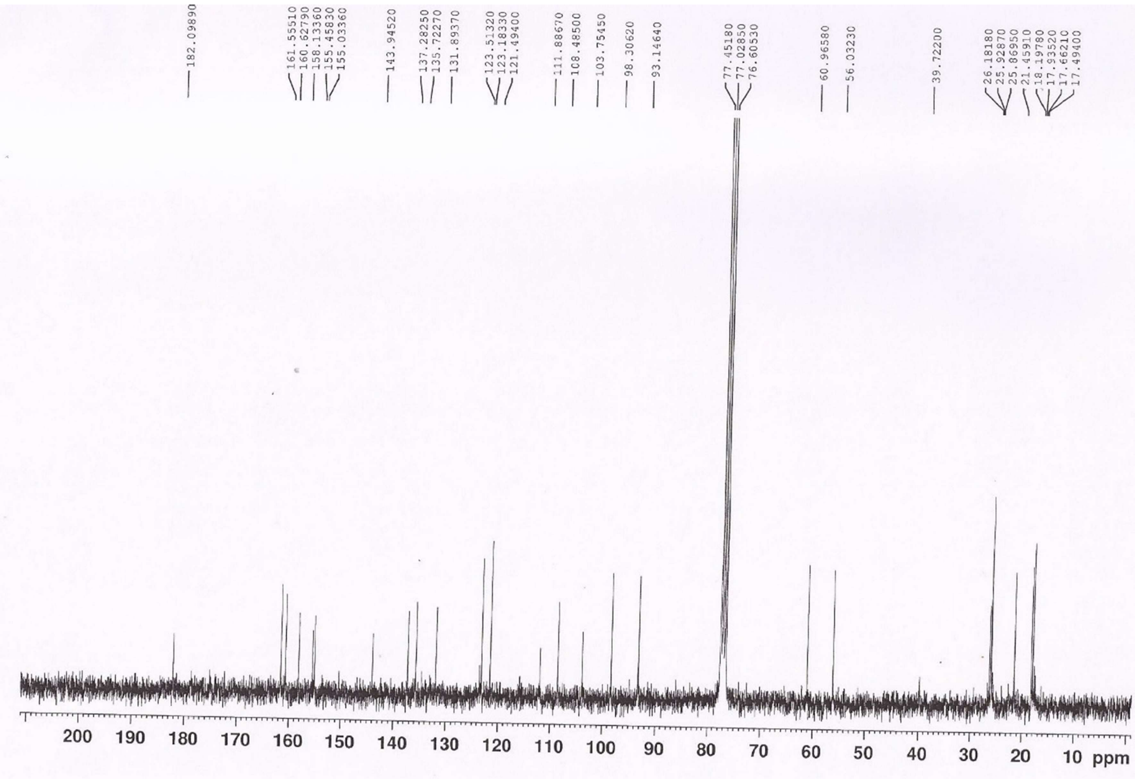

Figure S18. COSY spectrum of cowaxanthone B ($CDCl_3$, 300 MHz).

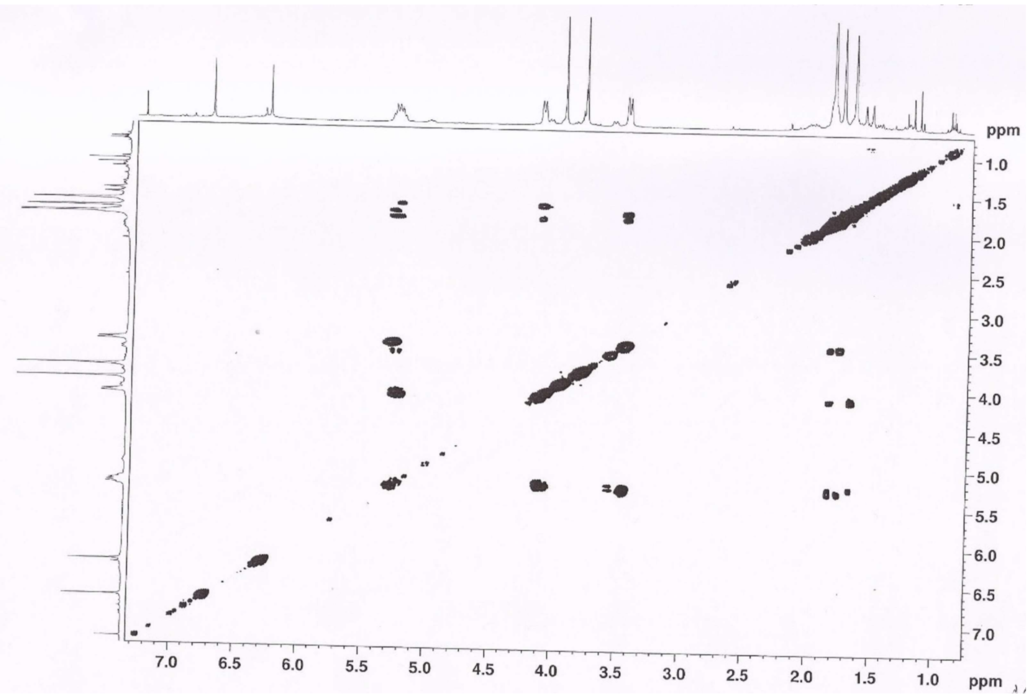

Figure S19. HSQC spectrum of cowaxanthone B ($CDCl_3$, 300 MHz).

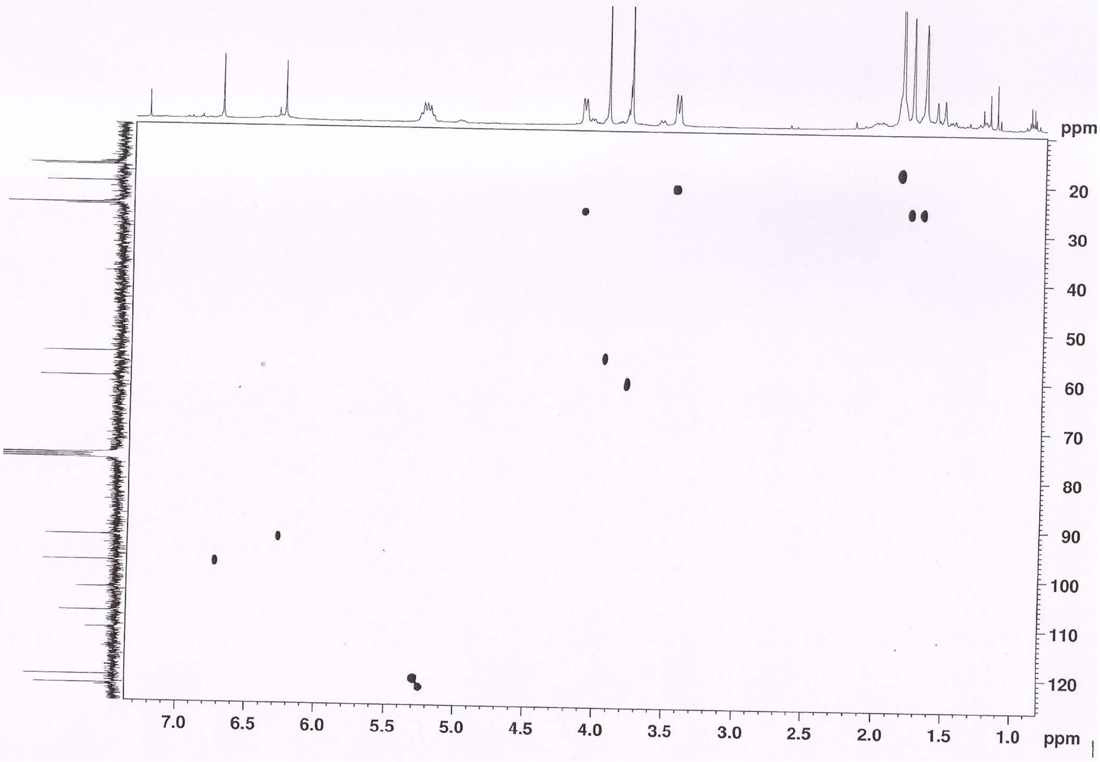

Figure S20. HSMBC spectrum of cowaxanthone B ($CDCl_3$, 300 MHz).

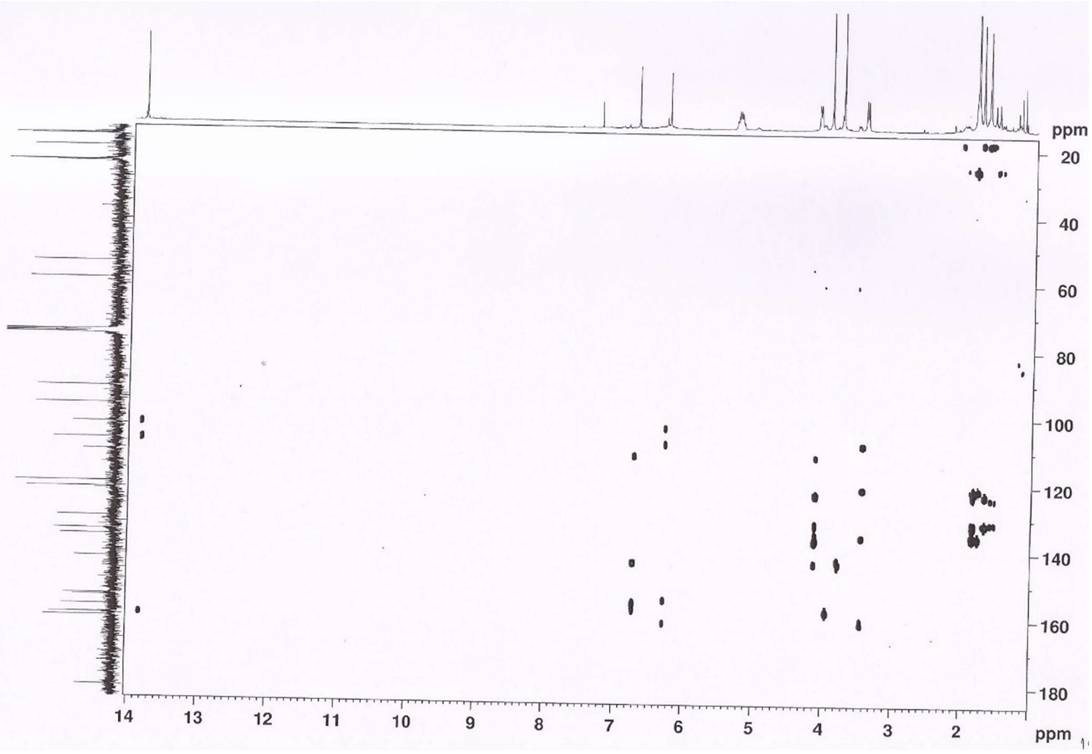

Figure S21. ^{1}HNMR spectrum of cowanin (DMSO-*d6*, 300 MHz).

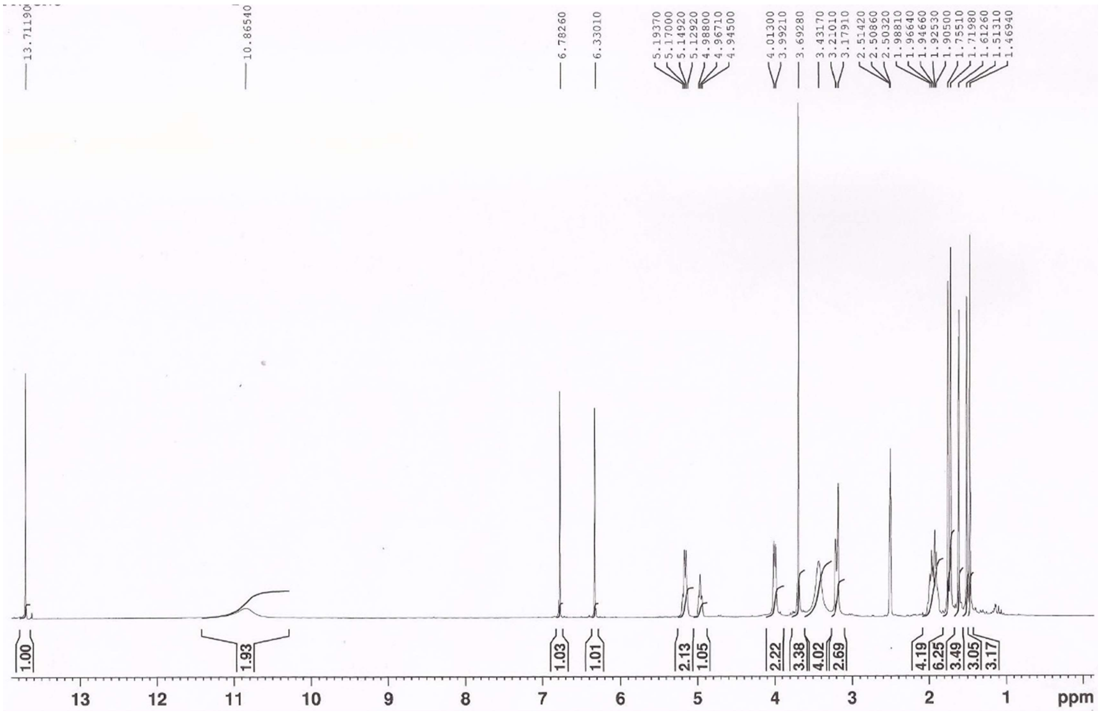

Figure S22. ^{13}C NMR spectrum of cowanin (DMSO-*d6*, 75 MHz).

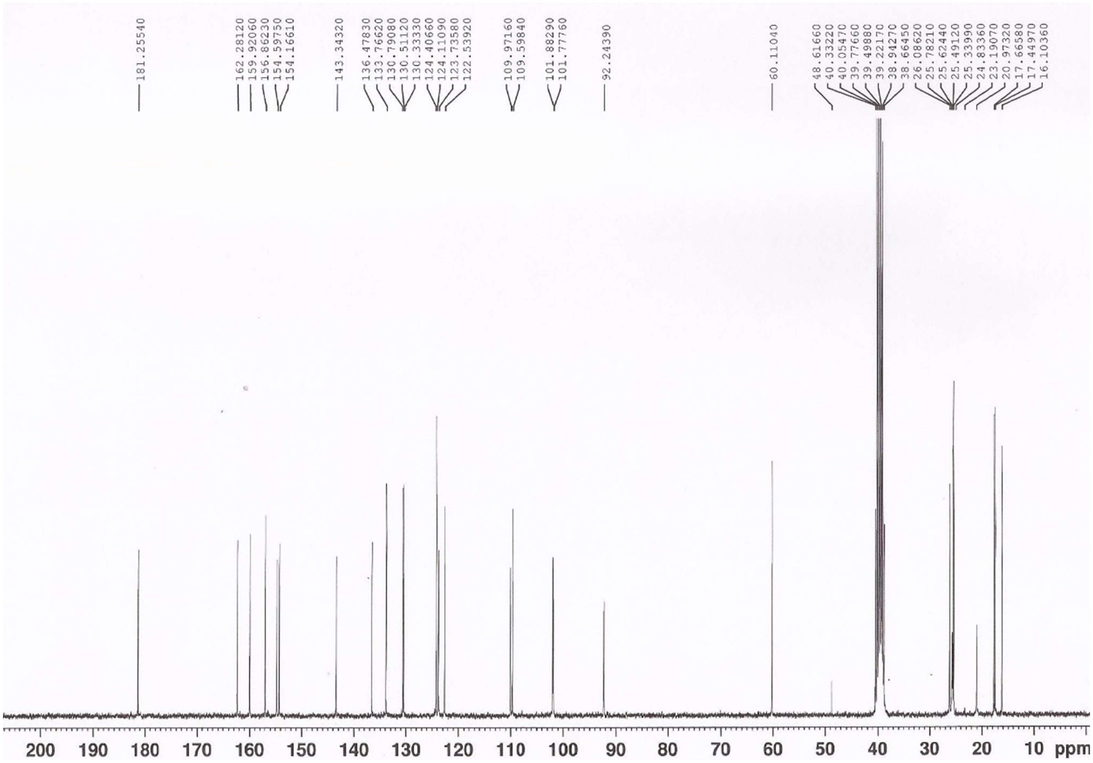

Figure S23. COSY spectrum of cowanin (DMSO-*d6*, 300 MHz).

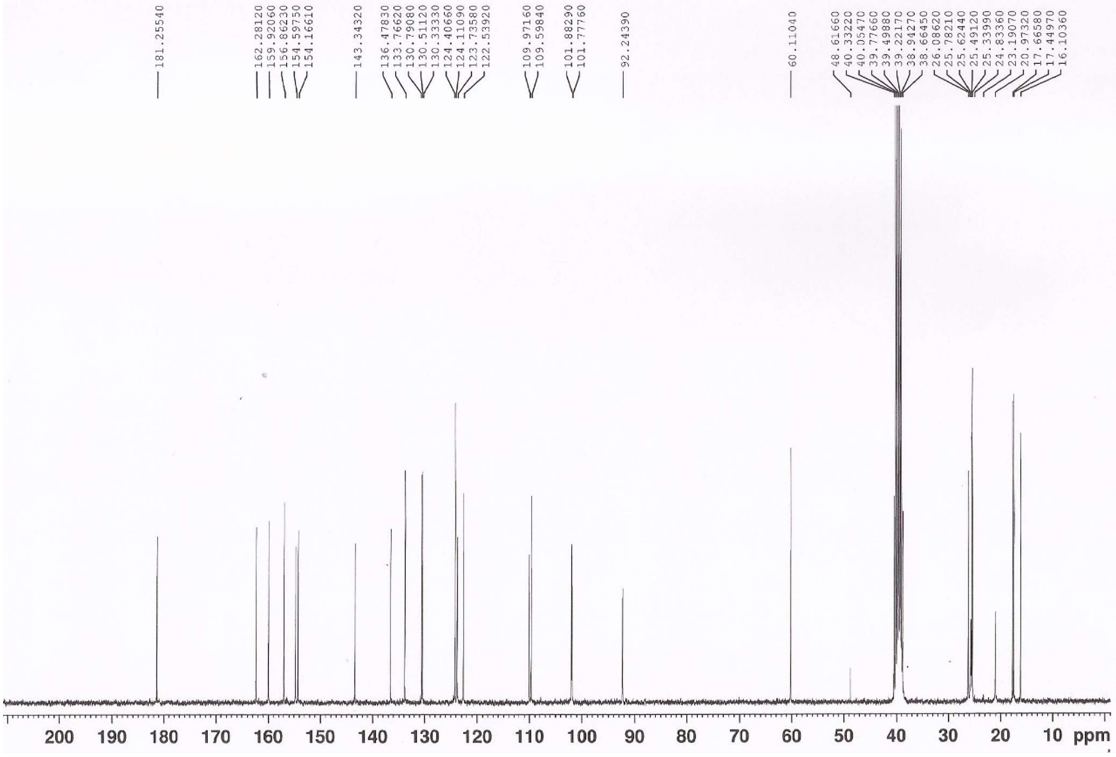

Figure S24. HSQC spectrum of cowanin (DMSO-*d6*, 300 MHz).

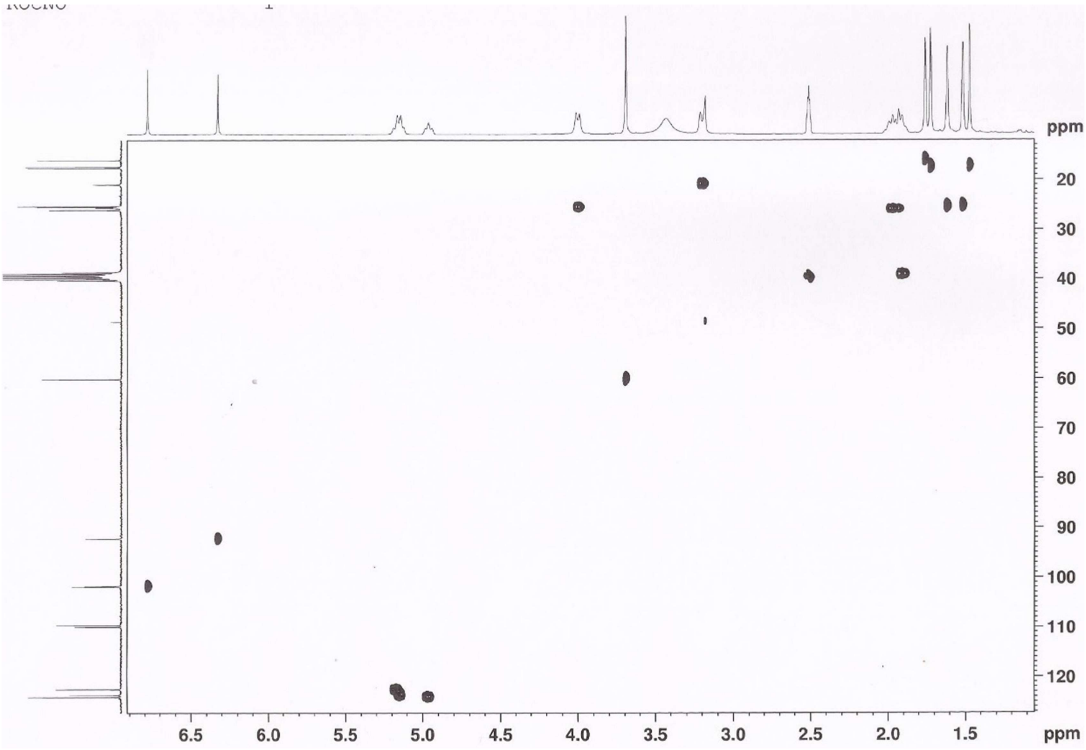

Figure S25. HMBC spectrum of cowanin (DMSO-*d6*, 300 MHz).

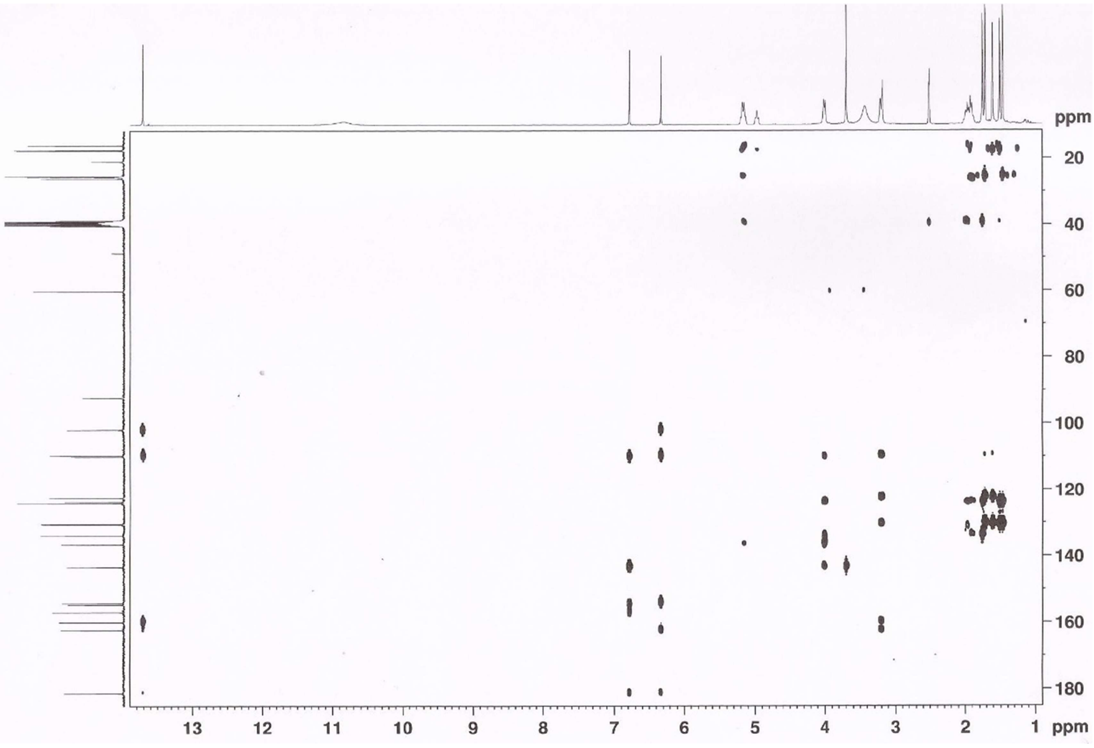

Figure S26. ^{1}H spectrum of fuscaxanthone A (DMSO-*d6*, 300 MHz).

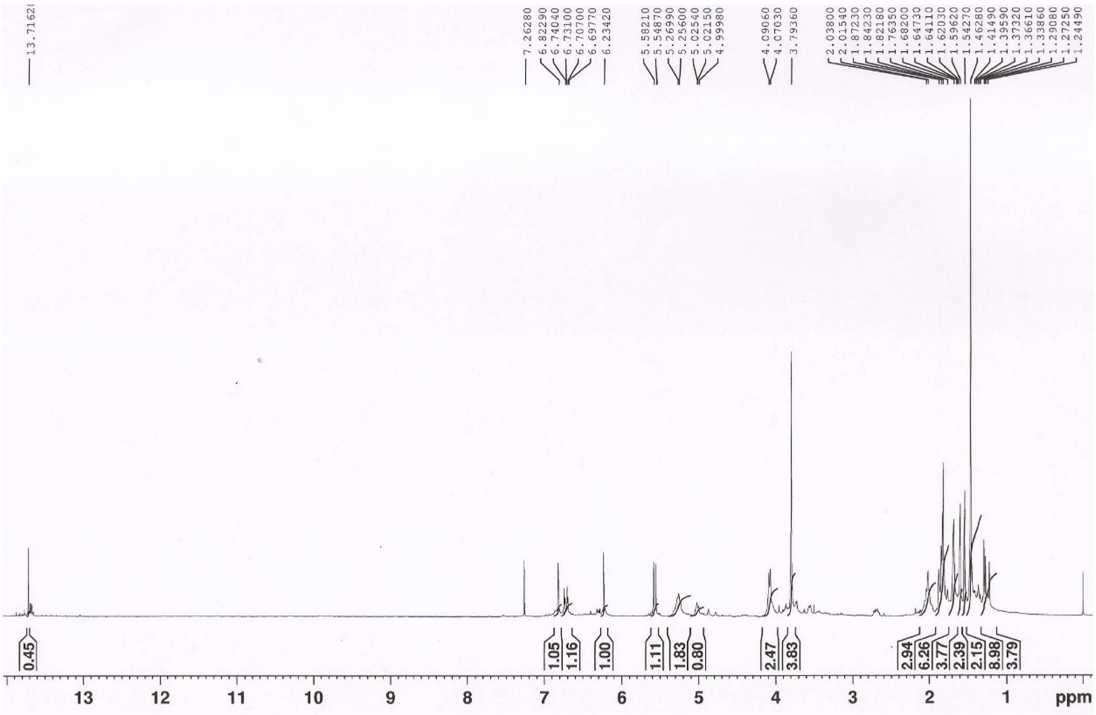

Figure S27. ^{13}C NMR spectrum of fuscaxanthone A (DMSO-*d6*, 75 MHz).

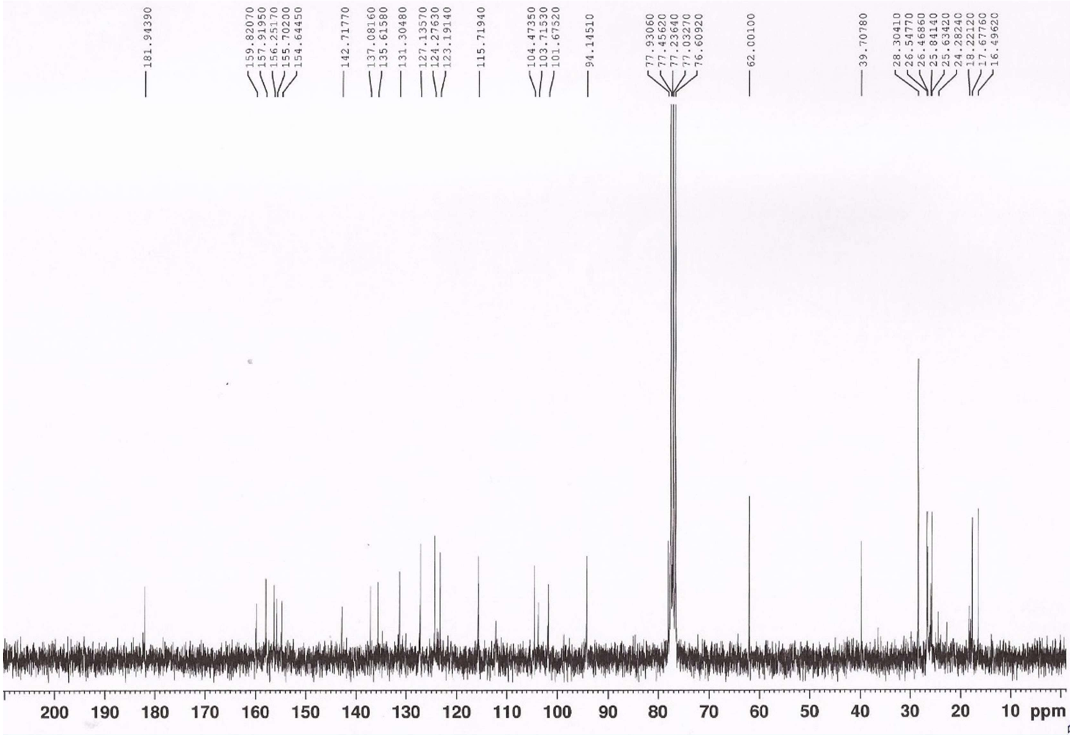

Figure S28. COSY spectrum of fuscaxanthone A (DMSO-*d6*, 300 MHz).

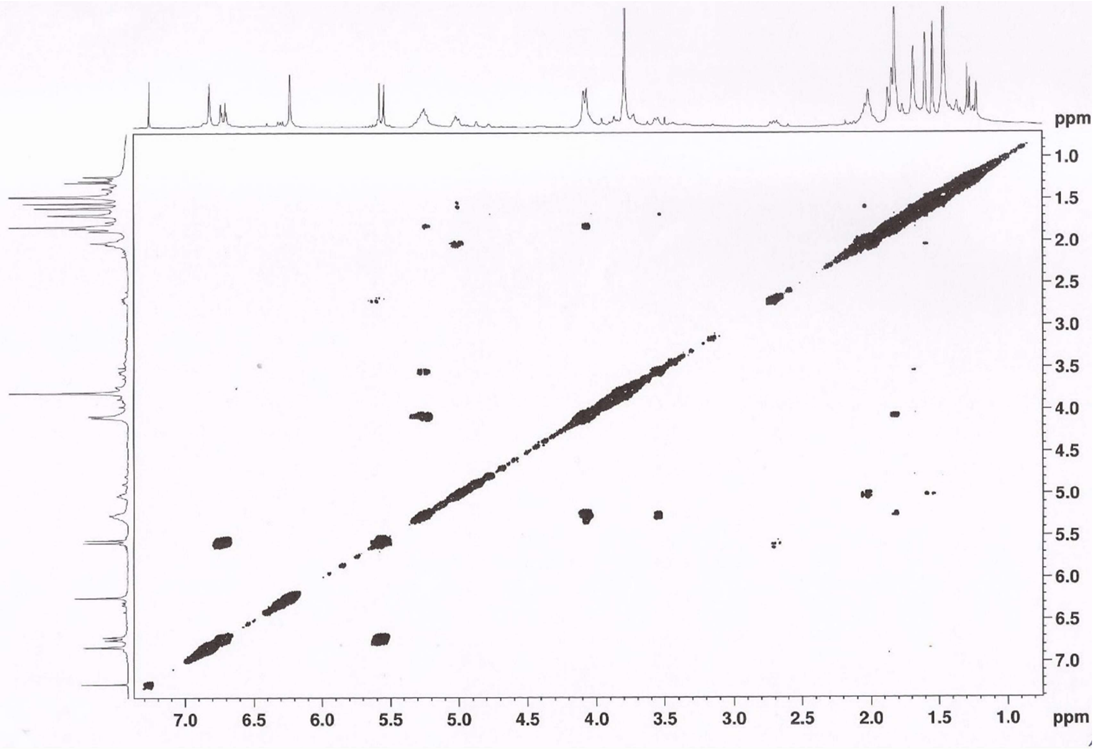

Figure S29. HSQC spectrum of fuscaxanthone A (DMSO-*d6*, 300 MHz).

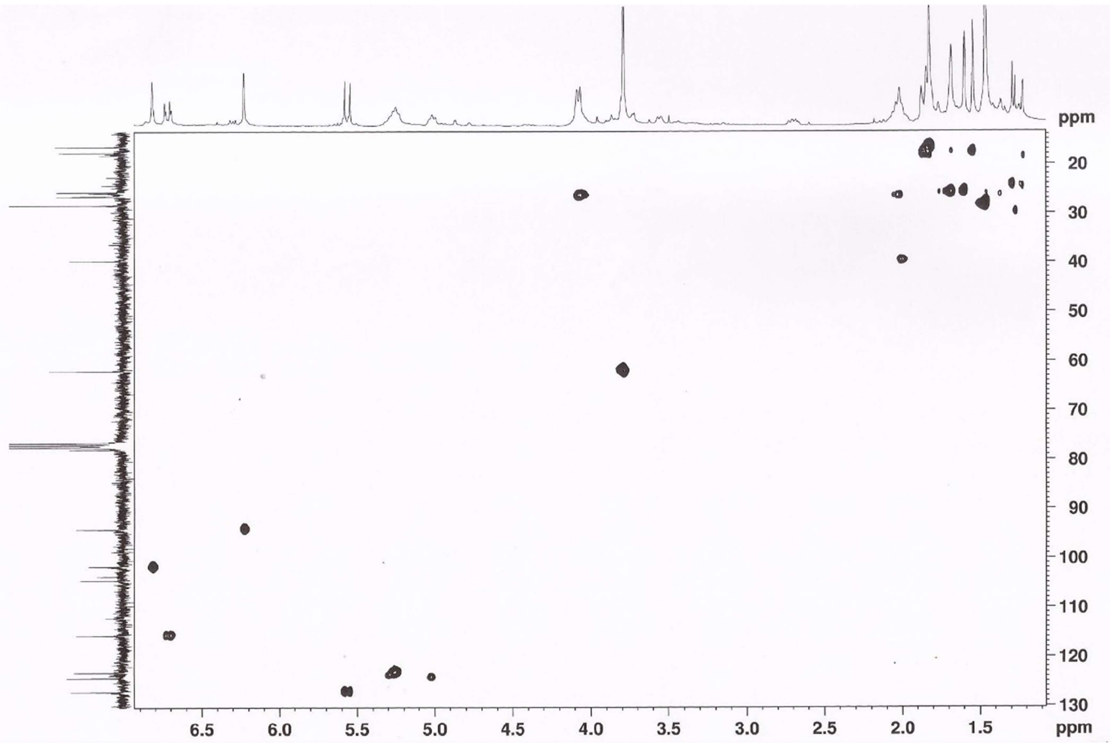

Figure S30. HMBC spectrum of fuscaxanthone A (DMSO-*d6*, 300 MHz).

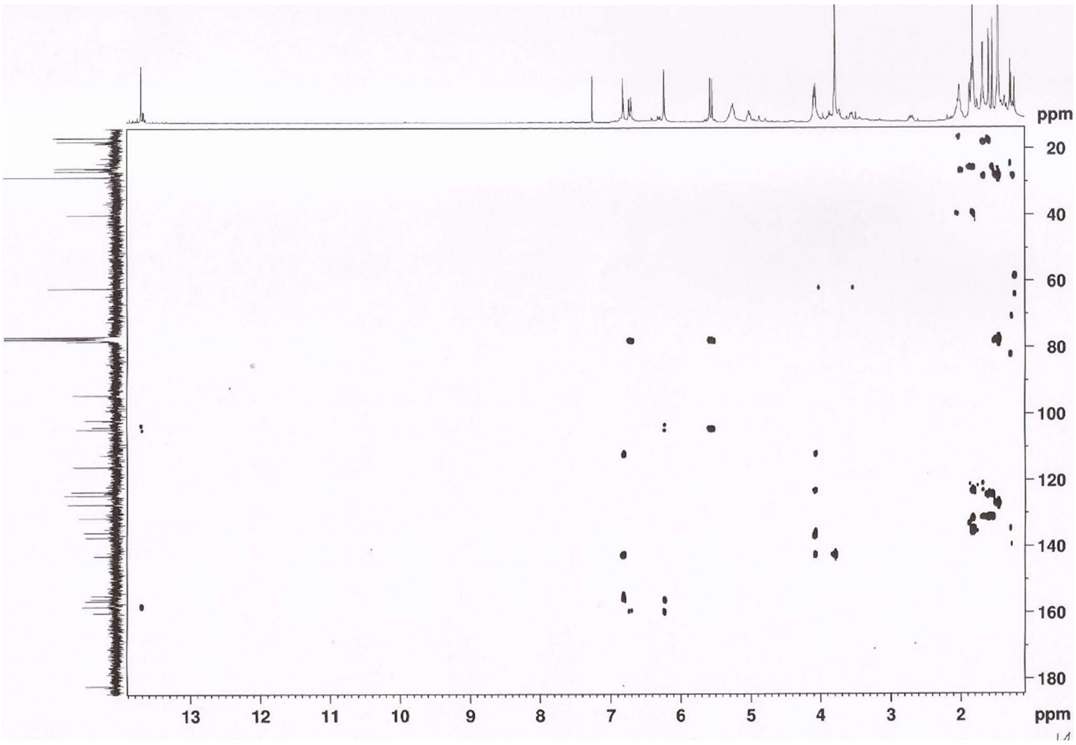

Figure S31. ^{1}H NMR spectrum of fuscaxanthone B (DMSO-*d6*, 300 MHz).

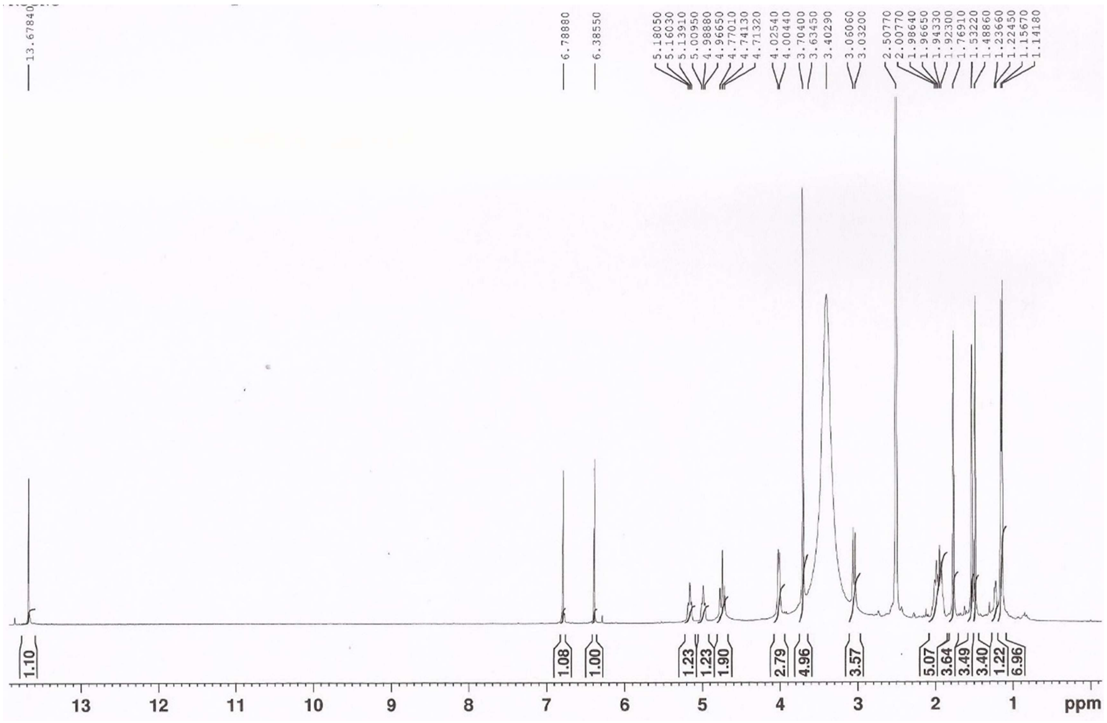

Figure S32. [1]COSY spectrum of fuscaxanthone B (DMSO-*d6*, 300 MHz).

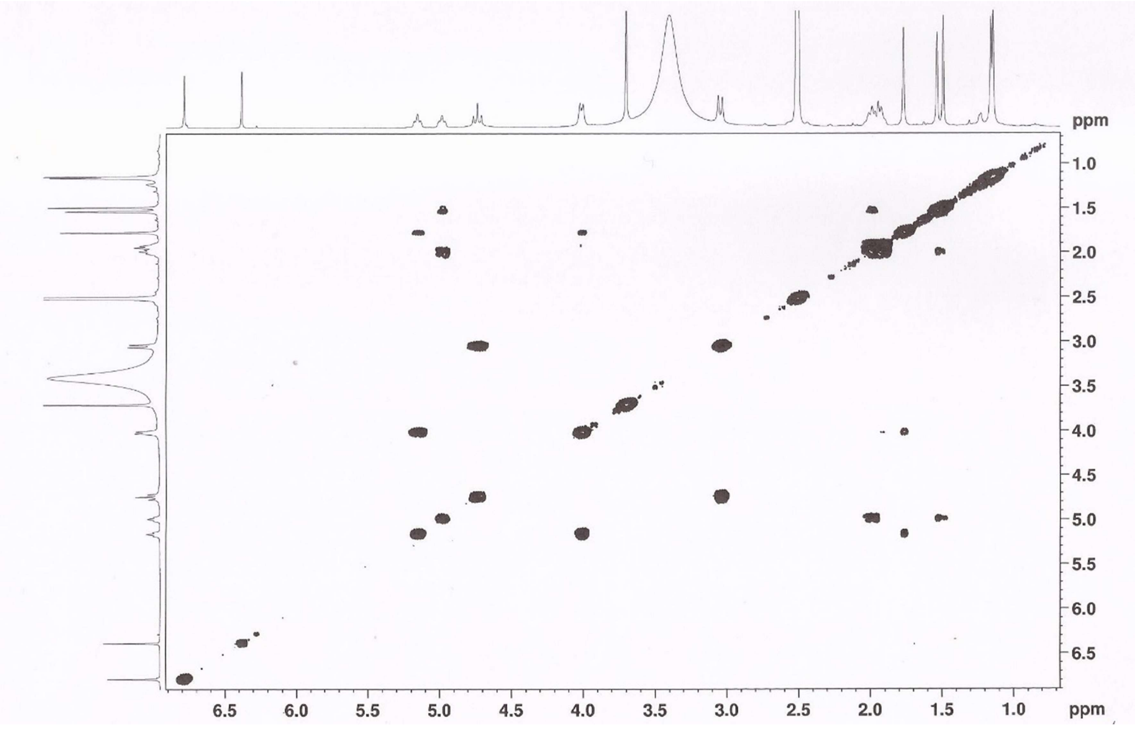

Figure S33. ^{13}C NMR spectrum of fuscaxanthone B (DMSO-*d6*, 300 MHz).

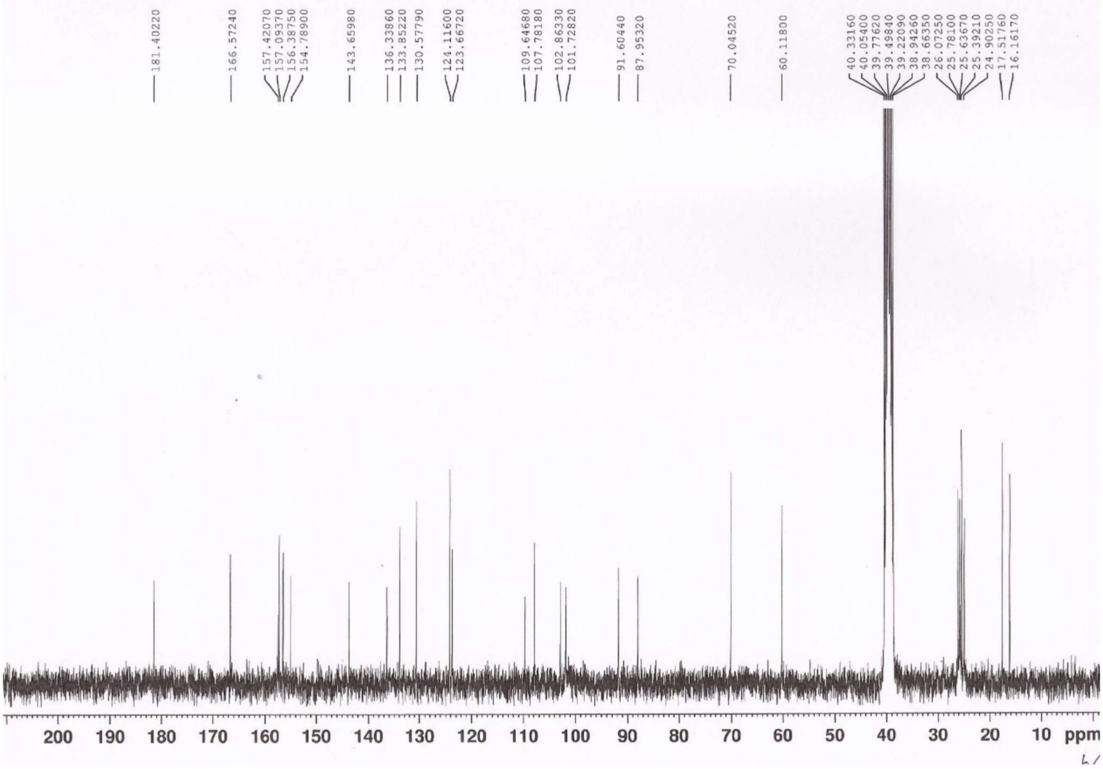

Figure S34. HSQC spectrum of fuscaxanthone B (DMSO-*d6*, 300 MHz).

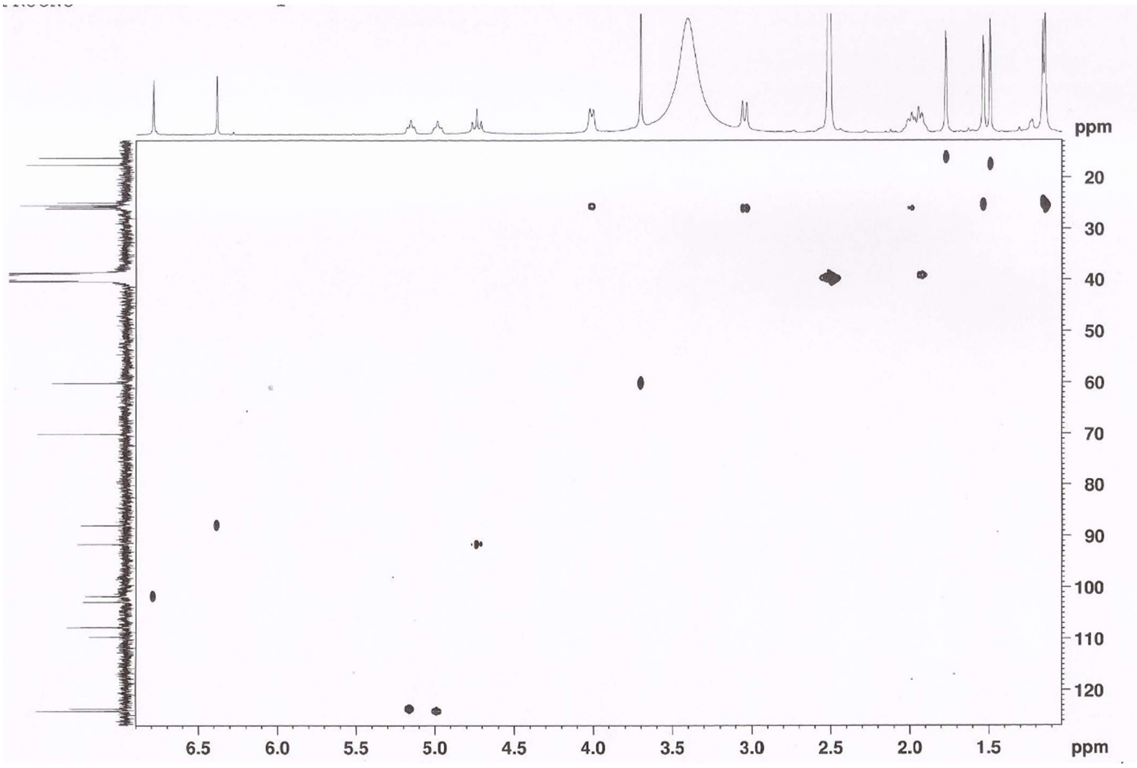

Figure S35. HMBC spectrum of fuscaxanthone B (DMSO-*d6*, 300 MHz).

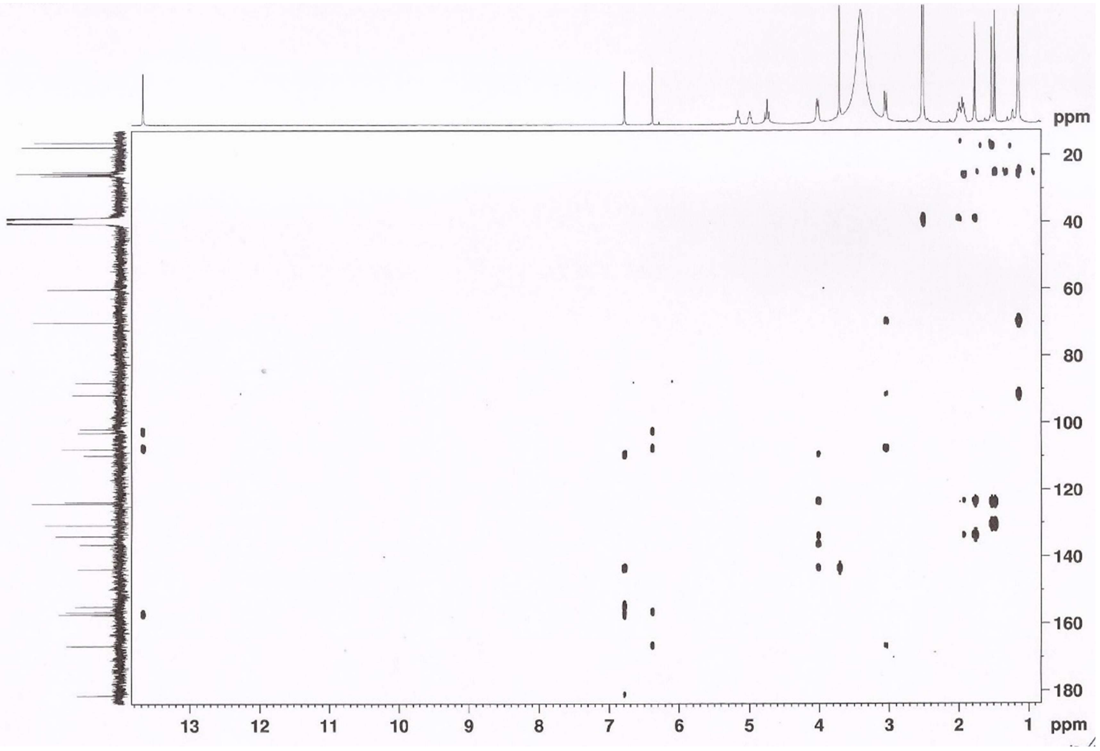

Figure S36. ^{1}H NMR spectrum of xanthochymusxanthone A ($CDCl_3$, 500 MHz).

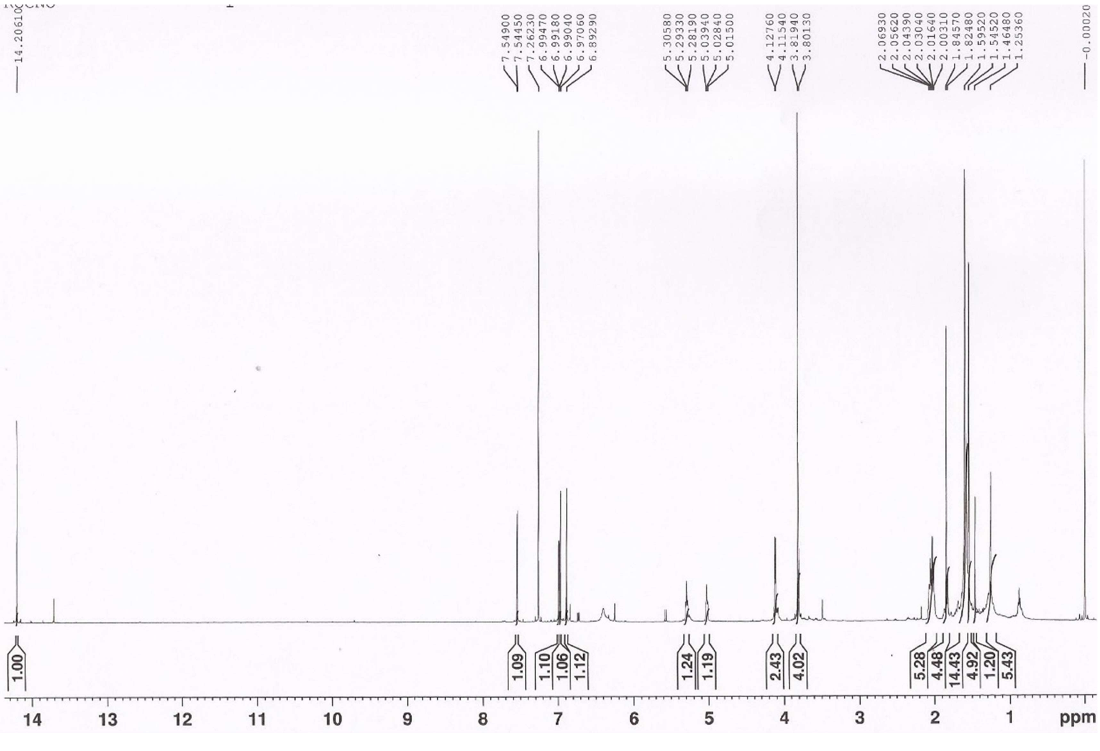

Figure S37. ^{13}C NMR spectrum of xanthochymusxanthone A ($CDCl_3$, 125 MHz).

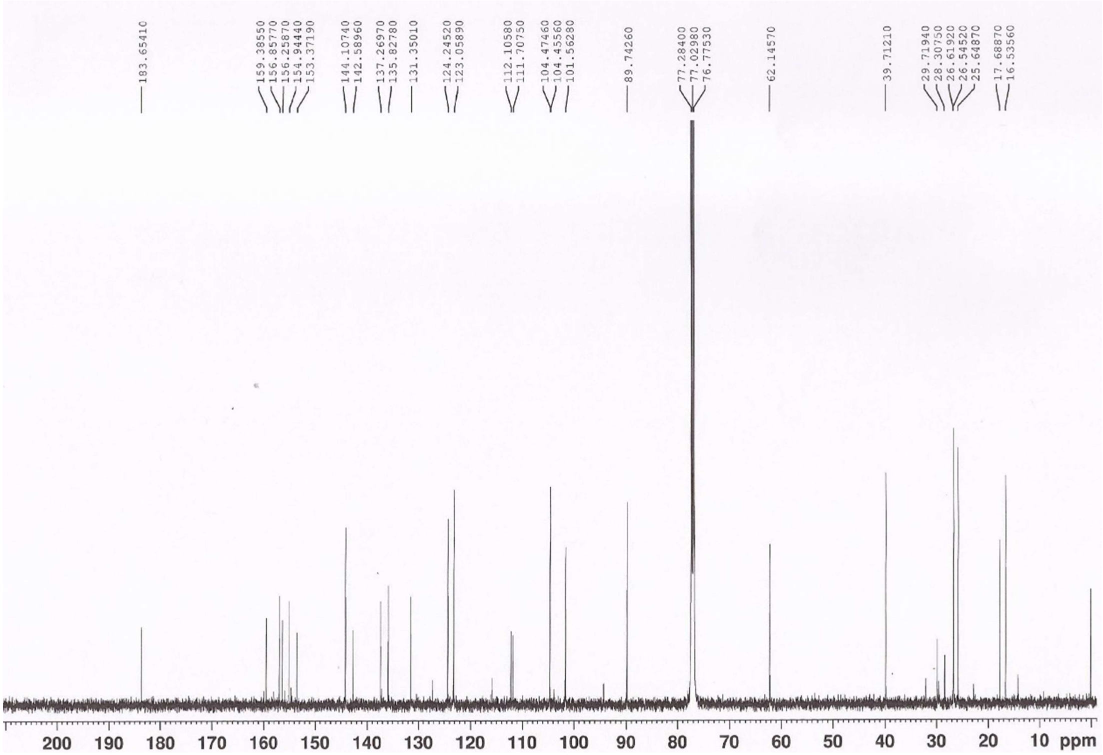

Figure S38. COSY spectrum of xanthochymusxanthone A ($CDCl_3$, 500 MHz).

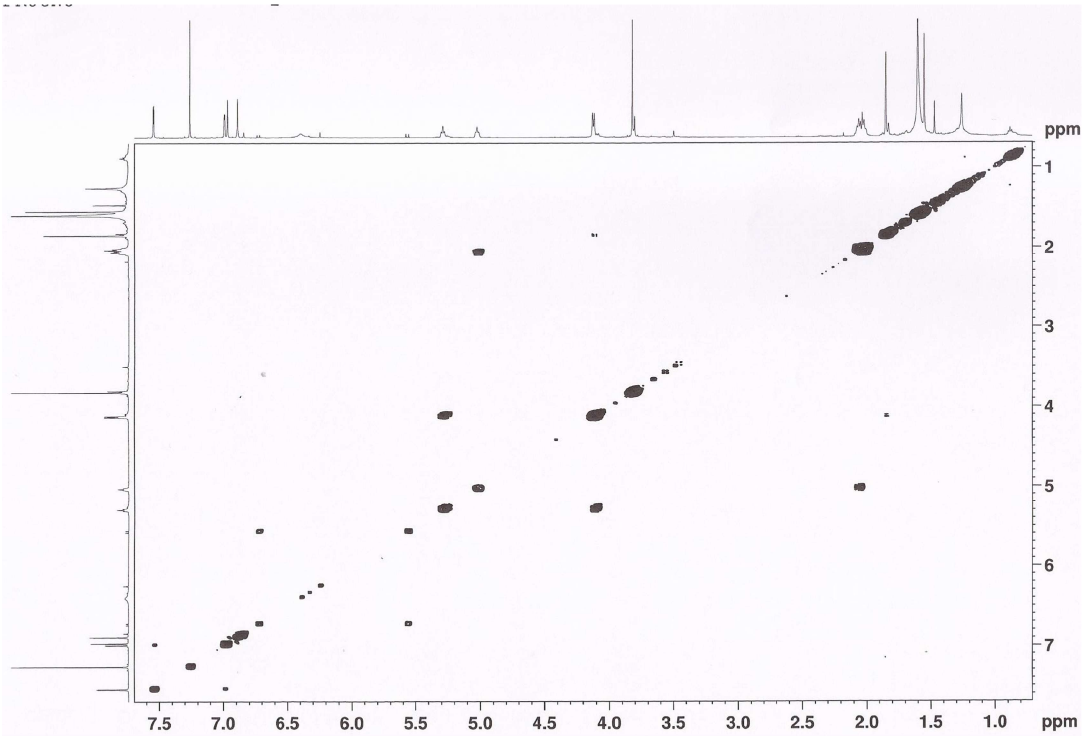

Figure S39. HSQC spectrum of xanthochymusxanthone A ($CDCl_3$, 500 MHz).

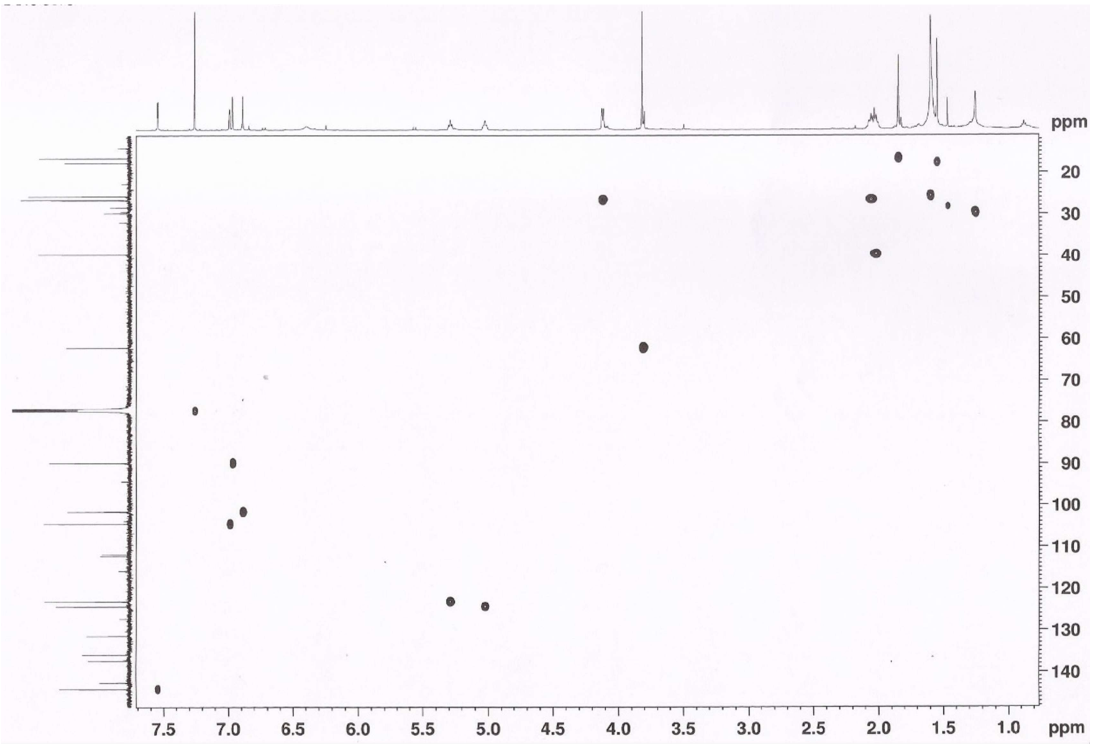

Figure S40. HMBC spectrum of xanthochymusxanthone A ($CDCl_3$, 500 MHz).

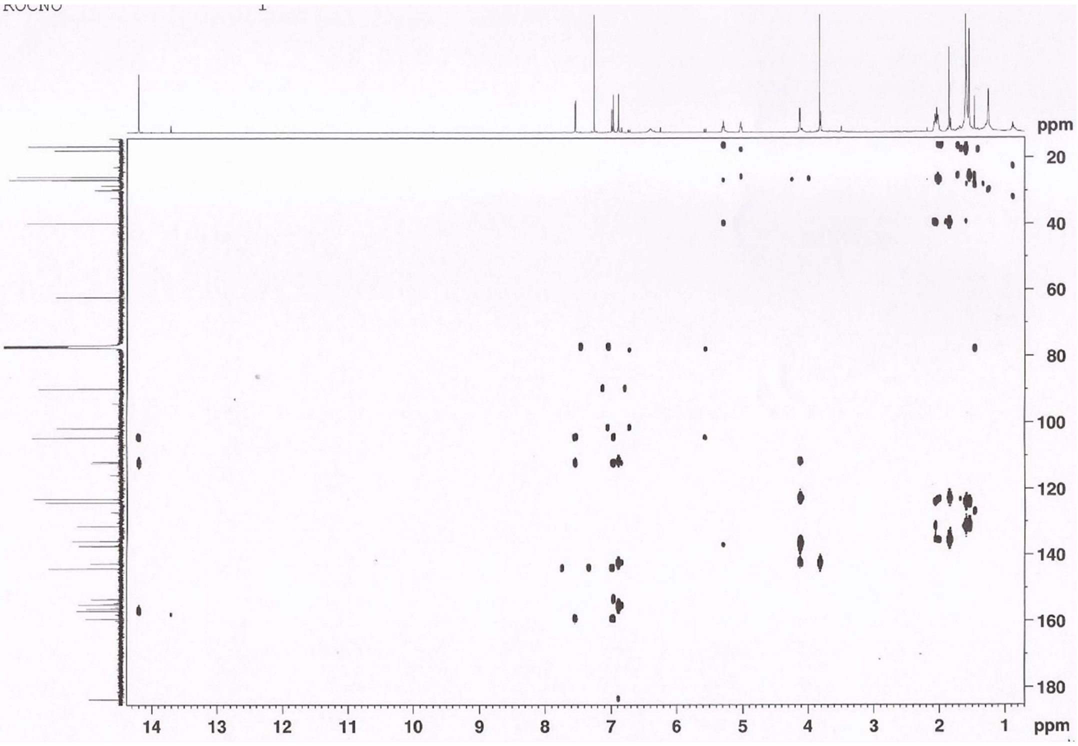

Figure S41. ^{1}H NMR spectrum of 7-*O*-methylgarcinone E ($CDCl_3$, 300 MHz).

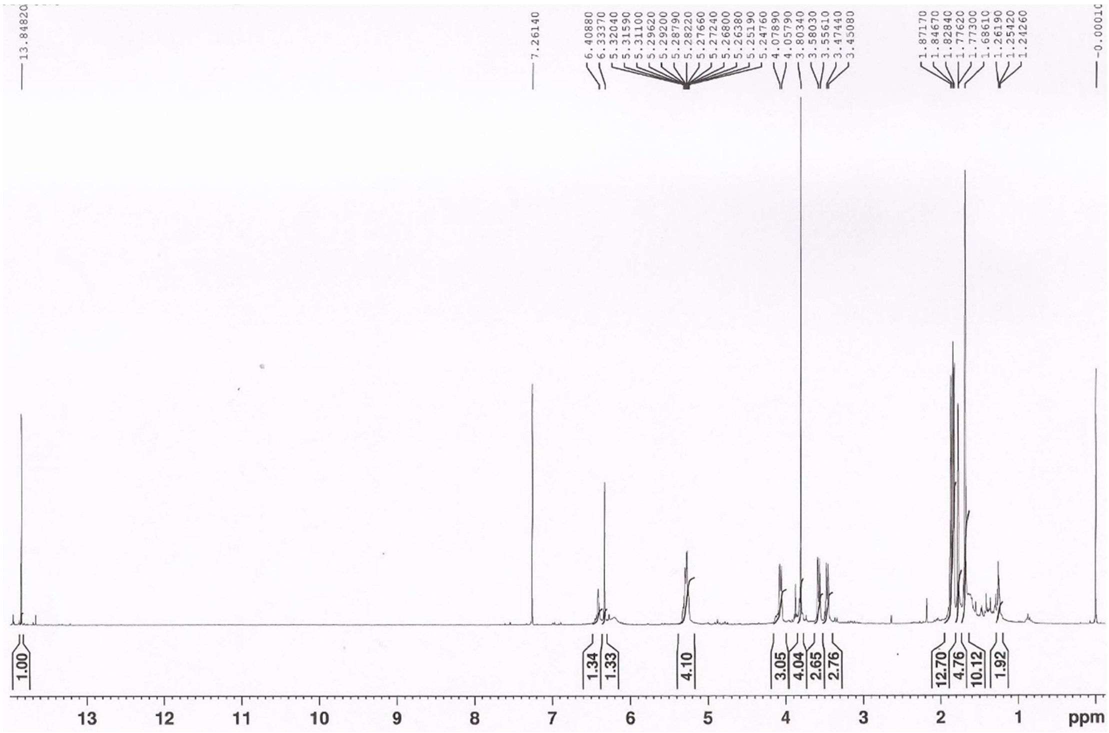

Figure S 42. ^{13}C NMR spectrum of 7-*O*-methylgarcinone E ($CDCl_3$, 75 MHz).

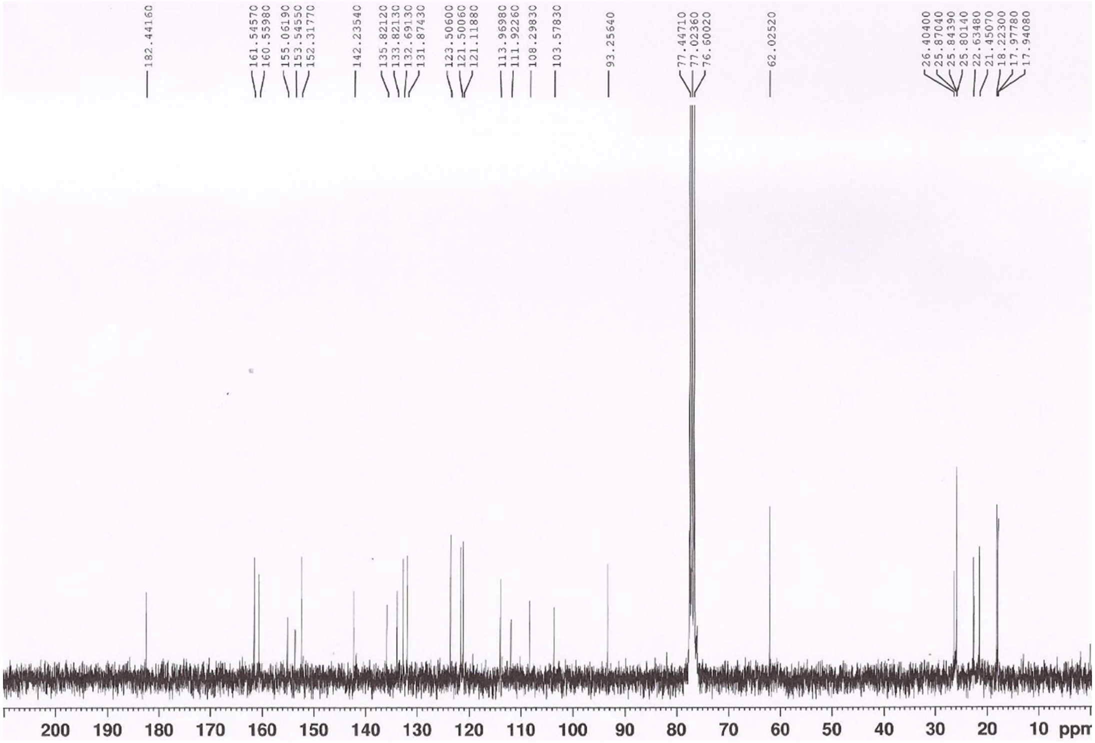

Figure S43. COSY spectrum of 7-*O*-methylgarcinone E ($CDCl_3$, 300 MHz).

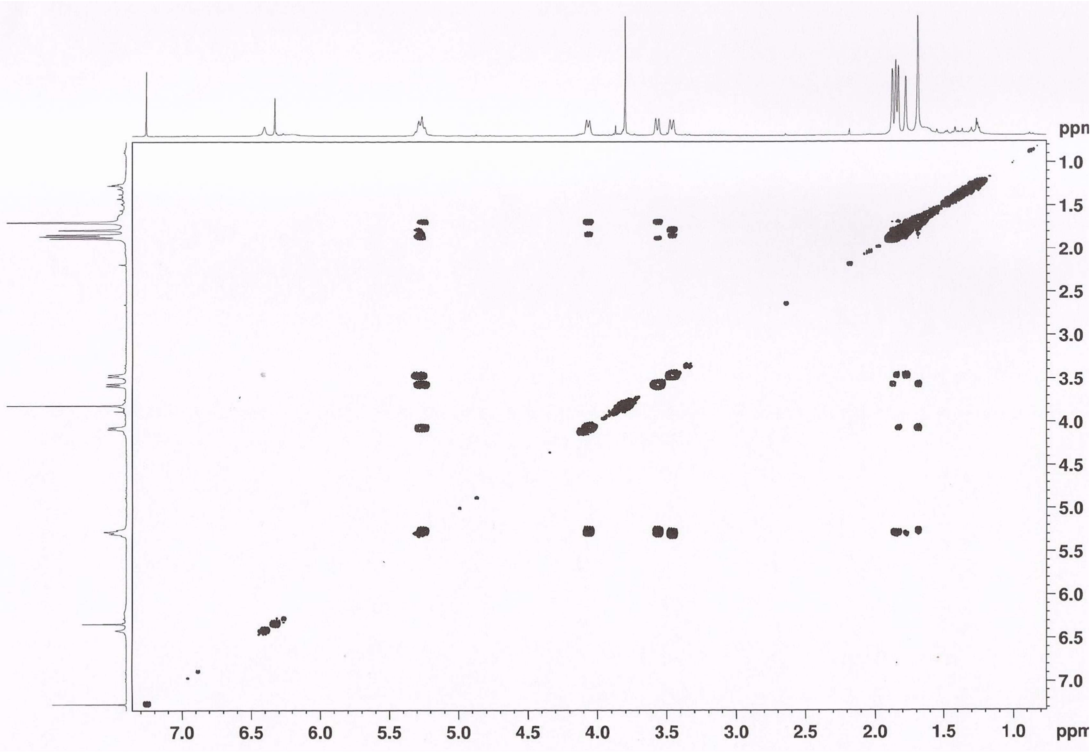

Figure S44. HSQC spectrum of 7-*O*-methylgarcinone E ($CDCl_3$, 300 MHz).

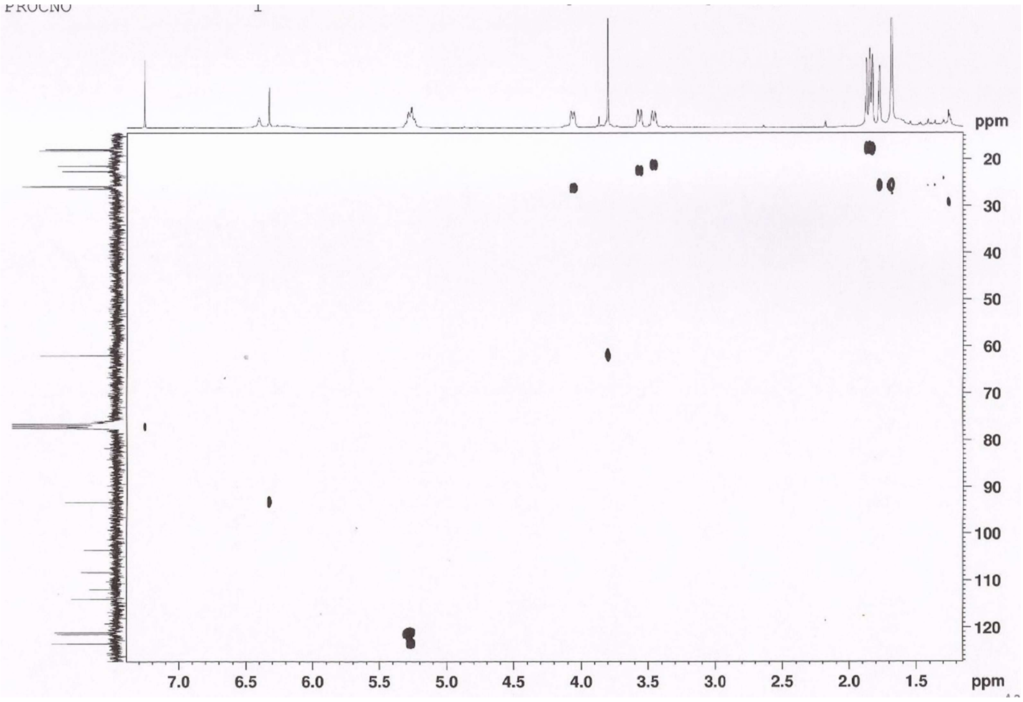

Figure S45. HMBC spectrum of 7-*O*-methylgarcinone E ($CDCl_3$, 300 MHz).

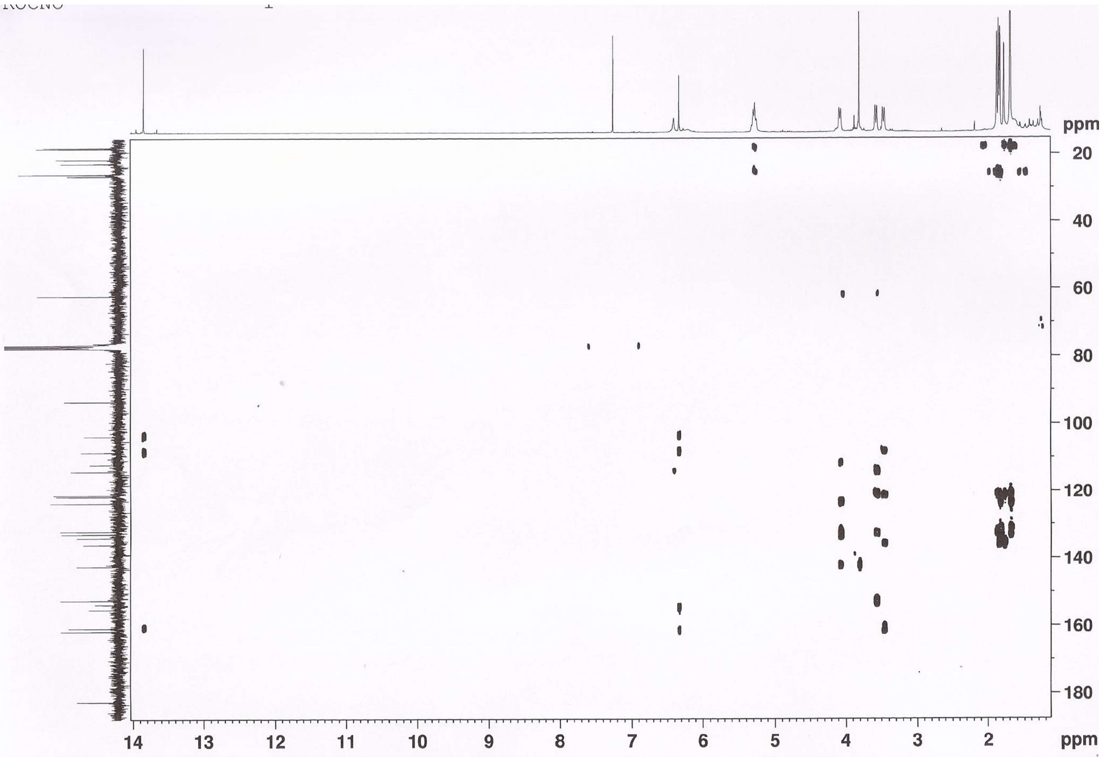

Figure S 46. ^{1}H NMR spectrum of cowagarcione A ($CDCl_3$, 500 MHz).

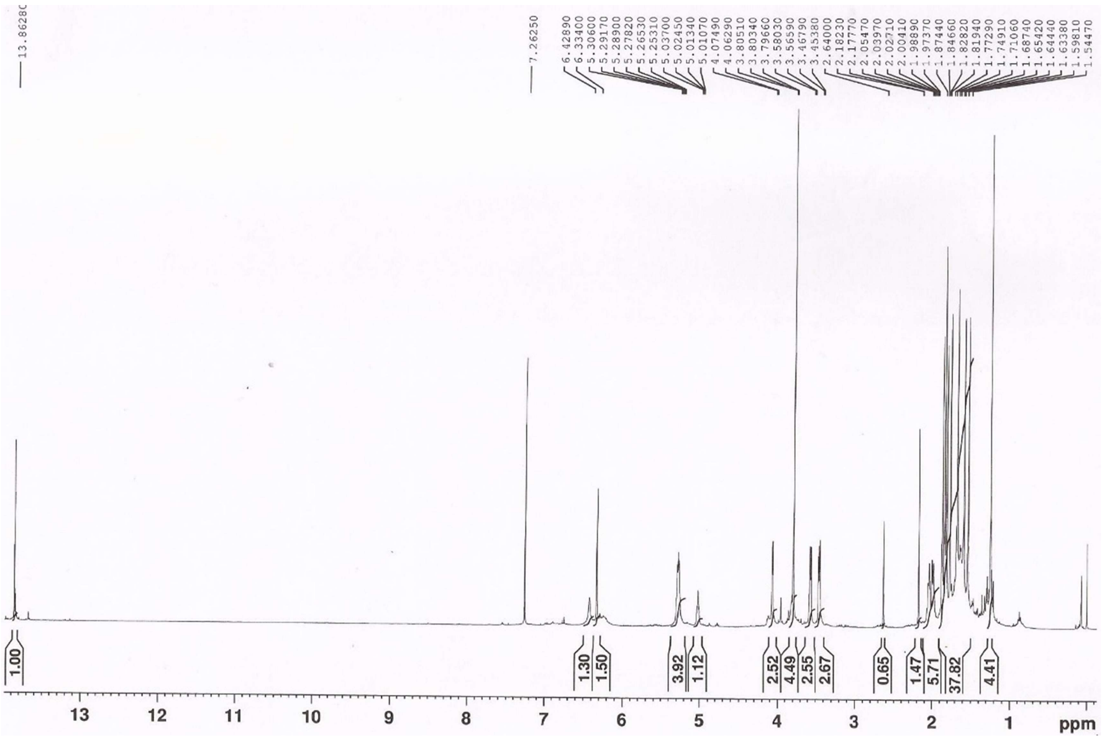

Figure S 47. ^{13}C NMR spectrum of cowagarcione A ($CDCl_3$, 125 MHz).

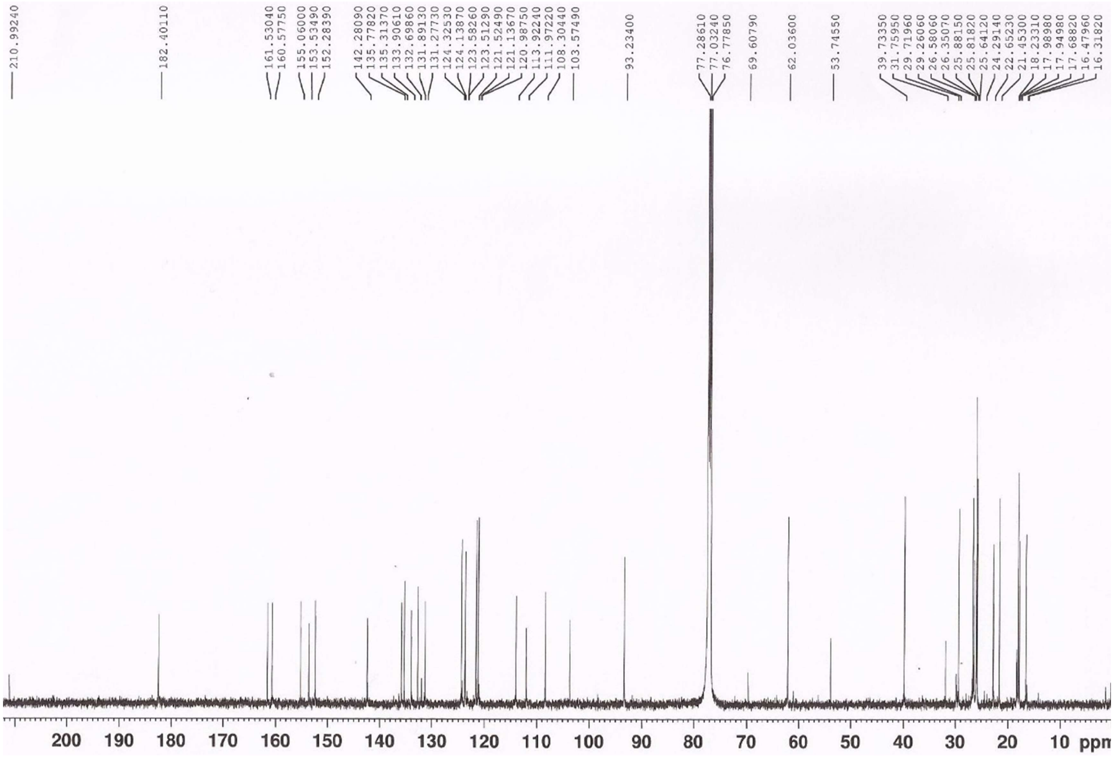

Figure S48. COSY spectrum of cowagarcione A ($CDCl_3$, 500 MHz).

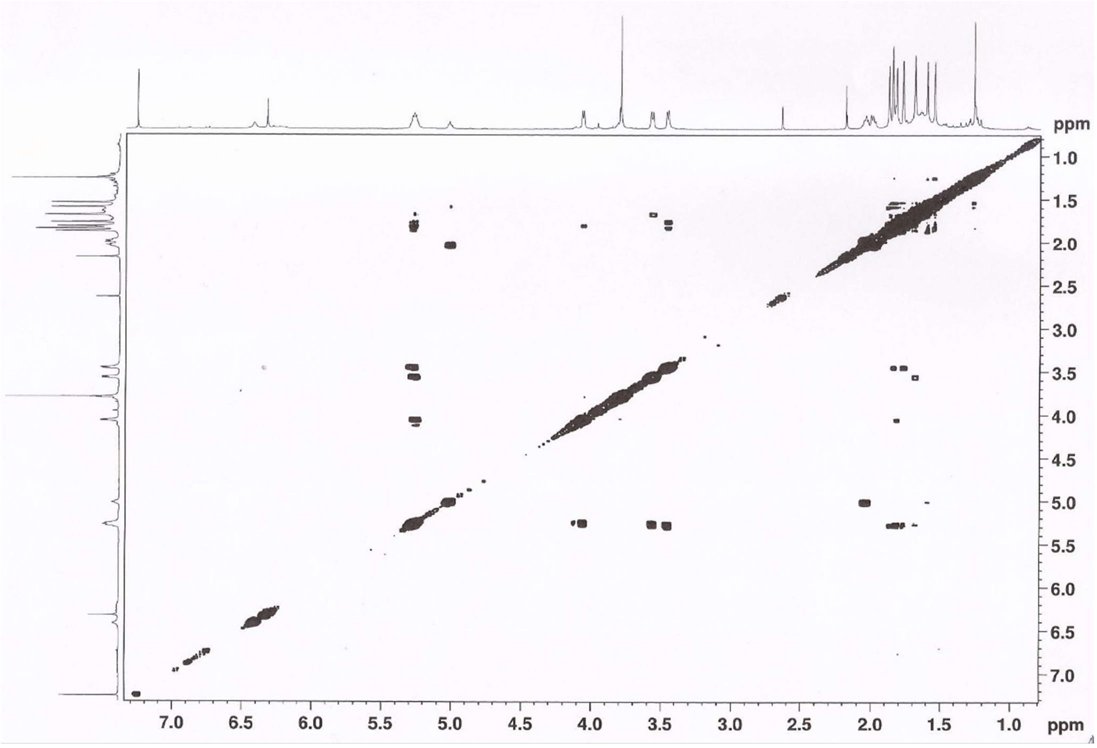

Figure S 49. HSQC spectrum of cowagarcione A ($CDCl_3$, 500 MHz).

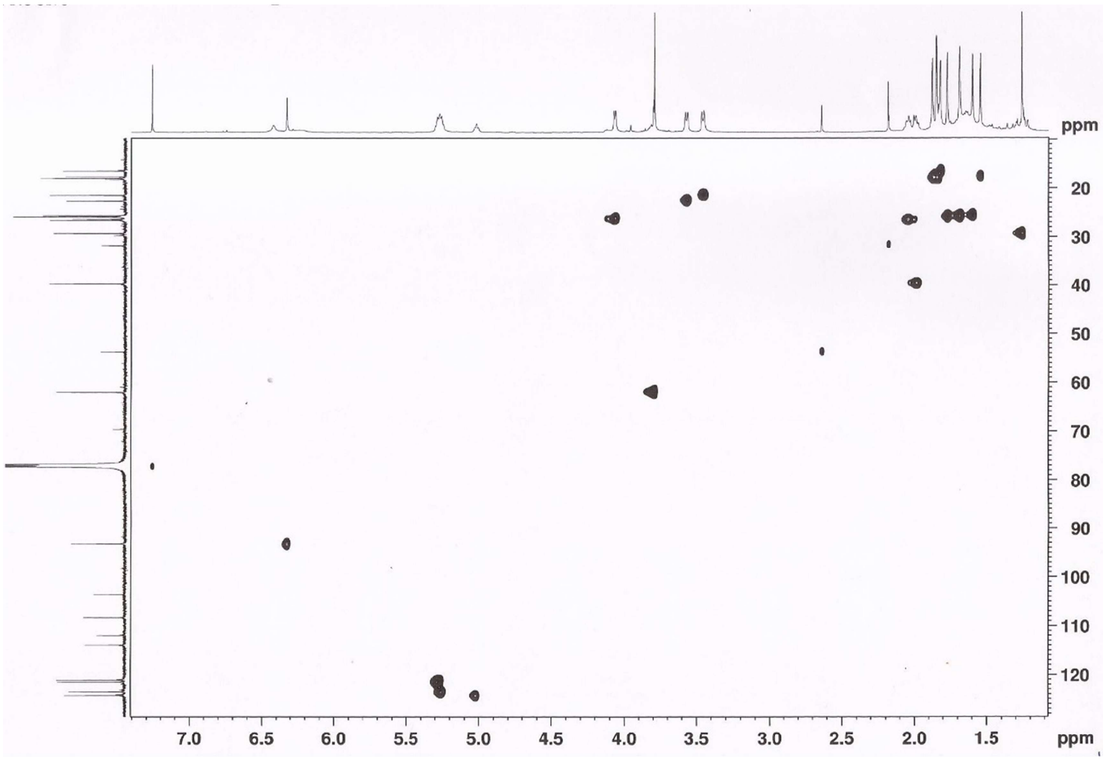

Figure S 50. HMBC spectrum of cowagarcione A ($CDCl_3$, 500 MHz).

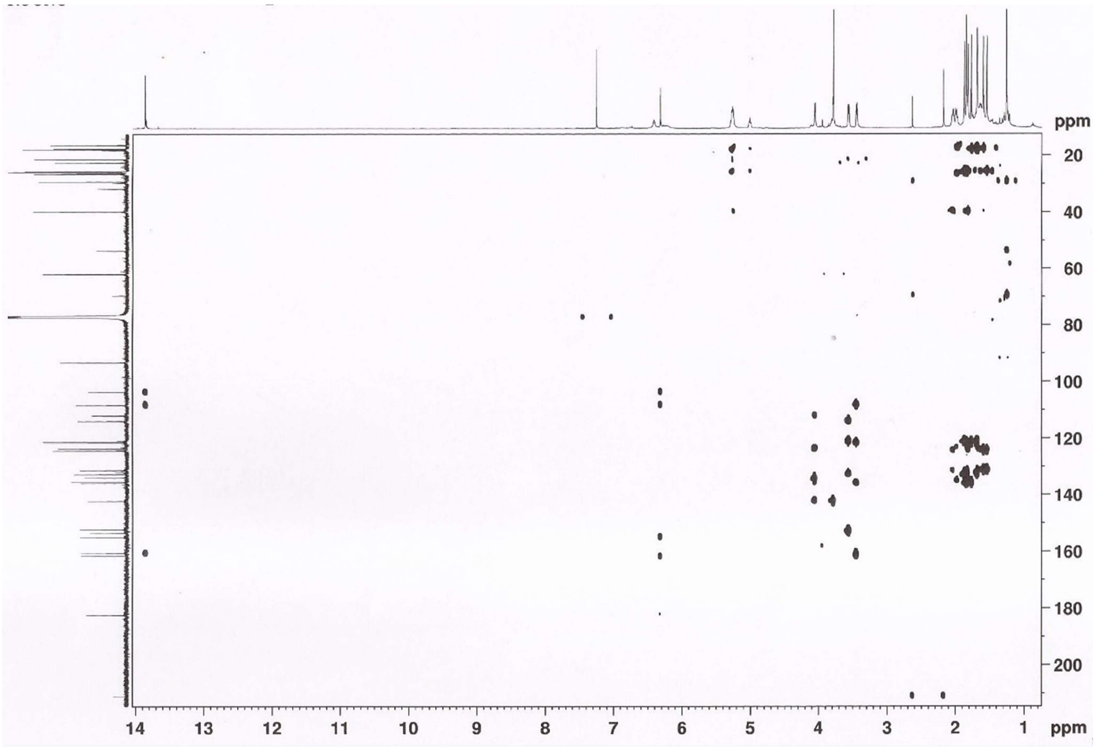

Figure S 51. A flow chart of the experimental protocol with the number of animals used, died, and included in this study*.

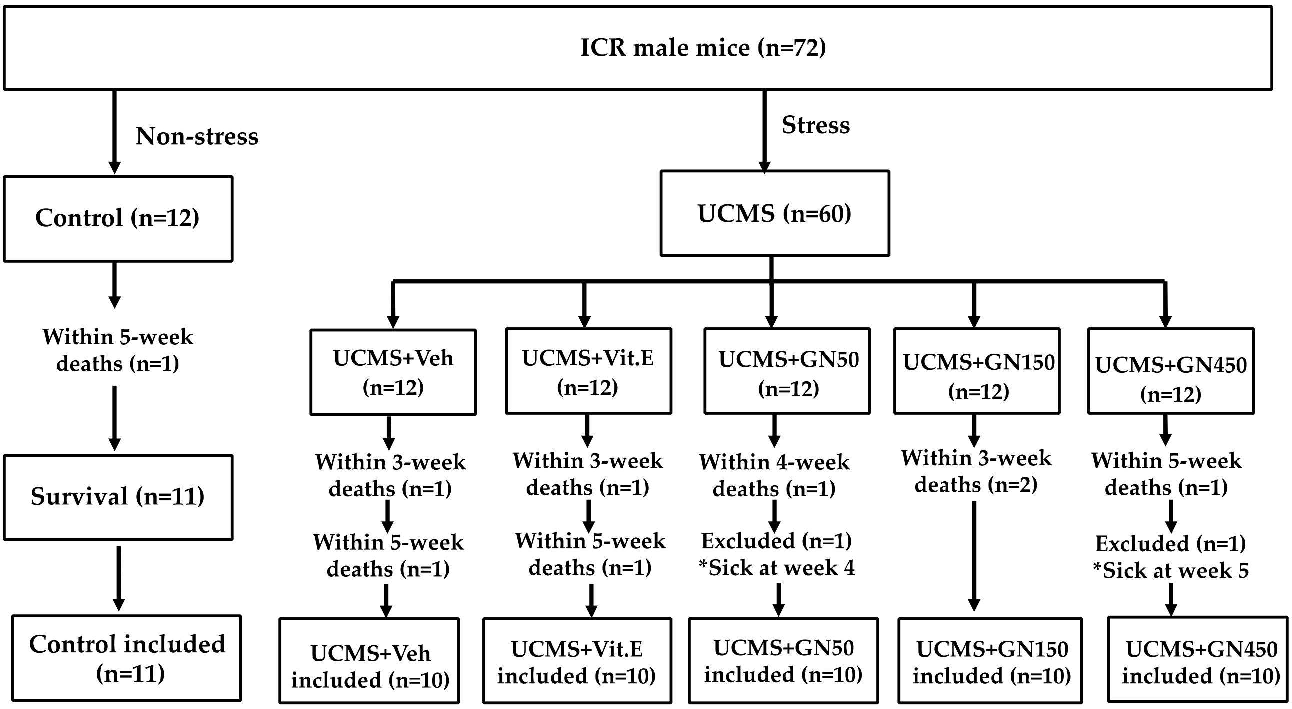

*All animal exclusions, along with the rationale for animal exclusions were summarized in a flowchart describing attrition in each group (ARRIVE guidelines 2.0).

Table S1. ^{1}H and ^{13}C NMR (300 and 75 MHz, $CDCl_3$) and HMBC assignments of cowagarcinone C (**1**).

Position	δ_C, type	δ_H (J in Hz)	COSY	HMBC
1	158.6, C			
2	110.7, C			
3	163.7, C			
4	90.6, CH	6.74, s	-	C-2, 3, 4a, 9 (w), 9a
4a	155.6, C			
5	134.4, C			
6	156.8,C			
7	114.2, CH	6.99, d (8.9)	H-8	C-5, 8a
8	120.8,CH	7.55, d (8.9)	H-7	C-6, 910a
8a	113.1, C			

9	179.6, C			
9a	102.1, C			
10a	150.5, C			
OMe-3	56.5,CH_3	3.94, s		C-3
OMe-5	60.8, CH_3	3.91, s		C-5
1’	20.9, CH_2	3.23, d (7.0)	H-2’, H_3-4’, H_3-5’	C-2, 2’, 3’
2’	122.0, CH	5.13, m	H-1’, H_3-4’, H_3-5’	
3’	130.9, C			
4’	25.5, CH_3	1.62,s	H-1’, 2’	C-2’, 3’, 5’
5’	17.6, CH_3	1.73, s	H-1’, 2’	C-2’, 3’, 4’
OH-1	-	13.19,s		C-1, 2, 9a

Table S2. ^{1}H and ^{13}C NMR (300 and 75 MHz, $CDCl_3$) and HMBC assignments of cowaxanthone (**2**).

Position	δ_C, Type	δ_H (J in Hz)	COSY	HMBC
1	159.4, C			
2	109.8, C			
3	162.5, C			
4	93.0, CH	6.41, s		C-2, 3, 4a, 9, 9a
4a	155.0, C			
5	102.7, CH	6.90. s		C-6, 7, 8a, 9, 10a
6	151.8, C			
7	145.9, C			
8	104.7, CH	7.43, s		C-6, 7, 8a, 9, 10a
8a	111.5, C			
9	178.8, CO			
9a	101.4, C			
10a	154.4, C			
OMe-7	55.9, OMe	3.88, s		C-7
1’	20.9, CH_2	3.23, d (7.0)	H-2’, Me-10	C-1, 2, 3, 2’, 3’
2’	122.2, CH	5.18, m	H-1’, Me-10,	
3’	134.0, C			

4’	39.2, CH_2	1.91, m		C-2’. 3’
5’	26.2, CH_2	2.01, m	H-6’, 4’, Me-8’, 9’	C-4’, 6’
6’	124.1, CH	5.02, m	H-5’, Me-8’, 9’	
7’	130.6, C			
8’	25.4, CH_3	1.57, s	H-5’, 6’	C-6’, 7’, 9’
9’	17.5, CH_3	1.51, s	H-5’, 6’	C-6’, 7’, 8’
10’	15.9, CH_3	1.73, s	H-1’, 2’	C-2’, 3’, 4’
OH-1	-	13.37, s		C-1, 2, 9a
OH-3/6	-	10.86, brs		

Table S3. ^{1}H and ^{13}C NMR (300 and 75 MHz, $CDCl_3$) and HMBC assignments of α-mangostin (**3**)

Position	δ_C, Type	δ_H (J in Hz)	COSY	HMBC
1	160.6, C			
2	108.5, C			
3	161.6, C			
4	93.3, CH	6.29, s		C-2,3,4a, 9a
4a	155.1, C			
5	101.6, CH	6.83, s		C-6,7,8a,9 (w), 10a
6	154.6, C			
7	142.5, C			
8	137.0, C			
8a	112.2, C			
9	182.0, CO			
9a	103.6, C			
10a	155.8, C			
OMe-7	62.1, OCH_3	3.81, s		C-7
1’	21.5, CH_2	3.45, d (7.1)	H-2’, Me-4’, 5’	C-1, 2, 2’, 3, 3’
2’	121.5, CH	5.29, m	H-1’, Me-4’, 5’	
3’	135.8, C			
4’	25.9, CH_3	1.77, d (0.9)		C-2’, 3’, 5’
5’	18.2, CH_3	1.84, s		C-2’, 3’, 4’

1”	26.6, CH_2	4.09, d (6.7)	H-2”, Me-4”, 5”	C-2”, 3”, 7,8,8a
2”	123.2, CH	5.27, s	H-1”, Me-4”, 5”	
3”	135.8, C			
4”	25.8, CH_3	1.69, d (0.9		C-2”, 3”, 5”
5”	17.9 CH_3	1.83, s		C-2”, 3”, 4”
OH-1	-	13.77, s		C-1,2,9a

Table S4. ^{1}H and ^{13}C NMR (300 and 75 MHz, $CDCl_3$) and HMBC assignments of cowanxanthone B (**4**).

Position	δ_C, Type	δ_H (*J* in Hz)	COSY	HMBC
1	160.6, C			
2	108.5, C			
3	161.6, C			
4	93.1, CH	6.32, d (1.0)		C-2, 3, 4a, 9a
4a	155.0, C			
5	98.3, CH	6.73, s		C-4a, 6, 7, 8a, 10a
6	158.1, C			
7	143.9, C			
8	137.3, C			
8a	111.9, C			
9	182.1, CO			
9a	103.8, C			
10a	155.5, C			
OMe-6	56.0, OCH_3	3.96, s		C-6
OMe-7	61.0, OCH_3	3.79, s		C-7
1’	21.5, CH_2	3.46, d (7.1)	H-2’, Me-4’, 5’	C-1, 2, 2’, 3, 3’
2’	121.5, CH	5.29, m	H_2-1’, Me-4’, 5’	
3’	135.7, C			
4’	26.0, CH_3	1.77, d (0.9)		C-2’, 3’, Me-5’
5’	18.2, CH_3	1.85, s		C-2’, 3’, Me-4’
1”	26.2, CH_2	4.13, d (6.5)	H-2”, Me-4”, 5”	C-2”, 3”, 7, 8, 8a
2”	123.2, CH	5.22, m	H_2-1”, Me-4”, 5”	

3”	131.9, C			
4”	25.9, CH_3	1.68, s		C-2”, 3”, Me-5”
5”	17.9, CH_3	1.85, s		C-2”, 3”, Me-4”
OH-1	-	13.84s		C-1, 2, 9a

Table S5. ^{1}H and ^{13}C NMR data of (300 and 75 MHz, DMSO-d6) and HMBC assignment of cowanin (**5**).

Position	δ_C, Type	δ_H (*J* in Hz)	COSY	HMBC
1	159.9, C			
2	109.6, C			
3	162.3, C			
4	92.2, CH	6.33, s		C-2, 3, 4a, 9, 9a
4a	154.2			
5	101.8, CH	6.78, s		C-6, 7, 8a, 9, 10a
6	156.9, C			
7	143.3, C			
8	136.5, C			
8a	110.0, C			
9	181.3, CO			
9a	101.9, C			
10a	154.6, C			
OMe-7	60.1, CH_3	3.69, s		C-7
1’	20.2, CH_2	3.19, m	H-2’, Me-4”, 5”	1, 2, 2’, 3, 3’
2’	122.5, CH	5.17, t (7.1)	H-1’, Me-4’, 5’	
3’	130.3, C			
4’	25.5, CH_3	1.61, s	H-1’, 2’	C2’, 3’, 5’
5’	17.7, CH_3	1.72, s	H-1’, 2’	C-2’, 3’, 4’
1”	25.6, CH_2	4.00, d (6.3)	H-2”, Me-10”	C-2”, 3”, 7, 8, 8a
2”	123.7, CH	5.15, t (6.3)	H-1”, Me-10”	C-8
3”	133.8, C			
4”	39.2, CH_2	1.92, m	H-5”, 6”	C-5”, 6”, 10”
5”	26.1, CH_2	1.96, m	H-4”, 6”, Me-8”, 9”	C-4”, 6”, 10”
6”	124.1, CH	4.97,d (6.3)	H-5”, Me-8”, 9”	
7”	130.5, C			
8”	25.3, CH_3	1.51, s	H-5”, 6”	C-6”, 7”, 9”
9”	17.4, CH_3	1.47, s	H-1”, 5”	C-6”, 7”, 8”
10”	16.1, CH_3	1.76, s	H-1”, 2”	C-2”, 3”, 4”
OH-1		13.71, s		C-1, 2, 9a,
OH-3/7		10.86, brs		

Table S6. ^{1}H and ^{13}C NMR data of (300 and 75 MHz, $CDCl_3$) and HMBC assignment of fuscaxanthone A (**6**).

Position	δ_C, Type	δ_H (*J* in Hz)	COSY	HMBC
1	157.9, C			
2	104.6, C			

3	159.8, C			
4	94.1, CH	6.23, s		C-2, 3, 4a, 9a
4a	155.7, C			
5	101.7, C	6.82, s		C-6, 7, 8a, 9, 10a
6	156.3, C			
7	142.7, C			
8	137.1, C			
8a	113.0, C			
9	181.9, CO			
9a	103.7, C			
1’	115.7, CH	6.72, d (10.0)	H-2’	C-3, 3’
2’	127.1, CH	5.57, d (10.0)	H-1’	C-2, 3’
3’	77.9, C			
4’	28.3, CH_3	1.46, s		
5’	28.3, CH_3	1.46, s		
1”	26.6, CH_2	4.08, d (6.1)	H-2”, Me-10”	C-2”, 3”, 7, 8, 8a
2”	123.2, CH	5.27, m	H-1”, Me-10”	
3”	135.6, C			
4”	39.7, CH_2	2.02, m		C-5”
5”	26.5 CH_2	2.04, m		C-4”, Me-10”
6”	124.3, CH	5.02,m	Me-8”, 9”H-4”,	
7”	131.1, C			
8”	25.6, CH_3	1.60, s		Me-9”
9”	17.7, CH_3	1.54, s		Me-8”
10”	16.5, CH_3	1.82, s		C-4”, 5”
OMe-7	62.0, CH_3	3.79, s		C-7
OH-1	-	13.72, s		C-1, 2, 9a

Table S7. ^{1}H and ^{13}C NMR (300 and 75 MHz, DMSO-d_6) and HMBC assignment of fuscaxanthone B (7).

Position	δ_C, Type	δ_H (*J* in Hz)	COSY	HMBC
1	157.4, C			
2	107.8, C			
3	166.6, C			
4	88.0, CH	6.39, s		C-2, 3, 4a, 9a
4a	156.4, C			
5	101.7, CH	6.73, s		C-6, 7, 8a, 9, 10a
6	157.1, C			
7	143.7, C			
8	136.3, C			
8a	109.6, C			
9	181.4, CO			
9a	102.9, C			
10a	154.8, C			
1’	26.1, CH_2	3.05, d (8.6)		C-2, 3, 3’, 4’
2’	91.6, CH	4.74, t (8.6)		
3’	70.0, C-3’			
4’	24.9, CH_3	1.14,s		C-C-2’, 3’, 5’
5’	25.8, CH_3	1.16, s		C-2’, 3’, 4’

1”	25.6, CH_2	4.01, d (6.3)	H-2”, Me-10”	C-2”, 3”, 7, 8, 8a
2”	123.6, CH	5.16 t (6.1)	H-1”, Me-10”	
3”	133.9, C			
4”	39.2, CH_2	1.94, m		C-2”, 3”, 5”
5”	26.1, CH_2	1.99, m	H-6”	C-4”
6”	124.1, CH	4.99, t (6.7)	H-5”, Me-8”, 9”	
7”	130.6, C			
8”	25.4, CH_3	1.53, s	H-6”	C-6”, 7”, 9”
9”	17.5, CH_3	1.49, s	H-6”	C-6”, 7”, 8”
10”	16.2, CH_3	1.77, s	H-2”	C-2”, 3”, 4”
OMe-7	60.1, OCH_3	3.70, s		C-7
OH-1	-	13.68s		C-1, 2, 4a

Table S8. ^1H and ^{13}C NMR (500 and 125 MHz, $CDCl_3$) and HMBC assignment of xanthochymusxanthone A (**8**).

Position	δ_C, Type	δ_H (*J* in Hz)	COSY	HMBC
1	156.9, C			
2	112.1, C			
3	159.4, C			
4	89.7, CH	6.97, s		C-4a, 9a
4a	153.3, C			C-6, 7, 8a, 9
5	101.6, CH	6.89, s		
6	156.3,C			
7	142.6, C			
8	137.3, C			
8a	111.7, C			
9	183.7, CO			
9a	104.5, C			
10a	154.9, C			
11	104.5, CH	6.99, dd (2.3, 0.8)	H-12	C-3, C-12
12	144.2, CH	7.55, d (2.3)	H-11	C-2, 3, 11
1’	26.6, CH_2	4.12, d (6.6)	H-2’	C-7, 8, 8a
2’	123.1, CH	5.29, t (6.3)	H-1’	C-8
3’	135.8, C			
4’	39.2, CH_2	2.02, m	H-5’	C-2’, 3’
5’	26.5, CH_2	2.06, m	H-4’, 6’	C-6’, 7’
6’	124.2, CH	5.03, t (6.7)	H-5’	
7’	131.4, C			
8’	25.6, CH_3	1.60, s		C-6’, 7’
9’	17.7, CH_3	1.55, s		C-6’. 7’
10’	16.5, CH_3	1.85, s		C-2’, 3’
OMe-7	62.1, OCH_3	3.82, s		C-7
OH-1	-	14.24, s		C-1, 2, 9a

Table S9. ^{1}H and ^{13}C NMR (500 and 125 MHz, $CDCl_3$) and HMBC assignment of 7-*O*-methylgarcinone E (**9**).

Position	δ$_C$, Type	δ$_H$ (*J* in Hz)	COSY	HMBC
1	160.6, C			
2	103.6, C			
3	161.5, C			
4	93.3, CH	6.33, s		C-2, 3, 4a, 9a
4a	152.3, C			
5	114.0, C			
6	153.1, C			
7	142.2, C			
8	133.8, C			
8a	111.9, C			
9	182.4, CO			
9a	108.3, C			
10a	152.3, C			
1'	21.5, CH_2	3.46, d (7.1)	H-2'. Me-4', 5'	C-1, 2, 3, 2', 3'
2'	121.5, CH	5.29, m		
3'	135.8, C			
4'	25.8, CH_3	1.77, d (0.1)		C-2', 3'
5'	18.0, CH_3	1.85, s		C-3'
1"	22.6, CH_2	3.57, d (7.3)	H-2". Me-4", 5"	C-2", 3", 5, 10a
2"	121.1, CH	5.27, m		
3"	132.7, C			
4"	25.8, CH_3	1.60,s		C-2", 3"
5"	18.2, CH_3	1.87, s		C-2", 3"
1"'	26.4, CH_2	4.07, d (6.3)	H-2"'. Me-4"', 5"'	C-2"', 7, 8, 8a
2"'	123.5, CH	5.27, m		
3"'	131.9			
4"'	26.0, CH_3	1.60, s		C-2"', 3"'
5"'	17.9, CH_3	1.83, s		C-2"', 3"'
OMe-7	62.0, OCH_3	3.80, s		C-7
OH-1	-	13.85, s		C-1, 2, 9a
OH-6	-	6.41, brs		C-5

Table S10. ^{1}H and ^{13}C NMR (500 and 125 MHz, $CDCl_3$) and HMBC assignment of cowagarcinone A (**10**).

Position	δ$_C$, Type	δ$_H$ (*J* in Hz)	COSY	HMBC
1	160.6, C			
2	108.3, C			
3	161.5, C			
4	93.2, CH	6.33s		C-2, 3, 4a, 9a
4a	155.1			
5	113.9, C			
6	152.3, C			
7	142.3, C			

8	133.9, C			
8a	112.0, C			
9	182.4CO			
9a	103.6, C			
10a	153.5, C			
1’	21.5, CH_2		H-2’	C-2, 2’, 3’
2’	121.5, CH	5.29, m	H-1’	
3’	135.8, C			
4’	25.9, CH_3	1.77,s		C-2’, 3’
5’	17.9, CH_3	1.85, s		C-2’, 3’
1”	22.7, CH_2	3.57, d (7.2)	H-2”, Me-4”	C-5, 2”, 3”
2”	121.1, CH	5.26, m	H-1”	
3”	132.7, C			
4”	25.8, CH_3	1.69, s	H-5”’	C-2”, 3”
5”	18.0, CH_3	1.87, s	H-4”’, 6”’	C-2”, 3”
1”’	26.4, CH_2	4.07, d (6.0)	H-2”’, Me-10”’	C-2”’, 7, 8, 9a
2”’	123.6, CH	5.26, m		
3”’	135.3, C			
4”’	39.7, CH_2	1.99, m	H-5”’	C-3”’
5”’	26.6, CH_2	2.04, m	H-4”’	C-4”’
6”’	124.3, CH	5.02,m		
7”’	1313, C			
8”’	25.6, CH_3	1.60,s		C-6”’, 7”’
9”’	17.7, CH_2	1.54, s		C-6”’, 7”’
10”’	16.5, CH_3	1.82, s		C-2”’, 3”’
OMe-7	62.0, OCH_3	3.80, s		C-7
OH-1		13.86, s		C-1, 2, 9a

1. Statistical Analysis Effect of GN bark resin on UCMS-Induced Cognitive Deficit Behavioral Using Y-maze test

Table S11. One-way analysis of variance (ANOVA) test of Y-maze test.

Group comparison	Statistical Analysis	
non-stress group vs. CMS + vehicle group	Paired *t-test*	
	P <0.001	
	ANOVA followed by Tukey's post hoc test	
	P	*F* ($DF_{between\ group}$, $DF_{residual}$
All group	<0.001	*F*(4,46)=10.448
UCMS + vehicle group vs. CMS + Vit E 100 group	<0.001	
UCMS + vehicle group vs. CMS + GN50 group	0.081	
UCMS + vehicle group vs. CMS + GN150 group	0.005	

UCMS + vehicle group vs. CMS + GN450 group	<0.001	

2. Statistical Analysis Effect of GN bark resin on UCMS-Induced Cognitive Deficit Behavioral Using Novel Object Recognition Test (NORT)

Table S12. One-way analysis of variance (ANOVA) test of novel object recognition test (NORT).

Group comparison	Statistical Analysis	
The sample phase trial	ANOVA followed by Tukey's post hoc test	
	Not significant P=0.329	
The test phase trial	Statistical Analysis	
non-stress group vs. CMS + vehicle group	Paired *t-test*	
	P <0.001	
	ANOVA followed by Tukey's post hoc test	
	P	F ($DF_{between\ group}$, $DF_{residual}$
All group	<0.001	F(4,46)=10.448
UCMS + vehicle group vs. CMS + Vit E 100 group	<0.001	
UCMS + vehicle group vs. CMS + GN50 group	0.721	
UCMS + vehicle group vs. CMS + GN150 group	0.035	
UCMS + vehicle group vs. CMS + GN450 group	<0.001	
UCMS + GN 50 vs. UCMS + 450 group	0.006	

3. Statistical Analysis Effect of GN bark resin on UCMS-Induced Cognitive Deficit Behavioral Using Morris Water Maze Test (MWMT)

Table S13.1. One-Way Repeated Measures ANOVA test of MWMT on the training test.

Day 1		
Group comparison	Statistical Analysis	
non-stress group vs. CMS + vehicle group	Paired *t-test*	
	Not significant P = 0.187	
	ANOVA followed by Tukey's post hoc test	
	P	F ($DF_{between\ subject}$,$DF_{between\ treatment}$, $DF_{residual}$)
All group	0.876	F(10,4,36)=0.876
UCMS + vehicle group vs. CMS + Vit E 100 group	-	
UCMS + vehicle group vs. CMS + GN50 group	-	
UCMS + vehicle group vs. CMS + GN150 group	-	

UCMS + vehicle group vs. CMS + GN450 group	-	
Day 2		
Group comparison	Statistical Analysis	
non-stress group vs. CMS + vehicle group	Paired *t-test*	
	<0.001	
	ANOVA followed by Tukey's post hoc test	
	P	*F* ($DF_{between\ subject}$, $DF_{between\ treatment}$, $DF_{residual}$)
All group	0.451	*F*(10,4,36)=0.942
UCMS + vehicle group vs. CMS + Vit E 100 group	-	
UCMS + vehicle group vs. CMS + GN50 group	-	
UCMS + vehicle group vs. CMS + GN150 group	-	
UCMS + vehicle group vs. CMS + GN450 group	-	
Day 3		
Group comparison	Statistical Analysis	
non-stress group vs. CMS + vehicle group	Paired *t-test*	
	<0.001	
	ANOVA followed by Tukey's post hoc test	
	P	*F* ($DF_{between\ subject}$, $DF_{between\ treatment}$, $DF_{residual}$)
All group	<0.001	*F*(10,4,36)=16.656
UCMS + vehicle group vs. CMS + Vit E 100 group	<0.001	
UCMS + vehicle group vs. CMS + GN50 group	<0.001	
UCMS + vehicle group vs. CMS + GN150 group	<0.001	
UCMS + vehicle group vs. CMS + GN450 group	<0.001	
Day 4		
Group comparison	Statistical Analysis	
non-stress group vs. CMS + vehicle group	Paired *t-test*	
	<0.001	
	ANOVA followed by Tukey's post hoc test	
	P	*F* ($DF_{between\ subject}$, $DF_{between\ treatment}$, $DF_{residual}$)
All group	<0.001	*F*(10,4,36)=30.030
UCMS + vehicle group vs. CMS + Vit E 100 group	<0.001	
UCMS + vehicle group vs. CMS + GN50 group	<0.001	
UCMS + vehicle group vs. CMS + GN150 group	<0.001	
UCMS + vehicle group vs. CMS + GN450 group	<0.001	

Day 5		
Group comparison	Statistical Analysis	
non-stress group vs. CMS + vehicle group	Paired *t-test*	
	<0.001	
	ANOVA followed by Tukey's post hoc test	
	P	*F* ($DF_{between\ subject}$,$DF_{between\ treatment}$, $DF_{residual}$)
All group	<0.001	*F*(10,4,36)=17.495
UCMS + vehicle group vs. CMS + Vit E 100 group	<0.001	
UCMS + vehicle group vs. CMS + GN50 group	<0.001	
UCMS + vehicle group vs. CMS + GN150 group	<0.001	
UCMS + vehicle group vs. CMS + GN450 group	<0.001	

Table S13.2 One-way analysis of variance (ANOVA) test of MWMT on the probe test.

Group comparison	Statistical Analysis	
non-stress group vs. CMS + vehicle group	Paired *t-test*	
	P <0.001	
	ANOVA followed by Tukey's post hoc test	
	P	*F* ($DF_{between\ group}$, $DF_{residual}$
All group	<0.001	*F*(4,46)=9.958
UCMS + vehicle group vs. CMS + Vit E 100 group	<0.001	
UCMS + vehicle group vs. CMS + GN50 group	0.283	
UCMS + vehicle group vs. CMS + GN150 group	<0.05	
UCMS + vehicle group vs. CMS + GN450 group	<0.001	

4. Statistical Analysis Effect of GN bark resin on the UCMS-Induced Changes Lipid peroxidation in the Frontal cortex and Hippocampus

Table S14.1 One-way analysis of variance (ANOVA) test of UCMS-Induced lipid peroxidation in frontal cortex.

Group comparison	Statistical Analysis	
non-stress group vs. CMS + vehicle group	Paired *t-test*	
	P <0.001	
	ANOVA followed by Tukey's post hoc test	
	P	*F* ($DF_{between\ group}$, $DF_{residual}$
All group	<0.001	*F*(4,16)=12.979
UCMS + vehicle group vs. CMS + Vit E 100 group	<0.001	

UCMS + vehicle group vs. CMS + GN50 group	<0.05	
UCMS + vehicle group vs. CMS + GN150 group	<0.05	
UCMS + vehicle group vs. CMS + GN450 group	<0.001	

Table S14.2 One-way analysis of variance (ANOVA) test of UCMS-Induced lipid peroxidation in hippocampus.

Group comparison	Statistical Analysis	
non-stress group vs. CMS + vehicle group	Paired *t-test*	
	P <0.001	
	ANOVA followed by Tukey's post hoc test	
	P	F ($DF_{between\ group}$, $DF_{residual}$
All group	<0.001	$F(4,16)=30.151$
UCMS + vehicle group vs. CMS + Vit E 100 group	<0.001	
UCMS + vehicle group vs. CMS + GN50 group	<0.001	
UCMS + vehicle group vs. CMS + GN150 group	<0.001	
UCMS + vehicle group vs. CMS + GN450 group	<0.001	
UCMS + GN 50 vs. UCMS + 450 group	0.008	
